# Psychological interventions for preventing relapse in individuals with partial remission of depression: a systematic review and individual participant data meta-analysis

**DOI:** 10.1017/S0033291725000157

**Published:** 2025-02-17

**Authors:** Joost Gülpen, Josefien J.F. Breedvelt, Eva A.M. van Dis, Gert J. Geurtsen, Fiona C. Warren, Cornelis van Heeringen, Caitlin Hitchcock, Fredrik Holländare, Marloes J. Huijbers, Robin B. Jarrett, Françoise Jermann, Margo de Jonge, Daniel N. Klein, Nicola S. Klein, S. Helen Ma, Michael T. Moore, Damiaan A.J.P. Denys, J. Mark G. Williams, Willem Kuyken, Claudi L. Bockting

**Affiliations:** 1 Amsterdam UMC, location University of Amsterdam, Department of Psychiatry, Amsterdam, The Netherlands; 2 Amsterdam Public Health Research Institute, Mental Health, Amsterdam, The Netherlands; 3 Amsterdam Neuroscience Research Institute, Amsterdam, The Netherlands; 4 Centre for Urban Mental Health, University of Amsterdam, Amsterdam, The Netherlands; 5 King’s College London, Department of Child and Adolescent Psychiatry, Institute for Psychiatry, Psychology and Neuroscience, London, UK; 6 Amsterdam UMC, location University of Amsterdam, Department of Medical Psychology, Amsterdam, The Netherlands; 7 Exeter Collaboration for Academic Primary Care (APEx), University of Exeter, Exeter, UK; 8 Department of Head and Skin, Faculty of Medicine and Health Sciences, Ghent University, Gent, Belgium; 9 Medical Research Council Cognition and Brain Sciences Unit, University of Cambridge, Cambridge, UK; 10 Melbourne School of Psychological Sciences, University of Melbourne, Melbourne, Victoria, Australia; 11 Department of Psychiatry, School of Medical Sciences, Örebro University, Örebro, Sweden; 12 Department of Psychiatry, Donders Center for Medical Neuroscience, Radboud University Medical Center, Nijmegen, The Netherlands; 13 University of Texas Southwestern Medical Center, Department of Psychiatry, Dallas, Texas, USA; 14 Department of Psychiatry, Geneva University Hospitals, Geneva, Switzerland; 15 Arkin Mental Health Institute, Amsterdam, The Netherlands; 16 Department of Psychology, Stony Brook University, Stony Brook, New York, USA; 17 GGZ Drenthe Mental Health Institute, Department Traumacentre, Beilen, The Netherlands; 18 Hong Kong Centre for Mindfulness, Hong Kong, China; 19 Gordon F. Derner School of Psychology, Adelphi University, Garden City, New York, USA; 20 Department of Psychiatry, University of Oxford, Oxford, UK

**Keywords:** Depression, Individual participant data meta-analysis, Major depressive disorder, Mood disorder, Partial remission, Psychological interventions, Quality of life, Relapse prevention, Residual symptoms, Treatment

## Abstract

Partial remission after major depressive disorder (MDD) is common and a robust predictor of relapse. However, it remains unclear to which extent preventive psychological interventions reduce depressive symptomatology and relapse risk after partial remission. We aimed to identify variables predicting relapse and to determine whether, and for whom, psychological interventions are effective in preventing relapse, reducing (residual) depressive symptoms, and increasing quality of life among individuals in partial remission. This preregistered (CRD42023463468) systematic review and individual participant data meta-analysis (IPD-MA) pooled data from 16 randomized controlled trials (*n* = 705 partial remitters) comparing psychological interventions to control conditions, using 1- and 2-stage IPD-MA. Among partial remitters, baseline clinician-rated depressive symptoms (*p* = .005) and prior episodes (*p* = .012) predicted relapse. Psychological interventions were associated with reduced relapse risk over 12 months (hazard ratio [HR] = 0.60, 95% confidence interval [CI] 0.43–0.84), and significantly lowered posttreatment depressive symptoms (Hedges’ *g* = 0.29, 95% CI 0.04–0.54), with sustained effects at 60 weeks (Hedges’ *g* = 0.33, 95% CI 0.06–0.59), compared to nonpsychological interventions. However, interventions did not significantly improve quality of life at 60 weeks (Hedges’ *g* = 0.26, 95% CI -0.06 to 0.58). No moderators of relapse prevention efficacy were found. Men, older individuals, and those with higher baseline symptom severity experienced greater reductions in symptomatology at 60 weeks. Psychological interventions for individuals with partially remitted depression reduce relapse risk and residual symptomatology, with efficacy generalizing across patient characteristics and treatment types. This suggests that psychological interventions are a recommended treatment option for this patient population.

## Introduction

Major depressive disorder (MDD) significantly affects overall mental and physical health (Gold et al., 2020; Herrman et al., 2022; Marx et al., 2023) and exerts strain on health services and society (Greenberg et al., 2021). This may be attributable to its high prevalence (Ferrari et al., 2013; Liu et al., 2020) and recurrent nature (Hardeveld et al., 2009), with relapse risk increasing with each major depressive episode (Hardeveld et al., 2009; Solomon, 2000). Although approximately half of previously depressed never experience a second depressive episode, for the others, it takes a recurrent course (Monroe & Harkness, 2022). Currently, antidepressant medication (ADM) is often the sole relapse prevention strategy used, despite persistently high relapse rates on ADM (Bockting et al., 2018; Kato et al., 2021). Moreover, patients prefer psychological treatment (McHugh et al., 2013), experience side effects from ADM (Anderson et al., 2012; Ferguson, 2001), and can resist prophylactic properties with long-term usage (Kaymaz et al., 2008), underscoring the limitations of relying solely on ADM. Hence, better relapse prevention in patients with recurrent depression is vital, and psychological interventions may play a key role.

One of the most robust prognostic indicators of relapse is partial remission of MDD (Böttcher et al., 2023; Buckman et al., 2018; Hardeveld et al., 2009; Judd et al., 1998; Paykel et al., 1995). Partial remission is defined as considerable residual depressive symptoms after a depressive episode (Frank et al., 1991; Paykel et al., 1995), such as depressed mood, suicidal ideation, and fatigue. Compared to full remission, partial remission is associated with considerable societal cost (Romera et al., 2010; Stewart et al., 2003; Tranter et al., 2002), a higher suicide risk (Sokero et al., 2005), diminished quality of life (Lenox-Smith et al., 2014; Riihimäki et al., 2016), and worse social functioning (Judd et al., 2000; Lenox-Smith et al., 2014; Romera et al., 2010). Clinical guidelines do not discern between fully or partially remitted patients (Gelenberg et al., 2010; NICE, 2022). However, this may be crucial to preventing relapse, as Kuyken et al. (2016) found symptom severity to moderate effectiveness of mindfulness-based cognitive therapy (MBCT) among 1258 remitted participants. Likewise, Jarrett et al. (2001) found that partial and unstable remitters especially benefited from the preventive effects of continuation cognitive therapy (C-CT). More personalized care by stratifying for symptom severity, instead of a one-size-fits-all approach, may enhance therapeutic outcomes.

How could partial remission best be treated? Our recent meta-analysis – the only one available specifically examining partial remission – demonstrated that psychological interventions show short-term benefits on depressive symptoms (Gülpen et al., 2023). However, these effects were not enduring at one-year follow-up, and importantly, psychological interventions did not reduce the relapse risk (Gülpen et al., 2023). The absence of effects is likely due to only three studies reporting longer-term outcomes and three studies examining relapse. Another explanation may be that interventions are effective for some individuals but not others. Such moderating effects were not systematically examined in included studies (Gülpen et al., 2023). Studying individual differences in effectiveness to elucidate what works for whom can help target preventive approaches or allow individuals to choose between treatment options.

To address the limited power in randomized controlled trials (RCTs) and previous meta-analytic research, and to examine individual differences in partial remission, an individual participant data meta-analysis (IPD-MA) approach is needed (Riley et al., 2021). First, while our previous meta-analysis included aggregate data from RCTs specifically targeting partial remitters (Gülpen et al., 2023), pooling individual participant data allows extraction of data on partial remitters within a higher number of RCTs that recruited both partial and full remitters, increasing statistical power. Second, IPD-MA overcomes aggregation bias and the large number of participant-level datapoints enables thorough assessment of individual-level predictors and moderators (Cuijpers et al., 2022). For example, using IPD-MA the International Taskforce for Relapse Prevention of Depression and Anxiety (ITFRA) demonstrated that the number of previous depressive episodes moderates relapse prevention outcomes (Breedvelt et al., 2024). Finally, IPD-MAs facilitate cross-sectional comparison of fully and partially remitted participants. Identifying participant-specific and intervention-specific variables associated with remission status can help identify barriers to achieving full remission and further support personalization. Earlier efforts did not specifically target partial remission and therefore what works (for whom) remains uncertain (Breedvelt et al., 2021, 2024; Kuyken et al., 2016).

In this systematic review and IPD-MA of RCTs using individual participant data from the ITFRA consortium, we aimed to (1) explore which clinical and demographic predictor variables are associated with prospectively assessed relapse among individuals in partial remission of MDD; (2) understand the effectiveness of psychological relapse prevention on depressive relapse risk, depressive symptoms, and quality of life; (3) examine which (if any) individual moderator characteristics, clinical, or demographic, are associated with differential treatment efficacy; and (4) compare fully and partially remitted participants at baseline.

## Method

### Search strategy

This IPD-MA (PROSPERO: CRD42023463468) was conducted according to the PRISMA-IPD (Supplementary Table 1) and Cochrane recommendations (Higgins et al., 2019; Stewart et al., 2015). Building on previous ITFRA initiatives (Breedvelt et al., 2020, 2024), we updated searches and focused on partial remission. Included were:(1) RCTs; (2) comparing psychological interventions versus any control conditions (e.g. treatment-as-usual [TAU], waitlist, or ADM); (3) in relapse prevention, with time to relapse established using clinical interviews (primary outcome); (4) in adult patients (mean age 18–65 years) in remission of MDD, defined as no or subthreshold symptoms for at least eight weeks measured with validated instruments. TAU constituted a control condition, wherein participants received the care they would typically receive outside the trial context, which could encompass receiving no care or ADM. Non-English publications and trials targeting conditions other than or in addition to depression (e.g. substance use disorders) were excluded. We updated searches in Cochrane, Embase, APA PsychInfo, and PubMed through October 18, 2023, using four search strings (Supplementary Appendix 1), expert input and references of meta-analyses. We defined partial remission of MDD, in line with previous research (Frank et al., 1991; Tranter et al., 2002), as (1) a previous MDD, yet no current episode based on a clinical interview or clinician-based assessment; (2) with at least considerable residual symptomatology remaining, as determined by a depression severity scale using a prespecified Hamilton Depression Rating Scale 17-item (HDRS-17) cutoff score of ≥8 (or transformed scores on alternative scales).

### Data collection, extraction, and handling

Authors of included studies were contacted to request data and, upon agreement, received a variable collection sheet stating instructions and requested variables (Supplementary Appendix 2). Shared data were recoded to harmonize and checked for accuracy using a two-step approach, verifying: (1) presence of variables and (2) whether data matched results and variables in published manuscripts (Supplementary Appendix 3). Authors were queried to resolve inconsistencies, confirm data, and request missing variables.

### Quality assessment

Three researchers independently rated the risk of bias (RoB) using the Cochrane Risk-of-Bias 2 (RoB-2) tool (Sterne et al., 2019), updating earlier assessments using version 1 (Higgins et al., 2011). Five domains were evaluated: (1) randomization process, (2) deviations from intended interventions, (3) missing outcome data, (4) outcome measurement, and (5) selection of reported results. Domains were judged at low RoB, having some concerns, or high risk, resulting in an overall evaluation using the RoB-2 algorithm. Assessments were piloted, registered (Supplementary Appendixes 4 and 5, for justifications and side-by-side comparisons of RoB-1/RoB-2) and disagreements resolved by senior authors.

### Data analysis

Analyses were conducted in Stata (v17) and R (v4.2.1), according to our preregistration (PROSPERO: CRD42023463468) and protocol (Breedvelt et al., 2020). Participants were classed as in partial or full remission, with partial remission operationalized using prespecified HDRS-17 scores of ≥8. When HDRS-17 scores were unavailable, depressive symptoms scores from alternative scales (e.g., BDI-II) were transformed, using Supplementary Table 2 (Furukawa et al., 2020; Rush et al., 2003). Partial remission prevalence and relapse rates were calculated in the total sample. Partial and full remitters were compared using χ^2^ tests for categorical variables and independent samples *t*-tests for continuous variables. Primary analyses used follow-up data to 12-months, censoring beyond this (Supplementary Appendix 6 for follow-up times and censoring). Participants with missing time-to-relapse data were excluded. Placebo or tapering groups were removed.

Treatment efficacy was determined using 1-stage (merging participant-level data across trials in single models), and 2-stage IPD-MA as our primary analysis (computing effects for each trial separately and then pooling effect sizes), with both random-effects and fixed-effects models for each approach. As ≥2 studies were required to conduct pairwise comparisons (Valentine et al., 2010), two clinically relevant comparisons were identified: (1) psychological versus nonpsychological interventions (primary comparison of interest) and (2) psychological interventions plus TAU versus TAU only (secondary comparison).

For 2-stage random-effects models, we used the DerSimonian and Laird method to pool individual results (DerSimonian & Laird, 1986), and applied the Hartung–Knapp–Sidik–Jonkman method to account for τ^2^ uncertainty (IntHout et al., 2014). Hazard ratios (HRs) for each study, pooled HRs, heterogeneity using *I*^2^, 95% confidence intervals (CIs), and forest plots were calculated based on 2-stage IPD-MA. Between-study variation due to heterogeneity, instead of sampling error, was examined using *I*^2^ and considered absent (0–24%), low (25–49%), moderate (50–74%), or high (75–100%) (Ioannidis et al., 2007). Fixed-effects models used inverse-variance weighting. 1-stage IPD-MA used Cox proportional hazard survival models for our primary outcome.

Depressive symptoms at posttreatment and 60-weeks follow-up (or closest available; range 52–65, mean = 58.2 weeks), and quality of life scores at 60-weeks, were standardized using *z*-score transformations. Again, 1- and 2-stage IPD-MA were used. In a 1-stage approach using multilevel models, standardized β-coefficients were calculated with participants clustered within trials. Here, higher β-coefficients indicate greater effects of treatment allocation on dependent variables. In 2-stage models, pooled effect sizes (Hedges’ *g*) were calculated, with positive Hedges’ *g* indicating treatment superiority, and interpreted as small (0.20–0.49), moderate (0.5–0.79), or large (≥0.8) (Cohen, 1988).

Based on the 2-stage IPD-MA approach, sensitivity analyses were conducted. As we found a substantial portion of participants (*n* = 134, 6.6%) to have baseline transformed HDRS-17 scores above the MDD cutoff (≥16), analyses excluding these patients were conducted, resulting in attenuated but comparable results (Supplementary Table 3). Small-study effects bias was investigated visually using funnel plots, tested and adjusted using Egger’s test of the intercept (Egger et al., 1997), and with Duval and Tweedie’s trim-and-fill procedure (Duval & Tweedie, 2000). Additionally, subgroup analyses – where possible – were performed for different treatment approaches, control conditions and study quality, using meta-regression and mixed-effect models.

To identify clinical and demographic predictors of relapse, predictors were entered in 1-stage survival models, using control-group data only. A study-level variable was included to account for patient clustering. Potential predictors were determined using two-sided *p*-values, with variables associated with time to relapse with *p* < .10. Variables were then combined in multivariable models, removing any variable with *p* > .05 to identify the final list of independent relapse predictors (Ahmed et al., 2014). For inclusion, predictor and moderator data had to be available for 60% of cases in ≥3 studies, and inclusion had to be justified based on prior literature identifying them as potentially relevant predictors or moderators (Breedvelt et al., 2020; Buckman et al., 2018).

Cox proportional-hazard assumptions were checked. Finally, moderation of all outcomes was investigated using 1-stage IPD-MA models, adding interaction terms between single moderating variables and binarized treatment allocations. In these models, interactions at the study level were estimated and pooled in random-effects IPD-MA. For models including interaction terms, predictors and moderators were centered around the study mean (continuous variables) or proportion *with* the covariate (binary variables), to facilitate interpretation and model estimation. In 1-stage IPD-MA interaction models, fixed effects on the study level (i.e., stratified by study) were added to avoid ecological bias, that is, possible discrepancies between group-level associations (across-trial) and individual-level associations (within) (Hua et al., 2017; Riley et al., 2010).

## Results

### Characteristics of studies

In total, 16,644 records, and 241 full-text articles were reviewed. Thirty RCTs (*n* = 4354) were identified, of which 20 agreed to share data (total *n* = 3171). Several author-stated factors contributed to an inability to share data (Supplementary Appendix 7), including time constraints and data protection regulations. Upon receipt of data, two studies had no primary outcome data (Biesheuvel-Leliefeld et al., 2017; Hoorelbeke et al., 2021), and two used active psychological comparisons (Farb et al., 2018; Shallcross et al., 2018).

**Figure 1.**
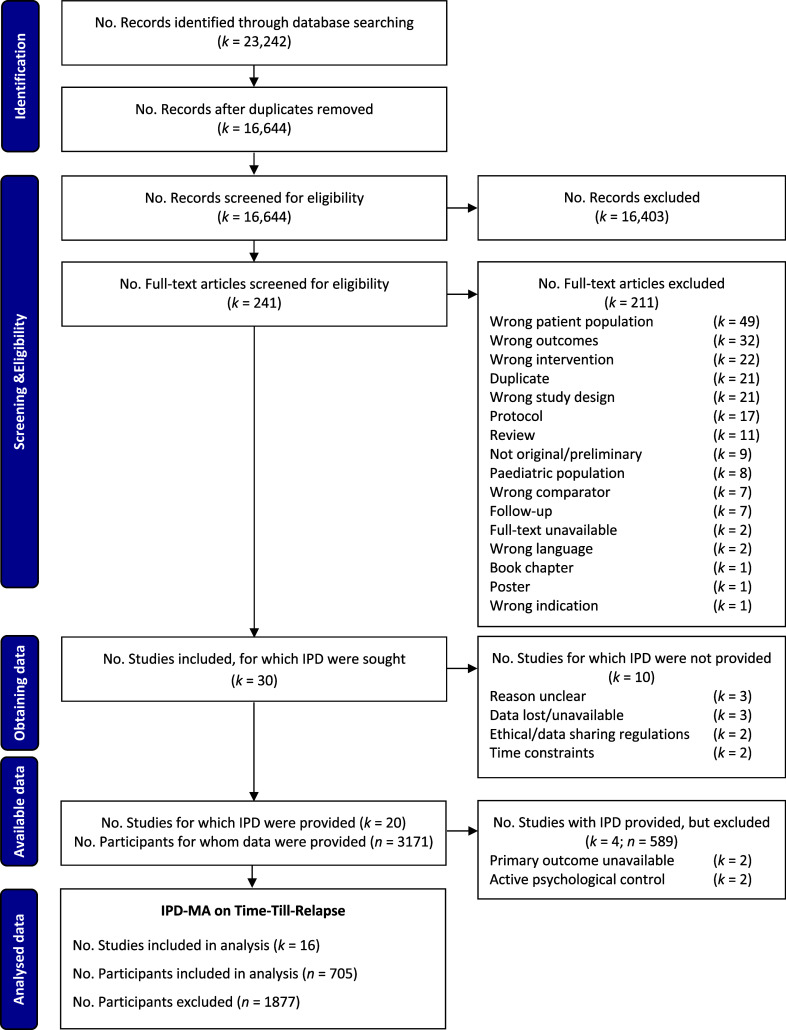
PRISMA Flow Diagram of Individual Participant Data Meta-Analysis Study Selection and Inclusion.

This resulted in 16 RCTs (Figure 1 and Supplementary Table 4) with complete outcome data at 12 months for 2028 participants (Bockting et al., 2005, 2018; Bondolfi et al., 2010; de Jonge et al., 2019; Godfrin & van Heeringen, 2010; Hitchcock et al., 2021; Holländare et al., 2011; Huijbers et al., 2015; Jarrett et al., 2001, 2013; Klein et al., 2004; Klein et al., 2018; Ma & Teasdale, 2004; Moore et al., 2022; Teasdale et al., 2000; Williams et al., 2014). Five psychological relapse prevention interventions were examined: cognitive behavioral therapy (CBT; *k* = 2), C-CT (*k* = 2), MBCT (*k* = 7), MemFlex (*k* = 1), and preventive cognitive therapy (PCT; *k* = 4). Control conditions consisted of TAU (*k* = 10), ADM (*k* = 3), psychoeducation (PE; *k* = 2), relaxation group therapy (RGT; *k* = 1), or evaluation only (*k* = 2). This led to two comparisons: (1) psychological versus nonpsychological interventions (*k* = 16) and (2) psychological interventions plus TAU versus TAU only (*k* = 8).

### Participant characteristics

The prevalence of partial remission of MDD was 34.8% (705/2028). Partial and full remitters were comparable on most variables (Table 1), but partial remitters more frequently identified as having a non-White background (*χ*^2^ = 9.86, *p* = .002), were more highly educated (*χ*^2^ = 5.53, *p* = .019), had longer durations of last depressive episodes (*t*(949) = 3.00, *p* = .003), experienced more psychiatric (*χ*^2^ = 7.97, *p* = .047), and physical comorbidity (*χ*^2^ = 5.23, *p* = .022), and showed lower therapy completion (*χ*^2^ = 41.20, *p* < .001). Moreover, partial remitters displayed a higher relapse rate at 12 months (*χ*^2^ = 32.70, *p* < .001), with 44.4% of partial versus 31.6% of full remitters relapsing. Examining the subset of partial remitters subsequently included in analyses, we found that on average they had depressive onset at age 25.9 years (SD = 12.20) and gone through 5.8 depressive episodes (SD = 8.01), with 77.5% having ≥3 episodes throughout their lifetime. Most previously received pharmacotherapy (64.5%) and psychological treatment (55.9%).

**Table 1.** Demographic and clinical characteristics, comparing patients in partial remission from MDD to those fully remitted

	Partial remission (*n* = 705)		Full remission (*n* = 1323)		***p* ^a^**
	Values	Total *n*	Values	Total *n*	
*Demographic characteristics*					
Age, years (SD)	45.06 (11.55)	703	44.44 (11.59)	1323	.257
Gender, female (%)	525 (74.57)	704	943 (71.28)	1323	.114
Ethnicity, White (%)	161 (84.74)	190	337 (93.09)	362	.002
Married/ cohabiting (%)	304 (46.55)	653	610 (47.47)	1285	.702
Employed (%)	197 (65.23)	302	556 (68.14)	816	.358
Education level completed, higher (%)	569 (85.18)	668	1007 (80.88)	1245	.019
*Clinical characteristics*					
Age of MDD onset, years (SD)	25.88 (12.20)	560	25.30 (11.75)	1155	.342
Previous episodes (SD)	5.84 (8.01)	623	5.35 (7.17)	1271	.177
Three or more episodes (%)	483 (77.53)	623	1002 (78.84)	1271	.516
Time in remission, months (SD)	12.94 (11.20)	99	13.06 (13.53)	460	.935
Duration last episode, months (SD)	18.23 (41.09)	246	12.20 (20.10)	705	.003
End of study relapse at 12-months (%)	313 (44.40)	705	418 (31.59)	1323	<.001
Psychiatric comorbidity		178		522	.047
No (%)	109 (61.24)		372 (71.26)		
Anxiety disorder (%)	46 (25.84)		87 (16.67)		
Other mental health disorder (%)	16 (8.99)		42 (8.05)		
Anxiety disorder and other disorder (%)	7 (3.93)		21 (4.02)		
Comorbid physical health disorder (%)	77 (60.63)	127	126 (48.28)	261	.022
Therapy sessions completed (%)	107 (61.49)	174	547 (83.77)	653	<.001
Previous psychological intervention (%)	167 (55.85)	299	344 (52.76)	652	.375
Previous pharmacotherapy (%)	129 (64.50)	200	114 (58.46)	195	.218
Depressive symptoms, HDRS–17^b^ (SD)	12.27 (4.14)	705	3.05 (2.27)	1323	<.001
Depressive symptoms, BDI-II (SD)	18.22 (8.23)	560	6.2 (6.09)	756	<.001

*Note*: Data are mean (SD), *n*, or %.

*Abbreviations: BDI = Beck Depression Inventory; HDRS-17 = 17-item Hamilton Depression Rating Scale; MDD = major depressive disorder; SD = standard deviation.*

a χ^2^-tests for categorical variables and independent samples *t*-tests for continuous variables.

b HDRS-17 scores are transformed from other scales when unavailable.

### Predictors of relapse

In examining prospective relapse predictors among partial remitters (Supplementary Table 5), both clinician-rated (HR = 1.05, 95% CI 1.01–1.09, *p* = .016) and self-reported depressive symptoms (HR = 1.02, 95% CI 1.00–1.04, *p* = .088), and ≥3 prior depressive episodes (HR = 1.63, 95% CI 1.03–2.58, *p* = .036), were significantly associated with an increased relapse risk at 12 months at *p* < .10. Clinician-rated depressive symptoms (HR = 1.22, 95% CI 1.06–1.40, *p* = .005) and ≥3 prior depressive episodes (HR = 2.17, 95% CI 1.18–3.98, *p* = .012) remained significant at *p* < .05 when added in a multivariable model.

### Treatment efficacy on preventing relapse

**Figure 2.**
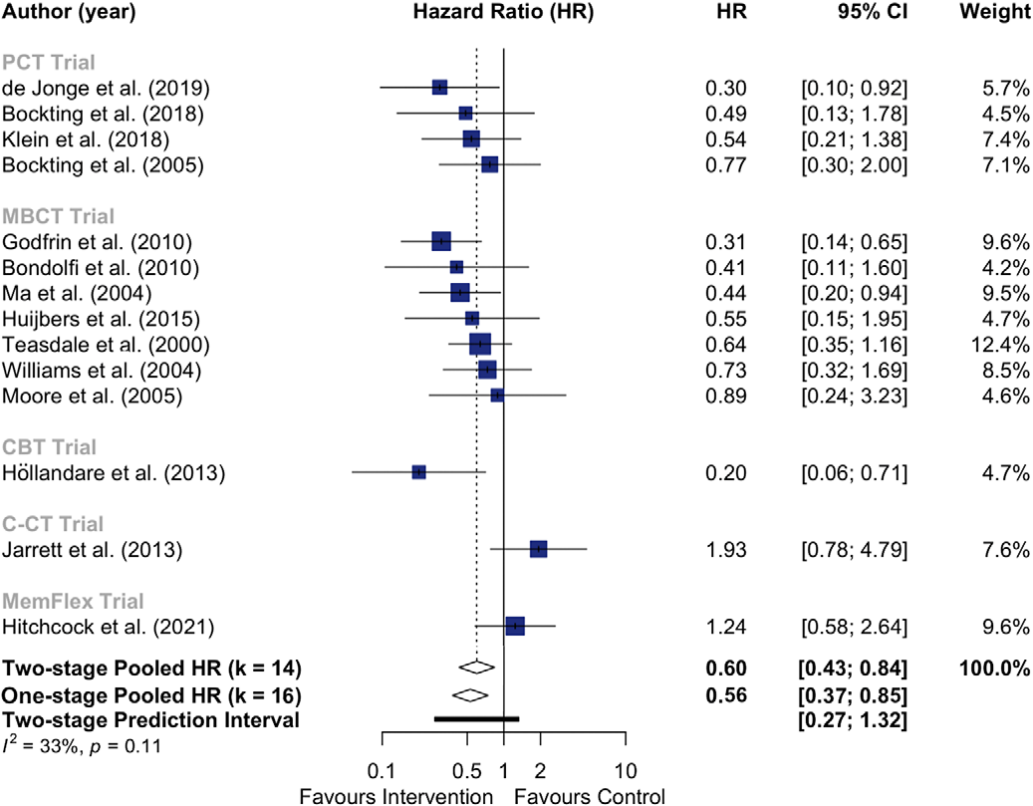
Forest Plot of Random Effects Two-Stage Individual Participant Data Meta-Analysis for Time-to-relapse, Comparing Psychological with Non-psychological Control Interventions. Note. Jarrett et al. (2001) and Klein et al. (2004) were excluded from the two-stage individual participant data meta-analysis models, as there were too few relapses observed. All 16 studies were pooled in the one-stage IPD-MA individual participant data meta-analysis depicted. CBT = cognitive behavioural therapy; C-CT = continuation cognitive therapy; MBCT = mindfulness-based cognitive therapy; PCT = preventive cognitive therapy.

Examining preventive effects using 2-stage random-effects IPD-MA, psychological interventions were associated with a significantly longer time to relapse, compared to nonpsychological control (Figure 2 and Table 2; HR = 0.60, 95% CI 0.43–0.84, *p* = .006, *I*^2^ = 32.7%, *n* = 672, *k* = 14), with 6.7 weeks difference in median survival time. Similarly, psychological interventions with TAU was associated with superior effects on time to relapse compared to TAU alone (HR = 0.51, 95% CI 0.32–0.73, *p* = .003, *I*^2^ = 0.0%, *n* = 402, *k* = 8). Overall, results from 1-stage IPD-MA were similar (Table 2). No significant moderator effects were observed for either comparison using 1-stage IPD-MA models (Supplementary Table 6). Using subgroup analyses, no differences in effects were found based on intervention type (*p*_subgroup_ = .839) or control conditions (*p*_subgroup_ = .069). While visual inspection of the funnel plot showed minor asymmetry as evidence of small-study effects bias (Supplementary Figure 2), Egger’s test did not (*p* = .479). Duval and Tweedie’s trim-and-fill procedure attenuated the effect (HR = 0.72, 95% CI 0.50–1.04, *p* = .078), indicating that it was overestimated, possibly due to small-study effects bias.

**Table 2:** 1- and 2-stage IPD meta-analysis models of treatment efficacy on time to relapse at 12-months

Comparisons and models	HR (95% CI)	*P*	*n* participants (relapsed)	*k* studies
*Psychological interventions versus nonpsychological control conditions*				
1-stage random-effects model	0.56 (0.37–0.85)	.006	701 (313)	16
1-stage fixed-effects model	0.58 (0.46–0.73)	<.001	701 (313)	16
2-stage random-effects model	0.60 (0.43–0.84)	.006	672 (309)	14^a^
2-stage fixed-effects model	0.61 (0.48–0.78)	<.001	672 (309)	14^a^
*Psychological interventions plus TAU versus TAU only*				
1-stage random-effects model	0.51 (0.39–0.67)	<.001	402 (205)	8
1-stage fixed-effects model	0.50 (0.38–0.67)	<.001	402 (205)	8
2-stage random-effects model	0.51 (0.32–0.73)	.003	402 (205)	8
2-stage fixed-effects model	0.51 (0.38–0.69)	<.001	402 (205)	8

a Jarrett et al. (2001) and Klein et al. (2004) were excluded from the 2-stage models, as there were too few relapses observed.

### Treatment efficacy on depressive symptoms and quality of life

Examining effects on depressive symptoms, using 2-stage random-effects IPD-MA, resulted in superior treatment outcomes for psychological interventions versus nonpsychological controls (Table 3 and Supplementary Figure 1), both at posttreatment (Hedges’ *g* = 0.29, 95% CI 0.04–0.54, *p* = .025, *I*^2^ = 43.0%, *n* = 600, *k* = 15) and 60-week follow-up (Hedges’ *g* = 0.33, 95% CI 0.06–0.59, *p* = .020, *I*^2^ = 38.2%, *n* = 544, *k* = 14). Again, results from 1-stage IPD-MA were similar (Table 3). Comparing psychological intervention plus TAU versus TAU only, pooled effect sizes using 2-stage models were small and nonsignificant at posttreatment (Hedges’ *g* = 0.43, 95% CI -0.01–0.88, *p* = .056, *I*^2^ = 61.2%, *n* = 353, *k* = 7) and 60-week follow-up (Hedges’ *g* = 0.40, 95% CI -0.09–0.89, *p* = .092, *I*^2^ = 60.1%, *n* = 338, *k* = 7). However, 1-stage outcomes were significant at posttreatment and 60 weeks (mean *β* = −0.53, *p* < .001, mean *β* = −0.45, *p* < .001, respectively). Effects on quality of life were nonsignificant for both IPD-MA models and no further moderator analyses were conducted, due to the limited data available (*n* = 196, *k* = 7; Table 3).

**Table 3.** Results from one- and two-stage IPD meta-analysis models of treatment efficacy on depression severity and quality of life, following partial remission of major depressive disorder

	1-stage IPD-MA			2-stage IPD-MA			
Variable and comparison	*n* observations (*k* studies)	Mean *β* (SE)^a^	*p*	Hedges’ *g* (95% CI)	*p*	***I***^**2**^	95% PI
*Psychological interventions versus nonpsychological control*							
Depression severity posttreatment	600 (15)	−0.39 (0.10)	<.001	0.29 (0.04–0.54)	.025	43.0%	−0.39–0.98
Depression severity 60-week follow-up	544 (14)	−0.35 (0.10)	<.001	0.33 (0.06–0.59)	.020	38.2%	−0.31–0.97
Quality of life 60-week follow-up	196 (7)	0.21 (0.50)	.675	0.26 (−0.06–0.58)	.090	0.0%	−0.12–0.64
*Psychological interventions plus TAU versus TAU only*							
Depression severity posttreatment	353 (7)	−0.53 (0.12)	<.001	0.43 (−0.01–0.88)	.056	61.2%	−0.67–1.54
Depression severity 60-week follow-up	338 (7)	−0.45 (0.13)	<.001	0.40 (−0.09–0.89)	.092	60.1%	−0.69–1.50
Quality of life 60-week follow-up	56 (2)	0.01 (0.02)	.563	n.s.^b^			

Abbreviations: CI = confidence interval; n.s. = not specified; PI = prediction interval; vs = versus.

a Standardized β coefficients using depression severity scores from the 17-item version Hamilton Depression Rating Scale, Montgomery Åsberg Depression Rating Scale, Inventory of Depressive Symptomatology – Clinician, Beck Depression Inventory I or II.

b Undefined as less than three studies with data available in this comparison.

Using moderator analyses to study differential treatment efficacy on depressive symptoms (Supplementary Tables 7 and 8), marital status significant interacted with efficacy at posttreatment in our first (psychological versus nonpsychological interventions; mean *β* = −0.49, *p* = .002) and second comparison (psychological intervention plus TAU versus TAU only; mean *β* = −0.59, *p* = .004). Thus, psychological interventions for patients in partial remission – compared to controls – seemed more effective in reducing depressive symptoms at posttreatment for those single, divorced, widowed, or separated. Furthermore, male patients (mean *β* = −0.52, *p* = .009), older patients (mean *β* = −0.01, *p* = .048), and patients with higher clinician-rated baseline depressive symptom severity (mean *β* = −0.05, *p* = .047), had fewer depressive symptoms at 60 weeks when receiving psychological interventions. In our second comparison, higher clinician-rated (mean *β* = −0.06, *p* = .017) and self-reported baseline depressive symptoms (mean *β* = −0.03, *p* = .027), and male gender (mean *β* = −0.71, *p* = .007), were associated with superior efficacy of psychological interventions.

Neither funnel plots (Supplementary Figures 3 and 4) nor Egger’s test provided evidence for small-study effects bias at posttreatment or 60-week follow-up (*p* = .423 and *p* = .361, respectively). Duval and Tweedie’s trim-and-fill procedure only slightly changed results at posttreatment (Hedges’ *g* = 0.33, 95% CI 0.07–0.59, *k* = 1 added), but not at 60 weeks (Hedges’ *g* = 0.33, 95% CI 0.06–0.59). Comparing treatment or control conditions was not possible due to a lack of power.

### Quality assessment

Overall, risk of bias was considerable (RoB-2 plot in Supplementary Figure 5). Three RCTs were assessed at low risk, three had some concerns, and 10 were at high risk. Preregistrations, protocols, or statistical analysis plans dated prior to study completion were obtained for eight studies. Differences in relapse prevention effects were found for risk of bias (*p*_subgroup_ = .006), with superior effects in high-risk studies (HR = 0.48, 95% CI 0.34–0.66, *p* = .001, *k* = 8), compared to studies having some concerns (HR = 0.49, 95% CI 0.14–1.78, *p* = .140, *k* = 3) or at low risk (HR = 1.18, 95% CI 0.37–3.76, *p* = .596, *k* = 3). Likewise, effects on depressive symptomatology differed between studies based on their RoB at posttreatment (*p*_subgroup_ = .016) and 60-week follow-up (*p*_subgroup_ = .002), with larger effects for higher-risk studies.

## Discussion

This IPD-MA of 16 RCTs showed psychological interventions for partial remission of MDD, compared to control conditions, effectively reduce (residual) depressive symptoms immediately after treatment and at 60-week follow-up. Critically, psychological interventions extended depressive time to relapse over 12 months but did not improve quality of life more than control conditions. Interventions are more effective than control conditions in reducing long-term depressive symptoms for individuals with higher baseline symptomatology in partial remitted MDD, indicating they work even better for those most at-risk. We found no evidence that individual characteristics or treatment types have differential effects on relapse prevention. These findings extend our prior meta-analysis of treatment for partial remission (Gülpen et al., 2023), including more studies and using participant-level data increasing statistical power. Despite prior reports of worse outcomes for partial remitters (Paykel, 2008; Tranter et al., 2002), clinical guidelines and studies have not consistently differentiated them from those fully recovered (Gelenberg et al., 2010; NICE, 2022). Our study provides compelling evidence for the advantages and generalizability of psychological interventions for individuals with partially remitted MDD. Therefore, it may be valuable for guidelines to include recommendations specifically for this at-risk group. As no differences between treatment types were observed, results indicate that partially remitted individuals have several effective treatments available to choose from (8 weekly sessions MBCT or PCT; 6–10 sessions CBT; 4 weeks MemFlex; or 10 sessions C-CT over 8 months).

Importantly, the favorable outcomes of psychological interventions were found despite partial remitters’ particular susceptibility to relapse (Buckman et al., 2018; Hardeveld et al., 2009; Paykel et al., 1995). Over 12 months, we found that 44.4% relapsed, compared to 31.6% in the fully remitted group. Among partially remitted individuals in our study, baseline depressive symptoms and the number of prior episodes are associated with a significantly higher relapse risk. This further highlights the relative importance of clinical prognostic factors compared to demographic factors in predicting relapse (Buckman et al., 2018; Hardeveld et al., 2009). Overall, psychological interventions – alone or added to TAU – were, relative to control conditions, associated with a longer time to relapse over 12 months in partial remission of MDD. Preventive outcomes were comparable to those of previous IPD-MAs and meta-analyses (Biesheuvel-Leliefeld et al., 2015; Breedvelt et al., 2024; Clarke et al., 2015; Kuyken et al., 2016). Consistent with this work, we found no differences in efficacy between treatment types. Additionally, we did not find support for differential treatment effects based on age, gender, education level, marital status, age of onset, number of prior depressive episodes, or baseline symptom severity. This indicates their efficacy generalizes across these individual characteristics in partially remitted individuals. Individuals thus have a choice between treatment types, regardless of patient factors. This choice applies to individuals with one or two prior episodes, but also those with ≥3 previous depressive episodes typically considered at increased relapse risk.

Furthermore, our findings indicate the benefit of psychological interventions in reducing residual depressive symptoms immediately posttreatment and at 60-weeks follow-up. However, when comparing psychological interventions plus TAU versus TAU only, results differed between 1- and 2-stage models. Effects on symptomatology were significant using 1-stage, while borderline nonsignificant for 2-stage models. However, most trials are relatively small and we observed moderate heterogeneity using 2-stage models, conceivably leading to underestimation of effects. Additionally, what constitutes TAU varied across trials, often comprising ADM continuation (Supplementary Table 4), though detailed accounts are lacking (Petersson et al., 2023). This lack of clarity may hinder interpretability, replicability, and reliability of findings. In the context of relapse prevention, TAU commonly consists of ADM continuation or no care at all (de Jonge et al., 2019). Interestingly, marital status interacted with posttreatment effects on depressive symptoms. Single, widowed, and separated individuals experienced greater efficacy. This group has previously been found to have a worse depression prognosis (Buckman et al., 2021), possibly allowing more room for improvement among single or no longer married individuals in our study, leading to enhanced treatment outcomes. Our finding that psychological interventions are more effective in reducing depressive symptoms at 60 weeks for partially remitted individuals, at higher baseline levels of depressive symptoms (HDRS-17 cutoff around 16), is of importance as this highlights that interventions work best for those most affected. In contrast, IPD-MAs on depression found baseline severity to have little impact on symptomatology outcomes after treatment (Bower et al., 2013; Furukawa et al., 2017). Current findings do hint effects are different for partial remitters, with superior effects for those with higher levels of symptomatology.

No differences were observed for quality of life, contrasting positive results previously reported (Kolovos et al., 2016; Segal et al., 2020). However, our results should be interpreted with caution as data was only sparsely available for this outcome (*n* = 196, *k* = 4), changes in quality of life may take longer to manifest compared to depressive symptomatology (McKnight & Kashdan, 2009), and studies varied in their conceptual measurement of quality of life (Supplementary Table 4). Four studies used the EQ-5D to measure quality of life, while this instrument is criticized as insensitive to change in mental health populations (van Kaam et al., 2024). Additionally, although these analyses were planned, the lack of sufficient power precluded a robust moderation analysis for quality of life.

The current study, done within a systematic review framework, is the first utilizing IPD-MA for evidence synthesis to examine outcomes specifically for those experiencing partial remission of MDD. A key strength lies in the data consolidation – collected by different international groups – not previously published separately for the partial remission subgroup. With this, it helps bridge critical knowledge gaps without resorting to additional resource-intensive trials. Finally, our rigorous data checking provided independent scrutiny of data and ensured its quality, completeness, and consistency.

Nonetheless, some limitations deserve mentioning. First, while based on a predefined and empirical cutoff score established by earlier consensus (Tranter et al., 2002), we selected a partial remission subsample. This post hoc analysis of RCTs may introduce selection bias, as selecting this subsample may be influenced by unknown characteristics and is not random. RCTs included individuals in remission and did not specifically target those only partially remitted. One’s symptom severity may affect participation in prevention trials, thereby hampering the generalizability of our findings. Relatedly, some RCTs imposed upper limits on depression severity as inclusion criteria, while others did not, introducing heterogeneity (though we were able to explicitly measure between-study heterogeneity). Second, our review included RCTs examining time to relapse and data for this variable was requested. Studies examining effects on symptomatology or quality of life, without primary outcome data available, were excluded. This may hamper the robustness of results for these outcomes, as such trials are known. Third, we were unable to conduct sensitivity analyses comparing studies providing data to those that did not, as only partial remitters were selected and aggregate data are not commonly published separately for this subgroup, potentially introducing availability bias. Fourth, we relied on a single measurement of depressive symptoms to stratify participants as partial versus full remitters. Given the considerable intraindividual variability in severity over time it would be prudent to use multiple measurements over time to determine remission status and stability. Nevertheless, real-world clinical decision-making would likely also be driven by a single measurement, perhaps including retrospective reporting. Fifth, we could not include all psychological modalities. Importantly, the absence of evidence for modalities not included, for example, interpersonal psychotherapy (no data provided), does not equal evidence of ineffectiveness. Finally, we were unable to examine some relevant factors, including prior psychological or pharmacological treatment during the acute phase of MDD. Future studies should consistently explore and report this to allow for more personalization and potentially staged treatment.

### Conclusions

This first-ever IPD-MA examining psychological interventions – specifically for individuals in partial remission of unipolar depression – finds support for their effectiveness in reducing relapse risk as well as residual symptomatology. Crucially, findings underscore that these psychological interventions should be offered to partially remitted individuals, ensuring patient involvement through shared decision-making, as diverse interventions work regardless of patient characteristics. Despite its limitations, this study addresses critical gaps in our understanding of treatment efficacy for partial remission without the need for additional resource-intensive RCTs. Notably, it may provide clinical guidance for treatment decisions in patients that have remitted. Future research should validate these findings, considering additional factors such as dose–response relationships or prior treatment. While research has repeatedly found psychological relapse prevention interventions effective, our study provides the most compelling empirical evidence to date of its effectiveness for individuals in partial remission of MDD, further suggesting they have even superior effects for those with the poorest prognosis (i.e., higher levels of residual symptomatology).

## Supporting information

Gülpen et al. supplementary material Gülpen et al. supplementary material

## Supplementary material

To view supplementary material for this article, please visit http://doi.org/10.1017/S0033291725000157.
