# Supplementary material for: Psychological interventions for preventing relapse in individuals with partial remission of depression: a systematic review and individual participant data meta-analysis
Source: Psychol Med. 2025 Feb 17;55:e50. doi: 10.1017/S0033291725000157 (PMC12080647; doi:10.1017/S0033291725000157)

**Supplementary Online Content**

[Supplementary Appendix 1. Four different search string and terms used, per database searched. 2](#_Toc164421628)

[Supplementary Appendix 2. Variable collection sheet. 5](#_Toc164421629)

[Supplementary Appendix 3. Overview of data wrangling (extracting, checking and recoding) upon receipt, per study. 8](#_Toc164421630)

[Supplementary Appendix 4. Side-by-side comparisons of RoB-1 and RoB-2 final judgements. 10](#_Toc164421631)

[Supplementary Appendix 5. Overview of risk of bias judgement and supportive descriptions, based on the RoB-2 tool 11](#_Toc164421632)

[Supplementary Appendix 6. Overview of follow-up times in included studies and censoring of data conducted. 46](#_Toc164421633)

[Supplementary Appendix 7. Studies with IPD not provided and reasons given. 47](#_Toc164421634)

[Supplementary Table 1. PRISMA-IPD Checklist. 48](#_Toc164421635)

[Supplementary Table 2. Depression symptom severity transformation table used for values available. 52](#_Toc164421636)

[Supplementary Table 3. Sensitivity analysis excluding patients with HDRS-17 scores of 16 and above, on the primary outcome of time-to-relapse at 12 months. 54](#_Toc164421637)

[Supplementary Table 4. Characteristics of included studies. 55](#_Toc164421638)

[Supplementary Table 5. Demographic and clinical variables as predictor of relapse in the control group. 57](#_Toc164421639)

[Supplementary Table 6. Moderator analysis of treatment efficacy on time-to-relapse at 12 months, following partial remission from major depressive disorder. 58](#_Toc164421640)

[Supplementary Table 7. Moderator analysis of treatment efficacy on depressive symptom severity at post-treatment, following partial remission of major depressive disorder. 59](#_Toc164421641)

[Supplementary Table 8. Moderator analysis of treatment efficacy on depressive symptom severity at 60 weeks, following partial remission of major depressive disorder. 60](#_Toc164421642)

[Supplementary Figure 1. Forest Plot of Random Effects Two-Stage Individual Participant Data Meta-Analysis for Depression Severity at Post-treatment and 60-weeks Follow-up, Comparing Psychological with Non-psychological Interventions. 61](#_Toc164421643)

[Supplementary Figure 2. Funnel plot examining small-study effects bias with results from a two-stage IPD meta-analysis model of treatment efficacy on time-to-relapse 12 months. 62](#_Toc164421644)

[Supplementary Figure 3. Funnel plot examining small-study effects bias with results from a two-stage IPD meta-analysis model of treatment efficacy on depressive symptom severity at post-treatment. 63](#_Toc164421645)

[Supplementary Figure 4. Funnel plot examining small-study effects bias with results from a two-stage IPD meta-analysis model of treatment efficacy on depressive symptom severity at 60-weeks follow-up. 64](#_Toc164421646)

[Supplementary Figure 5. Risk of bias plot using the Cochrane Risk-of-Bias 2 tool of included randomized controlled trials, across 5 domains and overall. 65](#_Toc164421647)

This supplemental material has been provided by the authors to give readers additional information about their work.

# Supplementary Appendix 1. Four different search string and terms used, per database searched.

*PUBMED:*

*#1:* (((("depressive disorder"[MeSH Terms] OR "depression"[MeSH Terms] OR depress*[Title/Abstract] OR affective disorder[Title/Abstract] OR affective disorders[Title/Abstract] OR dysphoria[Title/Abstract] OR dysthymia[Title/Abstract] OR depressed mood[Title/Abstract]) OR mood disorder[Title/Abstract])))

*#2:* (("recurrence"[MeSH Terms]) OR ("recur*"[Title/Abstract]) OR ("chroni*"[Title/Abstract]) OR (relaps*[Title/Abstract]) OR ("remi*"[Title/Abstract]) OR ("treatment resistant"[Title/Abstract]))) OR (("recurrent depression"[MeSH Terms]) OR ("treatment resistant depression"[Title/Abstract]))

*#3:* ("secondary prevention"[MeSH Terms] OR "prevention"[MeSH] OR "preventive [MeSH Terms]" OR "maintenance"[Title/Abstract]) OR ("continuation"[Title/Abstract]) OR ("prophyla*"[Title/Abstract])

*#4:* (("randomized controlled trial"[Publication Type] OR controlled clinical trial[Title/Abstract] OR random*[Title/Abstract] OR prevention and control[Title/Abstract]))

*#5:* 2021/01/22:2023/07/19[crdt]

*#6:* #1 AND #2 AND #3 AND #4 AND #5

*APA PSYCHINFO (OVIDSP):*

*1:* (randomized controlled trial or controlled clinical trial or randomly).mp. or exp clinical trials/ or random*.mp. or (prevention and control).mp. or randomised.mp. or randomized.mp. [mp=title, abstract, heading word, table of contents, key concepts, original title, tests & measures, mesh]

*2:* exp "Relapse (Disorders)"/ or exp "Remission (Disorders)"/ or exp "symptom remission"/ or recur*.ab,ti. or chroni*.ab,ti. or relaps*.ab,ti. or remi*.ab,ti. or treatment resistan*.ab,ti.

*3:* exp Treatment Resistant Depression/ or exp Recurrent Depression/ or exp Chronic Mental Illness/

*4:* major depression/ or "depress*".ab,ti. or affective disorder*.ab,ti. or dysphoria,ab.ti. or depressed mood.ab,ti. or mood disorder.ab,ti. or dysthymia.ab,ti.

*5:* 2 and 4

*6:* 3 or 5

*7:* exp Prevention/ or exp Maintenance Therapy/ or secondary prevention.mp. or preventive.ab,ti. or continuation.ab,ti. or prophyla*.ab,ti. or aftercare/ or intervention.ab,ti.

*8:* 1 and 6 and 7

*9:* limit 8 to up=20210122-20230719

*EMBASE (OVIDSP):*

*1:* treatment resistant depression.mp. [mp=title, abstract, heading word, drug trade name, original title, device manufacturer, drug manufacturer, device trade name, keyword, floating subheading word, candidate term word]

*2:* exp treatment resistant depression/

*3:* depression.mp. [mp=title, abstract, heading word, drug trade name, original title, device manufacturer, drug manufacturer, device trade name, keyword, floating subheading word, candidate term word]

*4:* depress*.mp. [mp=title, abstract, heading word, drug trade name, original title, device manufacturer, drug manufacturer, device trade name, keyword, floating subheading word, candidate term word]

*5:* affective disorder.mp. [mp=title, abstract, heading word, drug trade name, original title, device manufacturer, drug manufacturer, device trade name, keyword, floating subheading word, candidate term word]

*6:* exp depression/

*7:* affective disorder*.mp. [mp=title, abstract, heading word, drug trade name, original title, device manufacturer, drug manufacturer, device trade name, keyword, floating subheading word, candidate term word]

*8:* exp dysphoria/

*9:* dysphoria.mp. [mp=title, abstract, heading word, drug trade name, original title, device manufacturer, drug manufacturer, device trade name, keyword, floating subheading word, candidate term word]

*10:* exp dysthymia/

*11:* dysthymia.mp. [mp=title, abstract, heading word, drug trade name, original title, device manufacturer, drug manufacturer, device trade name, keyword, floating subheading word, candidate term word]

*12:* depressed mood.mp. [mp=title, abstract, heading word, drug trade name, original title, device manufacturer, drug manufacturer, device trade name, keyword, floating subheading word, candidate term word]

*13:* exp mood disorder/

*14:* mood disorder.mp. [mp=title, abstract, heading word, drug trade name, original title, device manufacturer, drug manufacturer, device trade name, keyword, floating subheading word, candidate term word]

*15:* 1 or 2 or 3 or 4 or 6 or 7 or 8 or 9 or 10 or 11 or 12 or 13 or 14

*16:* exp recurrent disease/

*17:* recurrent disease.mp. [mp=title, abstract, heading word, drug trade name, original title, device manufacturer, drug manufacturer, device trade name, keyword, floating subheading word, candidate term word]

*18:* exp relapse/

*19:* relapse.mp. [mp=title, abstract, heading word, drug trade name, original title, device manufacturer, drug manufacturer, device trade name, keyword, floating subheading word, candidate term word]

*20:* exp chronic depression/

*21:* chronic depression.mp. [mp=title, abstract, heading word, drug trade name, original title, device manufacturer, drug manufacturer, device trade name, keyword, floating subheading word, candidate term word]

*22:* remitted.mp. [mp=title, abstract, heading word, drug trade name, original title, device manufacturer, drug manufacturer, device trade name, keyword, floating subheading word, candidate term word]

*23:* exp remission/

*24:* remission.mp. [mp=title, abstract, heading word, drug trade name, original title, device manufacturer, drug manufacturer, device trade name, keyword, floating subheading word, candidate term word]

*25:* recur*.mp. [mp=title, abstract, heading word, drug trade name, original title, device manufacturer, drug manufacturer, device trade name, keyword, floating subheading word, candidate term word]

*26:* relaps*.mp. [mp=title, abstract, heading word, drug trade name, original title, device manufacturer, drug manufacturer, device trade name, keyword, floating subheading word, candidate term word]

*27:* chroni*.mp. [mp=title, abstract, heading word, drug trade name, original title, device manufacturer, drug manufacturer, device trade name, keyword, floating subheading word, candidate term word]

*28:* 16 or 17 or 18 or 19 or 20 or 21 or 22 or 23 or 24 or 25 or 26 or 27

*29:* exp secondary prevention/

*30:* secondary prevention.mp. [mp=title, abstract, heading word, drug trade name, original title, device manufacturer, drug manufacturer, device trade name, keyword, floating subheading word, candidate term word]

*31:* exp prevention/

*32:* prevention.mp. [mp=title, abstract, heading word, drug trade name, original title, device manufacturer, drug manufacturer, device trade name, keyword, floating subheading word, candidate term word]

*33:* maintenan*.mp. [mp=title, abstract, heading word, drug trade name, original title, device manufacturer, drug manufacturer, device trade name, keyword, floating subheading word, candidate term word]

*34:* prophyla*.mp. [mp=title, abstract, heading word, drug trade name, original title, device manufacturer, drug manufacturer, device trade name, keyword, floating subheading word, candidate term word]

*35:* continuation.mp. [mp=title, abstract, heading word, drug trade name, original title, device manufacturer, drug manufacturer, device trade name, keyword, floating subheading word, candidate term word]

*36:* 29 or 30 or 31 or 32 or 33 or 34 or 35

*37:* exp randomized controlled trial/

*38:* randomized controlled trial.mp. [mp=title, abstract, heading word, drug trade name, original title, device manufacturer, drug manufacturer, device trade name, keyword, floating subheading word, candidate term word]

*39:* exp controlled clinical trial/

*40:* controlled clinical trial.mp. [mp=title, abstract, heading word, drug trade name, original title, device manufacturer, drug manufacturer, device trade name, keyword, floating subheading word, candidate term word]

*41:* random*.mp. [mp=title, abstract, heading word, drug trade name, original title, device manufacturer, drug manufacturer, device trade name, keyword, floating subheading word, candidate term word]

*42:* random*.mp. [mp=title, abstract, heading word, drug trade name, original title, device manufacturer, drug manufacturer, device trade name, keyword, floating subheading word, candidate term word]

*43:* (prevention and control).mp. [mp=title, abstract, heading word, drug trade name, original title, device manufacturer, drug manufacturer, device trade name, keyword, floating subheading word, candidate term word]

*44:* (prevention and control).mp. [mp=title, abstract, heading word, drug trade name, original title, device manufacturer, drug manufacturer, device trade name, keyword, floating subheading word, candidate term word]

*45:* 37 or 38 or 39 or 40 or 42 or 43 or 44

*46:* 15 and 28 and 36 and 45

*47:* limit 46 to yr="2019 -Current"

*48:* limit 46 to (in-process status and yr="2019 -Current" and (article or article in press or conference abstract or "conference review" or "review"))

limit 47 to dd=20210122-20230719

*COCHRANE*:

*#1:* "depression"

*#2:* depress*

*#3:* "affective"

*#4:* affective disorders

*#5:* dysphoria

*#6:* dysthymia

*#7:* "mood disorder"

*#8:* "depressed mood"

*#9:* "recur*"

*#10:* "chroni*"

*#11:* "relaps*"

*#12:* "remi*"

*#13:* "treatment resistant"

*#14:* "secondary prevention study"

*#15:* "prevention"

*#16:* "maintenance"

*#17:* "prophylaxis"

*#18:* "continuation"

*#19:* "randomised control trial"

*#20:* "randomized controlled trial"

*#21:* "controlled clinical trial"

*#22:* "prevention and control"

*#23:* random*

*#24:* #1 or #2 or #3 or #4 or #5 or #6 or #7 or #8

*#25:* #9 or #10 or #11 or #12 or #13

*#26:* #14 or #15 or #16 or #17 or #18

*#27:* #19 or #20 or #21 or #23

*#28:* #24 and #25 and #26 and #27 with Cochrane Library publication date Between Jan 2021 and July 2023

# Supplementary Appendix 2. Variable collection sheet.

**VARIABLE COLLECTION SHEET**

**INDIVIDUAL PARICIPANT DATA ANALYSIS ON RELAPSE PREVENTION FOR DEPRESSION**

*Study name:*

*Collaborating author:*

*Email:*

**INSTRUCTIONS**

*Please supply the following information and data (where available). We also require a data key and codebook or coding labels for included variables to permit correct re-coding.*

*Please provide the dataset with missing values, not imputed values. Please remove any personal identifiers (name, address, date of birth).*

*Thank you on behalf of the* ***I****nternational* ***T****askforce* ***F****or Depression* ***R****el****A****pse prevention (ITFRA)!*

|  | **VARIABLE** | **DESCRIPTION (VALUE LABEL)** | **AVAILABLE?** |
| --- | --- | --- | --- |
| 1 | Patient ID | Anonymised unique patient identifier |  |
| **DEMOGRAPHICS** | | | |
| 2 | Age | Patient age in years at baseline |  |
| 3 | Gender | Patient gender (0 = female, 1 = male, 2 = transgender, 3 = unknown) |  |
| 4 | Ethnicity | 0 = European; 1= indigenous; 2= Asian; 3 = African; 4 = American |  |
| 5 | Country of Birth | 0 = Current of study; 1=other |  |
| 6 | Education (highest level obtained) | 0 = no formal education; 1= education up to high school only; 2 = high school education; 3 = education after high school; 4 = post graduate education |  |
| 7 | Employment | 0 = employed full time; 1 = employed part-time; 2 = unemployed/beneficiary, 3 = student |  |
| 8 | Marital status | Married/cohabiting = 0, single = 1 |  |
| **INTERVENTION DETAILS** | | | |
| 9 | Group | Randomised group: |  |
| 10 | Number of sessions completed |  |  |
| 11 | Other adherence or compliance information | Data on whether the intervention was received as allocated, measures of use or measures of number of guidance sessions received |  |
| **PRIMARY OUTCOME (RELAPSE/RECURRENCE)** | | | |
| **FIRST FOLLOW-UP** | | | |
| 12 | First follow-up timepoint (days since completion of the intervention) |  |  |
| 13 | Follow up 1 depression diagnosis (primary) | Cumulative proportion of depressed participants (binary score) at first follow up on clinical interview |  |
| 14 | Follow up 1 depression diagnosis (primary) | Continuous variable indicating number of days till onset of depression (if available) |  |
| **SECOND FOLLOW-UP** | | | |
| 15 | Second follow-up timepoint (days since completion of the intervention) |  |  |
| 16 | Follow up 2 depression diagnosis (primary) | Cumulative proportion of depressed participants (binary score) at second follow up on clinical interview |  |
| 17 | Follow up 2 depression diagnosis (primary) | Continuous variable indicating number of days till onset of depression (if available) |  |
| **THIRD FOLLOW-UP** | | | |
| 18 | Third follow-up timepoint (days since completion of the intervention) |  |  |
| 19 | Follow up 3 depression diagnosis (primary) | Binary score at third follow up on clinical interview |  |
| 20 | Follow up 3 depression diagnosis (primary) | Cumulative proportion of depressed participants (binary score) at third follow up on clinical interview |  |
| ***FOURTH, FIFTH ETC. (ADD MORE FOLLOW-UPS IF NECESSARY)*** | | | |
| **SECONDARY OUTCOMES (DEPRESSION, ANXIETY, QUALITY OF LIFE)** | | | |
| **DEPRESSION** | | | |
| 21 | Follow up 1 depression (secondary) TOTAL SCORE | Continuous scores at first follow up on secondary depression instrument |  |
| 22 | Follow up 2 depression (secondary) TOTAL SCORE | Continuous scores on second follow up on secondary depression instrument |  |
| 23 | Follow up 3 depression (secondary) TOTAL SCORE | Continuous scores on third follow up on secondary depression instrument (if appropriate) |  |
| 24 | Last follow up for depression TOTAL SCORE | Continuous scores on last follow up on secondary depression instrument (if appropriate) |  |
| **ANXIETY** | | | |
| 25 | Follow up 1 anxiety (secondary) TOTAL SCORE | Continuous scores at first follow up on secondary anxiety instrument |  |
| 26 | Follow up 2 anxiety (secondary) TOTAL SCORE | Continuous scores on second follow up on secondary anxiety instrument |  |
| 27 | Follow up 3 anxiety (secondary) TOTAL SCORE | Continuous scores on third follow up on secondary anxiety instrument (if appropriate) |  |
| 28 | Last follow up for anxiety TOTAL SCORE | Continuous scores on last follow up on secondary anxiety instrument (if appropriate) |  |
| **QUALITY OF LIFE** | | | |
| 29 | Follow up 1 Quality Of life – QOLI score | Add data labels that are applicable to the specific measure used |  |
| 30 | Follow up 2 Quality Of life – QOLI score | Add data labels that are applicable to the specific measure used |  |
| 31 | Follow up 3 Quality Of life – QOLI score | Add data labels that are applicable to the specific measure used |  |
| 32 | Last follow up for QOLI score | Add data labels that are applicable to the specific measure used |  |
| **CLINICAL INDICATORS** | | | |
| **CLINICAL HISTORY** | | | |
| 33 | Number of previous depressive episodes |  |  |
| 34 | Age of onset of first episode of depression | In years |  |
| 35 | Time spent in remission since last episode | In days |  |
| 36 | Duration of past episode (in days) | In days |  |
| 37 | Stable or unstable remission (since last episode) | 0 = stable, 1 = unstable |  |
| 38 | History of childhood trauma | 0= yes, 1=no |  |
| **TREATMENT HISTORY** | | | |
| 40 | Previously received psychotherapy for MDD | 0= yes, 1=no |  |
| 41 | If yes, please add type of psychotherapy |  |  |
| 42 | If yes, please specify time since last therapy session at baseline. |  |  |
| 43 | Previously received medication for MDD | 0= yes, 1=no |  |
| 44 | If yes, specify type and dosage of ADM. |  |  |
| 45 | If yes, specify time since last dose at baseline. |  |  |
| **CLINICAL CHARACTERISTICS AT BASELINE** | | | |
| 46 | Co-morbid Anxiety Disorder | 0= yes, 1=no |  |
| 47 | Specify Anxiety Disorder |  |  |
| 48 | Co-morbid Mental Health (MH) disorder | 0= yes, 1=no |  |
| 49 | Specify co-morbid MH disorder |  |  |
| 50 | Co-morbid Physical Health (PH)disorder | 0= yes, 1=no |  |
| 51 | Specify co-morbid PH disorder |  |  |
| 52 | Receiving antidepressants for MDD at baseline | 0= yes, 1=no |  |
| 53 | If yes, specify type and dosage of ADM. |  |  |
| 54 | Baseline depression symptoms TOTAL SCORE | Continuous scores at baseline on depression instrument – |  |
| 55 | Quality Of life (provide as many subscale outcomes as measured)– QOLI score baseline | With data labels |  |
| 56 | Baseline anxiety TOTAL SCORE | Continuous scores at baseline on anxiety instrument – |  |

**** Please don’t forget the data key and codebook for included variables ****

*Note.* This supplementary material is an update to the variable collection sheet provided in Breedvelt et al. (2024).

# Supplementary Appendix 3. Overview of data wrangling (extracting, checking and recoding) upon receipt, per study.

We checked each individual dataset to assess whether the number of participants by arm corresponded with the primary reference. We further checked which variables were available in the paper provided and which were in the dataset. Where there were any discrepancies, we contacted the authors for the missing variables from their dataset. We further checked each individual trial dataset on basic demographic variables. The following variables were checked in their correspondence with the main dataset: No. participants, age, gender, education (highest level), no. previous depressive episodes. We furthermore checked the raw numbers of relapses reported for each paper against the datasets we were given. Any queries were resolved via email with the study authors. A few inconsistencies between the original papers and our findings were observed:

- **Biesheuvel-Leliefeld et al. (2017)**: number of previous episodes in the TAU group was slightly different in the dataset (49.2%) compared to reported in the paper (49.9%) potentially due to missing data. The standard deviation of age was reported 0.1 lower in the paper compared to the dataset.
- **Bockting et al. (2005)**: education level was reported as years of education in paper whereas only education level was provided in the dataset.
- **Bockting et al. (2018)**: The reported % relapses per arm differed slightly from the data reported in the dataset MADM = 62.3%, PCT/-ADM = 65.5%. Moreover, the reported HR was different in the paper (HR:1.07 (95%CI; 0.86 – 1.32 ) but as in the paper not significant. Upon consultation with the lead analyst this is probably due to differences in the recoding of missing data.
- **Bondolfi et al. (2010)**: no inconsistencies.
- **Farb et al. (2017)**: number of previous episodes in the CT group is slightly different in dataset (11/46 ≤ 3 previous episodes, 7/31 > 4 previous episodes) and paper (7/33 ≤ 3 previous episodes, 7/31 > 4 previous episodes), possibly due patients who dropped out during the study.
- **Hitchcock et al. (2013)**: Minor inconsistencies in age (data for one subject missing in received dataset) and number of relapses/time-till-relapse over 12 months, possibly due to the fact that missing data were imputed and as such reported in the primary reference.
- **Holländare et al. (2013)**: no inconsistencies except for a 0.1% difference in rounding of percentage per education level.
- **Hoorelbeeke et al. (2017)**: no inconsistencies.
- **Hoorelbeeke et al. (2019)**: no inconsistencies.
- **Huijbers et al. (2015)**: no inconsistencies.
- **Huijbers et al. (2016)**: number of previous episodes in the MBCT + discontinuation group is slightly different in dataset (6.1 episodes on average) as compared to the paper (5.9 episodes on average).
- **Jarrett et al. (2001)**: no inconsistencies.
- **Jarrett et al. (2013)**: no inconsistencies.
- **de Jonge et al. (2019)**: Age reported in paper (PCT = 42.1 years, CAU = 44.7 years) is slightly higher than age in dataset (PCT = 41.8, CAU = 44.3), possibly due to missing data. For education level, it is unclear how levels have been grouped to retrieve the data presented in the paper.
- **Klein et al. (2004)**: no inconsistencies.
- **Klein et al. (2018)**: no inconsistencies.
- **Kuyken et al. (2008)**: Number of previous episodes are slightly different in our dataset (M-ADM = 6.54, MBCT = 6.41) when compared to the data reported in the paper (M-ADM = 6.35, MBCT = 6.43).
- **Kuyken et al. (2015)**: no inconsistencies.
- **Moore et al. (2022)**: Only one minor inconsistency, as percentage females for the RGT-group is reported slightly different in manuscript Table 1 (57.9% instead of 59.7% as calculated using the received dataset, however this is regarded as a typographical error by the review team).
- **Segal et al. (2010)**: no inconsistencies.
- **Shallcross et al. (2018):** Percentage females in the MBCT group reported in the paper is incorrectly calculated (36/46*100% = 78% rather than 76%). This was regarded as calculation error, because the number of females reported in the paper corresponded to the number of females in the dataset. This has been clarified.

*Note*. This supplementary material is an update to the overview provided in Breedvelt et al. (2024).

# Supplementary Appendix 4. Side-by-side comparisons of RoB-1 and RoB-2 final judgements.

| **Study** | **Risk of Bias Version 1** | | | | | | | | | | | | **Risk of Bias Version 2** | | | | | |
| --- | --- | --- | --- | --- | --- | --- | --- | --- | --- | --- | --- | --- | --- | --- | --- | --- | --- | --- |
|  | *Sequence generation* | *Allocation concealment* | *Blinding of participants and personnel* | *Blinding of outcome assessors for outcomes* | *Incomplete outcome data* | *Selective outcome reporting* | *Co-interventions* | *Serious flaws* | *Intention-to-treat (ITT)* | *Similar groups* | *Compliance* | *Identical post timing* | *Randomization process* | *Deviations from intended interventions* | *Missing outcome data* | *Outcome measurement* | *Selection of reported results* | *Overall* |
| Bockting et al. (2005) | **+** | **+** | **-** | **+** | **+** | **+** | **+** | **+** | **-** | **+** | **+** | **+** | **+** | **+** | **+** | **+** | **+/-** | **+/-** |
| Bockting et al. (2018) | **+** | **+** | **-** | **+** | **+** | **+** | **+** | **+** | **+** | **+** | **+** | **+** | **+** | **+/-** | **+/-** | **+** | **+** | **-** |
| Bondolfi et al. (2010) | **+** | **+** | **-** | **+** | **+** | **+** | **-** | **?** | **+** | **+** | **+** | **?** | **+** | **+** | **+** | **+** | **+/-** | **+/-** |
| De Jonge et al. (2019) | **+** | **+** | **-** | **+** | **+** | **+** | **+** | **+** | **+** | **+** | **+** | **+** | **+** | **+/-** | **+** | **+** | **+** | **+/-** |
| Godfrin et al. (2010) | **?** | **+** | **-** | **+** | **+** | **+** | **?** | **?** | **+** | **+** | **?** | **+** | **+** | **+/-** | **+/-** | **-** | **+/-** | **-** |
| Hitchcock et al. (2021) | n/a | n/a | n/a | n/a | n/a | n/a | n/a | n/a | n/a | n/a | n/a | n/a | **+** | **+** | **+** | **+** | **+** | **+** |
| Holländare et al. (2013) | **-** | **?** | **-** | **+** | **+** | **?** | **+** | **+** | **+** | **-** | **?** | **+** | **+** | **+/-** | **+** | **-** | **+/-** | **-** |
| Huijbers et al. (2015) | **+** | **+** | **-** | **+** | **+** | **+** | **+** | **+** | **+** | **+** | **+** | **+** | **+** | **+/-** | **+/-** | **-** | **+** | **-** |
| Jarrett et al. (2001) | **+** | **+** | **-** | **+** | **+** | **+** | **+** | **+** | **+** | **+** | **?** | **+** | **+** | **+** | **+/-** | **+** | **+/-** | **-** |
| Jarrett et al. (2013) | **+** | **+** | **?** | **+** | **+** | **+** | **+** | **+** | **+** | **?** | **?** | **+** | **+** | **+** | **+** | **+** | **+** | **+** |
| Klein et al. (2004) | **?** | **?** | **-** | **+** | **?** | **+** | **?** | **+** | **-** | **-** | **?** | **+** | **+/-** | **+/-** | **+/-** | **+** | **+/-** | **-** |
| Klein et al. (2018) | **+** | **+** | **-** | **+** | **+** | **+** | **+** | **+** | **+** | **-** | **?** | **+** | **+** | **+/-** | **+/-** | **+** | **+** | **-** |
| Ma et al. (2004) | **+** | **-** | **-** | **+** | **+** | **+** | **+** | **+** | **+** | **+** | **+** | **+** | **+** | **+/-** | **+** | **+** | **+/-** | **-** |
| Moore et al. (2002) | n/a | n/a | n/a | n/a | n/a | n/a | n/a | n/a | n/a | n/a | n/a | n/a | **+/-** | **+/-** | **-** | **+** | **+/-** | **-** |
| Teasdale et al. (2000) | **?** | **?** | **-** | **+** | **+** | **+** | **+** | **+** | **+** | **-** | **+** | **?** | **+** | **+/-** | **+** | **+** | **+/-** | **-** |
| Williams et al. (2014) | **+** | **+** | **-** | **+** | **+** | **+** | **+** | **+** | **+** | **+** | **+** | **+** | **+** | **+** | **+** | **+** | **+** | **+** |
| *Abbreviations*. n/a = not applicable.  + = low  - = high  ? = unclear risk of bias (RoB-1 only)  +/- = some concerns (RoB-2 only) | | | | | | | | | | | | | | | | | | |

# Supplementary Appendix 5. Overview of risk of bias judgement and supportive descriptions, based on the RoB-2 tool

| **Study: Bockting et al. (2005)** | | |
| --- | --- | --- |
| *Domain 1: Randomization Process* | | |
| **Signaling Question** | **Response** | **Description** |
| 1.1. Was the allocation sequence random? | Y | “Randomization was performed using random permutated blocks and was stratified by study location and type of aftercare (family physician, psychiatric center, or no aftercare). Consecutively numbered, sealed envelopes contained computer-generated cards with concealed assignment codes. This procedure was organized and administered by an independent research associate.”  [Method, p648] |
| 1.2. Was the allocation sequence concealed until participants were enrolled and assigned to interventions? | Y |  |
| 1.3. Did baseline differences between intervention groups suggest a problem with the randomization process? | N | No baseline balances apparent or incompatible with chance, as seen in Table 1.  “Demographic and clinical characteristics of the intention-totreat group are summarized in Table 1. Both groups were comparable on each of the variables (all ps .10), except for number of previous episodes, 2(1, N 172) 4.43, p .04 (77/88 in the CT group had more than two previous episodes vs. 63/84 in the treatment as usual group)”  [Results, p650] |
| Authors risk-of-bias judgement of domain: | **LOW** | |
| *Domain 2: Deviations from intended interventions* | | |
| **Signaling Question** | **Response** | **Description** |
| 2.1. Were participants aware of their assigned intervention during the trial? | Y | Awareness of the assigned interventions is inherent to the design of the trial and the intervention examined, given that participants could be allocated to cognitive group therapy or treatment-as-usual. |
| 2.2. Were carers and people delivering the interventions aware of participants' assigned intervention during the trial? | Y |  |
| 2.3. If Y/PY/NI to 2.1 or 2.2: Were there deviations from the intended intervention that arose because of the trial context? | PN | “For the intention-to-treat analyses we excluded 15 patients (dropouts), 9 from the CT group because they did not attend any sessions and 6 from the treatment as usual group because they dropped out from the study immediately.”[Results, p650]  “Attrition, N=9 withdrew before start CT. Reasons: - Schedule problem (N=2), Withdrew from study (N=2) – Other (N=5). (N=6) withdrew in TAU group immediately after start study” [Figure 1, p650] |
| 2.4. If Y/PY to 2.3: Were these deviations likely to have affected the outcome? | n/a | n/a |
| 2.5. If Y/PY/NI to 2.4: Were these deviations from intended intervention balanced between groups? | n/a | n/a |
| 2.6. Was an appropriate analysis used to estimate the effect of assignment to intervention? | Y | “The analysis was performed with an intention-to-treat and a completers-analyses approach” [Statistical Analysis, p649] |
| 2.7. If N/PN/NI to 2.6: Was there potential for a substantial impact (on the result) of the failure to analyse participants in the group to which they were randomized? | n/a | n/a |
| Authors risk-of-bias judgement of domain: | **LOW** | |
| *Domain 3: Risk of bias due to missing outcome data* | | |
| **Signaling Question** | **Response** | **Description** |
| 3.1. Were data for this outcome available for all, or nearly all, participants randomized? | Y | Data at 12 months available for N = 172, out of n = 187 randomized to TAU or TAU+CT, which equals about 92% of the data. However, this is judged as adequate. |
| 3.2. If N/PN/NI to 3.1: Is there evidence that the result was not biased by missing outcome data? | n/a | n/a |
| 3.3. If N/PN to 3.2: Could missingness in the outcome depend on its true value? | n/a | n/a |
| 3.4. If Y/PY/NI to 3.3: Is it likely that missingness in the outcome depended on its true value? | n/a |  |
| Authors risk-of-bias judgement of domain: | **LOW** | |
| *Domain 4: Risk of bias in measurement of the outcome* | | |
| **Signaling Question** | **Response** | **Description** |
| 4.1. Was the method of measuring the outcome inappropriate? | N | “Relapse/recurrence. To assess relapse/recurrence, we used the Structured Clinical Interview for DSM–IV (SCID-I; First, Gibbon, Spitzer, & Williams, 1996). At baseline and at three follow-up assessments (3, 12, and 24 months), current and past depressive episodes were checked.”  [Study Measures, p640]  Using the SCID-I to judge relapse is considered suitable for this outcome, by the review team. |
| 4.2. Could measurement or ascertainment of the outcome have differed between intervention groups? | N | Relapse was measured and assessed at similar time-points and using the same methods in both groups.  “At baseline and at three follow-up assessments (3, 12, and 24 months), current and past depressive episodes were checked.” [Study Measures, p640] |
| 4.3. If N/PN/NI to 4.1 and 4.2: Were outcome assessors aware of the intervention received by study participants? | PN | “To maintain the assessors’ unawareness of treatment condition, we instructed participants not to reveal this information to the interviewers (psychologist/ research assistants). All interviews were audiotaped. Two independent, experienced psychiatrists who were blind to treatment condition evaluated all 108 occasions in which participants met DSM–IV criteria for major depression. In cases of disagreement, the ratings of the psychiatrists were used for further analyses.” [Study Measures, p640] |
| 4.4. If Y/PY/NI to 4.3: Could assessment of the outcome have been influenced by knowledge of intervention received? | n/a | n/a |
| 4.5. If Y/PY/NI to 4.4: Is it likely that assessment of the outcome was influenced by knowledge of intervention received? | n/a |  |
| Authors risk-of-bias judgement of domain: | **LOW** | |
| *Domain 5: Risk of bias in selection of the reported result* | | |
| **Signaling Question** | **Response** | **Description** |
| 5.1. Were the data that produced this result analysed in accordance with a pre-specified analysis plan that was finalized before unblinded outcome data were available for analysis? | NI | No detailed pre-specified intentions available to the review authors. Thus, planned outcome measures and analyses cannot be compared to those presented in the reviewed trials/articles. |
| Is the numerical result being assessed likely to have been selected, on the basis of the results, from... | | |
| 5.2. ...multiple eligible outcome measurements (e.g. scales, definitions, time points) within the outcome domain? | PN | As measurement of the event ‘relapse’ can only be done in one specific way according to the SCID-I, there is no opportunity to select from multiple measures. Additionally, based on the reports across multiple articles - including those reporting on follow-up data - reviewers are fairly confident that all the eligible results are reported according with the intended measurements. |
| 5.3. ...multiple eligible analyses of the data? | NI | Analysis intentions are not available or reported in sufficient detail to facilitate the assessment and reach a valid judgement. |
| Authors risk-of-bias judgement of domain: | **SOME CONCERNS** | |
| **Study: Bockting et al. (2018)** | | |
| *Domain 1: Randomization Process* | | |
| **Signaling Question** | **Response** | **Description** |
| 1.1. Was the allocation sequence random? | Y | “An independent research assistant masked to the randomisation sequence entered the stratification characteristics and implemented the automated permuted-block randomisation using computer-generated random numbers with a predefined allocation ratio of 10:10:8 to PCT and antidepressants, antidepressants alone, and PCT while tapering off antidepressants. Randomisation was stratified for number of previous major depressive episodes (two vs three or more) and type of care (GP vs secondary mental health care).” [Methods, p403] |
| 1.2. Was the allocation sequence concealed until participants were enrolled and assigned to interventions? | Y |  |
| 1.3. Did baseline differences between intervention groups suggest a problem with the randomization process? | N | No imbalances are apparent and observed imbalances are compatible with chance, as can be gathered from the manuscript and the unnumbered table on p407. |
| Authors risk-of-bias judgement of domain: | **LOW** | |
| *Domain 2: Deviations from intended interventions* | | |
| **Signaling Question** | **Response** | **Description** |
| 2.1. Were participants aware of their assigned intervention during the trial? | Y | Awareness of the assigned interventions is inherent to the design of the trial and the intervention examined, given that participants could be allocated to PCT and maintenance ADM, PCT with tapering, or maintenance ADM. |
| 2.2. Were carers and people delivering the interventions aware of participants' assigned intervention during the trial? | Y |  |
| 2.3. If Y/PY/NI to 2.1 or 2.2: Were there deviations from the intended intervention that arose because of the trial context? | PY | “In 43 (23%) of 189 participants assigned to PCT, the intervention was not started and follow-up data were not available because the travel time was too long to visit the PCT group or the group sessions were planned at a time they could not attend. Therefore, from February, 2013, we decided to offer individual sessions as well as group sessions. In addition to these 43 participants, 16 in the PCT groups and 21 in the antidepressants alone group dropped out for other reasons.”  [Results, p405] |
| 2.4. If Y/PY to 2.3: Were these deviations likely to have affected the outcome? | PN | “More than half of participants still in the study after 6 months adhered to the antidepressant protocol (30 [60%] of 50 participants in the PCT with tapering of antidepressants group, 36 [65%] of 55 in the combined treatment group, and 38 [58%] of 66 in the antidepressants alone group; appendix)” [Results, p407] |
| 2.5. If Y/PY/NI to 2.4: Were these deviations from intended intervention balanced between groups? | n/a | n/a |
| 2.6. Was an appropriate analysis used to estimate the effect of assignment to intervention? | Y | “Primary analyses were done by intention to treat.”[Statistical analysis, p404] |
| 2.7. If N/PN/NI to 2.6: Was there potential for a substantial impact (on the result) of the failure to analyse participants in the group to which they were randomized? | n/a | n/a |
| Authors risk-of-bias judgement of domain: | **SOME CONCERNS** | |
| *Domain 3: Risk of bias due to missing outcome data* | | |
| **Signaling Question** | **Response** | **Description** |
| 3.1. Were data for this outcome available for all, or nearly all, participants randomized? | PN | In total, 289 participants were randomly assigned. Of these, 62+55+72 completed the 15-month follow-up. This equates 65.4% and can thus be seen as insufficient. |
| 3.2. If N/PN/NI to 3.1: Is there evidence that the result was not biased by missing outcome data? | PN | Multiple imputation was used, but imputation of outcome variables should not be assumed to correct for any bias. |
| 3.3. If N/PN to 3.2: Could missingness in the outcome depend on its true value? | PY | It is possible that dropout, while comparable across groups, is related to participants health status and depression severity. It could be that missingness was influenced by its true value. However, considering results from use of imputation and sensitivity analysis, it is unlikely that missingness did depend on its true value. |
| 3.4. If Y/PY/NI to 3.3: Is it likely that missingness in the outcome depended on its true value? | PN |  |
| Authors risk-of-bias judgement of domain: | **SOME CONCERNS** | |
| *Domain 4: Risk of bias in measurement of the outcome* | | |
| **Signaling Question** | **Response** | **Description** |
| 4.1. Was the method of measuring the outcome inappropriate? | N | “Outcomes The primary outcome was time-related proportion of participants with recurrence over a follow-up of 24 months, analysed in terms of time to recurrence. Participants were assessed for remission by trained assessors at baseline and after 3, 9, 15, and 24 months using DSM-IV-TR criteria assessed with SCID-I, including retrospective parts and information from monthly ratings on the Inventory of Depressive Symptomatology–Self Report (IDS-SR).29 Four trained interviewers (NSK, CS, and two research assistants) rated a subset of 50 interviews, resulting in an inter-rater agreement of 0·96, indicating excellent agreement.” [Outcomes, p404]  Using the SCID-I to judge relapse is considered suitable for this outcome, by the review team. |
| 4.2. Could measurement or ascertainment of the outcome have differed between intervention groups? | PN | “…were assessed for remission by trained assessors at baseline and after 3, 9, 15, and 24 months…” [Outcomes, p404] |
| 4.3. If N/PN/NI to 4.1 and 4.2: Were outcome assessors aware of the intervention received by study participants? | N | No, as it is a single-blind study and outcome assessors were trained assessors and interviewers. |
| 4.4. If Y/PY/NI to 4.3: Could assessment of the outcome have been influenced by knowledge of intervention received? | n/a | n/a |
| 4.5. If Y/PY/NI to 4.4: Is it likely that assessment of the outcome was influenced by knowledge of intervention received? | n/a |  |
| Authors risk-of-bias judgement of domain: | **LOW** | |
| *Domain 5: Risk of bias in selection of the reported result* | | |
| **Signaling Question** | **Response** | **Description** |
| 5.1. Were the data that produced this result analysed in accordance with a pre-specified analysis plan that was finalized before unblinded outcome data were available for analysis? | Y | Trial protocol is available, and data produced is in accordance to this analysis plan. See Bockting et al. (2011). Disrupting the rhythm of depression: design and protocol of a randomized controlled trial on preventing relapse using brief cognitive therapy with or without antidepressants. BMC psychiatry, 11, 1-9. |
| Is the numerical result being assessed likely to have been selected, on the basis of the results, from... | | |
| 5.2. ...multiple eligible outcome measurements (e.g. scales, definitions, time points) within the outcome domain? | PN | All eligible reported results for the outcome measurement seem to correspond to all intended measurement. |
| 5.3. ...multiple eligible analyses of the data? | PN | All eligible reported results for the outcome measurement seem to correspond to all intended analyses. Additionally, analyses are consistent across several different reports on this trial. |
| Authors risk-of-bias judgement of domain: | **LOW** | |
| **Study: Bondolfi et al. (2010)** | | |
| *Domain 1: Randomization Process* | | |
| **Signaling Question** | **Response** | **Description** |
| 1.1. Was the allocation sequence random? | Y | “Randomization was performed by the last author (GBe) independently from the research team. A stratified block randomization procedure was implemented. … After checking for inclusion and exclusion criteria and informed consent had been obtained, intervention was assigned to patients through sealed envelopes. Patients were instructed not to inform the research team about group assignment to insure that blind outcome assessment could be performed”  [Methods, p226] |
| 1.2. Was the allocation sequence concealed until participants were enrolled and assigned to interventions? | Y |  |
| 1.3. Did baseline differences between intervention groups suggest a problem with the randomization process? | N | No baseline imbalances apparent to reviewers.  “The TAU and MBCT + TAU groups did not differ statistically with respect to socio-demographic variables (age, gender, and education) or clinical history (age at onset of depression, number of past episodes, and number of hospitalizations). Baseline MADRS did not differ between the groups.”  [Results, p227] |
| Authors risk-of-bias judgement of domain: | **LOW** | |
| *Domain 2: Deviations from intended interventions* | | |
| **Signaling Question** | **Response** | **Description** |
| 2.1. Were participants aware of their assigned intervention during the trial? | Y | Awareness of the assigned interventions is inherent to the design of the trial and the intervention examined, given that participants could be allocated to MBCT+TAU or TAU only. |
| 2.2. Were carers and people delivering the interventions aware of participants' assigned intervention during the trial? | Y |  |
| 2.3. If Y/PY/NI to 2.1 or 2.2: Were there deviations from the intended intervention that arose because of the trial context? | PN | “The intent to treat sample (ITT) consisted of 60 patients who were randomized to either TAU (n = 29) or MBCT + TAU (n = 31) group. Regarding the MBCT + TAU group, the average number of MBCT sessions completed was 7.00 ± 1.09 and 90% (28/31) of the MBCT participants completed the 2-month program. Complete data on 14-month relapse or recurrence were available for 55 patients (per-protocol sample, PP).” [Patients flow, p227]  “There was also no difference between groups regarding the use of antidepressant and non-pharmacological treatment during the study. Antidepressant medication was reinstated in 36% of the MBCT + TAU participants and in 31% of the TAU participants (Fisher's exact test, p = .78). Delay until onset of antidepressant medication was similar in both groups (median in days (min–max); MBCT + TAU: 204 (19–437); TAU: 111 (30–385); Mann–Whitney U-test, p = .40). One or more visit for counselling, psychotherapy or support from professional mental health staff was undertaken for 46% of the MBCT + TAU group and for 55% of the TAU group (Fisher's exact test, p = .60). One or more visit to general practitioner were undertaken for 29% of the MBCT + TAU group and for 24% of the TAU group (Fisher's exact test, p = .77).”  [Patient characteristics, p227] |
| 2.4. If Y/PY to 2.3: Were these deviations likely to have affected the outcome? | n/a | n/a |
| 2.5. If Y/PY/NI to 2.4: Were these deviations from intended intervention balanced between groups? | n/a | n/a |
| 2.6. Was an appropriate analysis used to estimate the effect of assignment to intervention? | Y | Analyses were conducted for both ITT and PP samples. |
| 2.7. If N/PN/NI to 2.6: Was there potential for a substantial impact (on the result) of the failure to analyse participants in the group to which they were randomized? | n/a | n/a |
| Authors risk-of-bias judgement of domain: | **LOW** | |
| *Domain 3: Risk of bias due to missing outcome data* | | |
| **Signaling Question** | **Response** | **Description** |
| 3.1. Were data for this outcome available for all, or nearly all, participants randomized? | Y | 60 participants were randomized in total. Of these, 55 completed the final follow-up and outcome data is thus available for most participants (±91%). |
| 3.2. If N/PN/NI to 3.1: Is there evidence that the result was not biased by missing outcome data? | n/a | n/a |
| 3.3. If N/PN to 3.2: Could missingness in the outcome depend on its true value? | n/a | n/a |
| 3.4. If Y/PY/NI to 3.3: Is it likely that missingness in the outcome depended on its true value? | n/a |  |
| Authors risk-of-bias judgement of domain: | **LOW** | |
| *Domain 4: Risk of bias in measurement of the outcome* | | |
| **Signaling Question** | **Response** | **Description** |
| 4.1. Was the method of measuring the outcome inappropriate? | N | “Our primary dependent measure, the occurrence of relapse or recurrence meeting DSM-IV criteria for major depressive episode, was assessed with the depression module of the Structured Clinical Interview for DSM-IV (First et al., 1996) by raters blind to group assignment (FJ, PhD psychologist, 2nd author and CG clinical research nurse, 8th author). An experienced clinical psychologist with formal training in the use of the SCID trained the two research staff. To ensure accuracy, all the assessments were audiotaped. A senior psychiatrist, blind to group assignment, then rated all actual, borderline or probable relapse/recurrences. When disagreement occurred between the interviewer and the senior psychiatrist's assessments, consensus was reached through discussion.” [2.4 Measures, p226]  Using the SCID-I to judge relapse is considered suitable for this outcome, by the review team |
| 4.2. Could measurement or ascertainment of the outcome have differed between intervention groups? | PN | “Patients were interviewed at baseline, 2 months later (end of therapy sessions for participants allocated to MBCT + TAU) and at 3month intervals during follow-up (months 5, 8, 11 and 14).” [2.4 Measures, p226-227] |
| 4.3. If N/PN/NI to 4.1 and 4.2: Were outcome assessors aware of the intervention received by study participants? | PN | “…by raters blind to group assignment…” [2.4 Measures, p226] |
| 4.4. If Y/PY/NI to 4.3: Could assessment of the outcome have been influenced by knowledge of intervention received? | n/a | n/a |
| 4.5. If Y/PY/NI to 4.4: Is it likely that assessment of the outcome was influenced by knowledge of intervention received? | n/a |  |
| Authors risk-of-bias judgement of domain: | **LOW** | |
| *Domain 5: Risk of bias in selection of the reported result* | | |
| **Signaling Question** | **Response** | **Description** |
| 5.1. Were the data that produced this result analysed in accordance with a pre-specified analysis plan that was finalized before unblinded outcome data were available for analysis? | NI | No detailed pre-specified intentions available to the review authors. Thus, planned outcome measures and analyses cannot be compared to those presented in the reviewed trials/articles. |
| Is the numerical result being assessed likely to have been selected, on the basis of the results, from... | | |
| 5.2. ...multiple eligible outcome measurements (e.g. scales, definitions, time points) within the outcome domain? | NI | Pre-specified definitions of outcomes are not available or reported in sufficient detail to facilitate the assessment and reach a valid judgement. |
| 5.3. ...multiple eligible analyses of the data? | NI | Analysis intentions are not available or reported in sufficient detail to facilitate the assessment and reach a valid judgement. |
| Authors risk-of-bias judgement of domain: | **SOME CONCERNS** | |
| **Study: Godfrin et al. (2010)** | | |
| *Domain 1: Randomization Process* | | |
| **Signaling Question** | **Response** | **Description** |
| 1.1. Was the allocation sequence random? | Y | “…using a computer-generated randomization procedure. The sequence of allocation to the study groups was concealed until assignment.”  [Method, p739] |
| 1.2. Was the allocation sequence concealed until participants were enrolled and assigned to interventions? | Y |  |
| 1.3. Did baseline differences between intervention groups suggest a problem with the randomization process? | N | “No significant group differences were found.”  [Results, p741]  No imbalances are apparent and observed imbalances are compatible with chance. |
| Authors risk-of-bias judgement of domain: | **LOW** | |
| *Domain 2: Deviations from intended interventions* | | |
| **Signaling Question** | **Response** | **Description** |
| 2.1. Were participants aware of their assigned intervention during the trial? | Y | Awareness of the assigned interventions is inherent to the design of the trial and the intervention examined, given that participants could be allocated to MBT+TAU or TAU only. |
| 2.2. Were carers and people delivering the interventions aware of participants' assigned intervention during the trial? | Y |  |
| 2.3. If Y/PY/NI to 2.1 or 2.2: Were there deviations from the intended intervention that arose because of the trial context? | NI | “Non-trial treatment A comparative analysis revealed differences between study groups in terms of non-trial treatment. At 8-month-follow-up, patients in the MBCT plus TAU group reported less visits to their general practitioner with depression-related complaints than patients in the TAU group. At baseline and at 14-month follow-up, patients in the MBCT plus TAU group reported more commonly visits to a psychiatrist than patients in the TAU group. However, no differences in frequency of antidepressant medication or psychological treatment emerged at any stage of the study period. Seen from a longitudinal perspective, analysis of non-trial treatment at 8 and 14 months showed a decreased frequency of antidepressant medication in the MBCT plus TAU group (Fisher’s exact test = 11.63, p < 0.01) but not in the TAU group (Table 2).” [Results, p741]  Nevertheless, the above does constitute trial protocol interventions as participants in both groups had access to TAU. There is not enough further information available to make an adequate judgement. |
| 2.4. If Y/PY to 2.3: Were these deviations likely to have affected the outcome? | n/a | n/a |
| 2.5. If Y/PY/NI to 2.4: Were these deviations from intended intervention balanced between groups? | n/a | n/a |
| 2.6. Was an appropriate analysis used to estimate the effect of assignment to intervention? | Y | “…the statistical analyses are based on the intent-to-treat sample.”[Results, p741] |
| 2.7. If N/PN/NI to 2.6: Was there potential for a substantial impact (on the result) of the failure to analyse participants in the group to which they were randomized? | n/a | n/a |
| Authors risk-of-bias judgement of domain: | **SOME CONCERNS** | |
| *Domain 3: Risk of bias due to missing outcome data* | | |
| **Signaling Question** | **Response** | **Description** |
| 3.1. Were data for this outcome available for all, or nearly all, participants randomized? | PN | A total of 106 participants were randomized to either one of the two groups. From these, 18+12 were lost-to-follow-up. From this it follows that 71.7% of the follow-up data is available and this is judged by reviewers as inadequate. |
| 3.2. If N/PN/NI to 3.1: Is there evidence that the result was not biased by missing outcome data? | PN | “Over the total study period, no significant group differences in lost-to-follow-up-rates emerged (MBCT plus TAU: N = 18/52 [34.6%]; TAU: N = 12/54 [22.2%]; x2 = 2.01, df = 1, p > 0.05” [Recruitment and retention, p740].  No evidence that results were not biased, as no bias correcting analysis methods or adequate sensitivity analyses are apparent to the review team. |
| 3.3. If N/PN to 3.2: Could missingness in the outcome depend on its true value? | PY | It is possible that dropout, while somewhat comparable across groups, is related to participants depression status and it could thus be that missingness was influenced by its true value. However, this is not judged as likely by the reviewers. |
| 3.4. If Y/PY/NI to 3.3: Is it likely that missingness in the outcome depended on its true value? | PN |  |
| Authors risk-of-bias judgement of domain: | **SOME CONCERNS** | |
| *Domain 4: Risk of bias in measurement of the outcome* | | |
| **Signaling Question** | **Response** | **Description** |
| 4.1. Was the method of measuring the outcome inappropriate? | N | “The Structured Clinical Interview for DSM-IV Axis-I disorders (SCID-I) (Van Groenestijn, Akkerhuis, Kupka, Schneider, & Nolen, 1999) is a structured clinical interview and aims at diagnosing axis-I psychiatric disorders, based on the DSM-IV.”  [Assessment, p739]  Using the SCID-I to judge relapse is considered suitable for this outcome, by the review team. |
| 4.2. Could measurement or ascertainment of the outcome have differed between intervention groups? | PN | “All patients were assessed by a skilled clinical psychologist by means of structured interviews and self-report measures, filled in by the patients, at baseline and 2 (i.e. after completion of the training in MBCT-allocated patients), 8 and 14 months afterwards.”  [Assessment, p739] |
| 4.3. If N/PN/NI to 4.1 and 4.2: Were outcome assessors aware of the intervention received by study participants? | NI | Not enough information available to come to a judgement. |
| 4.4. If Y/PY/NI to 4.3: Could assessment of the outcome have been influenced by knowledge of intervention received? | Y | Yes, it is possible that knowledge of treatment allocation could have influenced the assessment, as the clinical psychologist was required to come to a judgement (vs. a more objective outcome such as all-cause mortality).  However, there is not enough information available to believe that undue influence was likely. That is, there is no strong level of belief that outcomes were influenced and not enough information available. NB. If bias was introduced, it is likely to be in favor of the intervention group. |
| 4.5. If Y/PY/NI to 4.4: Is it likely that assessment of the outcome was influenced by knowledge of intervention received? | NI |  |
| Authors risk-of-bias judgement of domain: | **HIGH** | |
| *Domain 5: Risk of bias in selection of the reported result* | | |
| **Signaling Question** | **Response** | **Description** |
| 5.1. Were the data that produced this result analysed in accordance with a pre-specified analysis plan that was finalized before unblinded outcome data were available for analysis? | NI | A pre-specified is available (see <https://clinicaltrials.gov/study/NCT00259506#study-plan>), which was first pre-registered on 2005-11-28. However, this is insufficiently detailed to compare planned outcome measures and analyses to those presented in the reviewed trials/articles. |
| Is the numerical result being assessed likely to have been selected, on the basis of the results, from... | | |
| 5.2. ...multiple eligible outcome measurements (e.g. scales, definitions, time points) within the outcome domain? | NI | Pre-specified definitions of outcomes are not available or reported in sufficient detail to facilitate the assessment and reach a valid judgement. |
| 5.3. ...multiple eligible analyses of the data? | NI | Analysis intentions are not available or reported in sufficient detail to facilitate the assessment and reach a valid judgement. |
| Authors risk-of-bias judgement of domain: | **SOME CONCERNS** | |
| **Study: Hitchcock et al. (2021)** | | |
| *Domain 1: Randomization Process* | | |
| **Signaling Question** | **Response** | **Description** |
| 1.1. Was the allocation sequence random? | Y | “Participants were randomized to condition by the trial statistician (Watson) using a computer-generated random number allocation, and initial session facilitators were informed of treatment allocation after completion of the baseline assessment by opening a sealed opaque envelope.”  [1.6 Procedure, p5] |
| 1.2. Was the allocation sequence concealed until participants were enrolled and assigned to interventions? | Y |  |
| 1.3. Did baseline differences between intervention groups suggest a problem with the randomization process? | N | Two treatment groups are of comparable sizes. Baseline characteristics are similar in both groups as follows from Table 1. No imbalances apparent between groups or incompatible with chance. |
| Authors risk-of-bias judgement of domain: | **LOW** | |
| *Domain 2: Deviations from intended interventions* | | |
| **Signaling Question** | **Response** | **Description** |
| 2.1. Were participants aware of their assigned intervention during the trial? | Y | Awareness of the assigned interventions is inherent to the design of the trial and the intervention examined, given that participants could be allocated to MemFlex or psychoeducation. |
| 2.2. Were carers and people delivering the interventions aware of participants' assigned intervention during the trial? | Y |  |
| 2.3. If Y/PY/NI to 2.1 or 2.2: Were there deviations from the intended intervention that arose because of the trial context? | PN | “We achieved good adherence for a self-guided intervention, with 83.8% of MemFlex participants and 91.9% of Psychoeducation participants completing all eight workbook sessions. Non-completers were those who did not attend the post assessment, and as such, we were unable to record how many workbook sessions they had completed” [Results, p5]  “No significant between-group differences were observed for demographic or concurrent treatment variables (see Table 1).” [Results, p5] |
| 2.4. If Y/PY to 2.3: Were these deviations likely to have affected the outcome? | n/a | n/a |
| 2.5. If Y/PY/NI to 2.4: Were these deviations from intended intervention balanced between groups? | n/a | n/a |
| 2.6. Was an appropriate analysis used to estimate the effect of assignment to intervention? |  | “We report intent-to-treat analyses (and thus intent-to-treat means) for our primary and secondary outcomes using multiple imputation for missing data, applying Satterthwaite’s correction.” [Method, p5] |
| 2.7. If N/PN/NI to 2.6: Was there potential for a substantial impact (on the result) of the failure to analyse participants in the group to which they were randomized? | n/a | n/a |
| Authors risk-of-bias judgement of domain: | **LOW** | |
| *Domain 3: Risk of bias due to missing outcome data* | | |
| **Signaling Question** | **Response** | **Description** |
| 3.1. Were data for this outcome available for all, or nearly all, participants randomized? | PY | In total, 74 participants were randomized (*n* = 37 in the MemFlex condition and *n* = 37 in the Psychoeducation condition). At 12 months, outcome data was available for n = 29 and n = 29 participants, respectively, which totals at 78.38%. |
| 3.2. If N/PN/NI to 3.1: Is there evidence that the result was not biased by missing outcome data? | n/a | n/a |
| 3.3. If N/PN to 3.2: Could missingness in the outcome depend on its true value? | n/a | n/a |
| 3.4. If Y/PY/NI to 3.3: Is it likely that missingness in the outcome depended on its true value? | n/a |  |
| Authors risk-of-bias judgement of domain: | **LOW** | |
| *Domain 4: Risk of bias in measurement of the outcome* | | |
| **Signaling Question** | **Response** | **Description** |
| 4.1. Was the method of measuring the outcome inappropriate? | N | “Primary clinical outcomes. The co-primary clinical outcomes were number of depression free days from post-intervention to twelve-month follow-up, and time until depressive relapse until 12 month follow-up, both measured using the Longitudinal Interval Follow-up Evaluation (LIFE) for the SCID (Keller et al., 1987). All LIFE assessments were reviewed by the supervising clinical psychologist who was blind to intervention allocation. In the case of disagreement with the original assessor, the clinical psychologist re-administered the LIFE. One hundred percent agreement was obtained between raters for the final data.” [Method, p4]  Using the SCID-I to judge relapse is considered suitable for this outcome, by the review team. |
| 4.2. Could measurement or ascertainment of the outcome have differed between intervention groups? | N | “Assessments were completed at baseline, post-intervention, six month follow-up, and at twelve month follow-up (the primary clinical endpoint).” [Method, p2] |
| 4.3. If N/PN/NI to 4.1 and 4.2: Were outcome assessors aware of the intervention received by study participants? | PN | “All LIFE assessments were reviewed by the supervising clinical psychologist who was blind to intervention allocation. In the case of disagreement with the original assessor, the clinical psychologist re-administered the LIFE. One hundred percent agreement was obtained between raters for the final data.” [Method, p4] |
| 4.4. If Y/PY/NI to 4.3: Could assessment of the outcome have been influenced by knowledge of intervention received? | n/a | n/a |
| 4.5. If Y/PY/NI to 4.4: Is it likely that assessment of the outcome was influenced by knowledge of intervention received? | n/a |  |
| Authors risk-of-bias judgement of domain: | **LOW** | |
| *Domain 5: Risk of bias in selection of the reported result* | | |
| **Signaling Question** | **Response** | **Description** |
| 5.1. Were the data that produced this result analysed in accordance with a pre-specified analysis plan that was finalized before unblinded outcome data were available for analysis? | Y | A pre-registration for the trial (NCT02614326) on clinicialtrials.gov and a trial protocol are available and were reviewed.  https://clinicaltrials.gov/study/NCT02614326?term=NCT 02614326&rank=1  Hitchcock, C., Gormley, S., O’Leary, C., Rodrigues, E., Wright, I., Griffiths, K., ... Dalgleish, T. (2018). Study protocol for a randomised, controlled platform trial estimating the effect of autobiographical Memory Flexibility training (MemFlex) on relapse of recurrent major depressive disorder. BMJ Open, 8(1). https://doi.org/ 10.1136/bmjopen-2017-018194.  Researchers did indeed pre-specify their intentions in sufficient detail, and they match the published manuscript. |
| Is the numerical result being assessed likely to have been selected, on the basis of the results, from... | | |
| 5.2. ...multiple eligible outcome measurements (e.g. scales, definitions, time points) within the outcome domain? | PN | All eligible reported results for the outcome measurement seem to correspond to all intended measurements. |
| 5.3. ...multiple eligible analyses of the data? | PN | All eligible reported results for the outcome measurement seem to correspond to all intended analyses. |
| Authors risk-of-bias judgement of domain: | **LOW** | |
| **Study: Holländare et al. (2011)** | | |
| *Domain 1: Randomization Process* | | |
| **Signaling Question** | **Response** | **Description** |
| 1.1. Was the allocation sequence random? | Y | “...were then randomized to Internet-based CBT or a control group by drawing numbers (each corresponding to a person) out of an opaque bowl.”  [Materials and methods, p287]  No information available/reported regarding allocation sequence concealment, but no reason to suspect that enrolling investigators or participants had knowledge of forthcoming allocations. |
| 1.2. Was the allocation sequence concealed until participants were enrolled and assigned to interventions? | PY |  |
| 1.3. Did baseline differences between intervention groups suggest a problem with the randomization process? | N | Two treatment groups are of comparable sizes. Baseline characteristics are similar in both groups as follows from Table 1. No imbalances apparent between groups or incompatible with chance. |
| Authors risk-of-bias judgement of domain: | **LOW** | |
| *Domain 2: Deviations from intended interventions* | | |
| **Signaling Question** | **Response** | **Description** |
| 2.1. Were participants aware of their assigned intervention during the trial? | Y | Awareness of the assigned interventions is inherent to the design of the trial and the intervention examined, given that participants could be allocated to online CBT or the control condition (consisting of low intensity contact only).  “Over a 10-week period, participants in the treatment group worked through CBT modules with guidance (via e-mail) from a personal therapist, after which no guidance was received; however, they were offered the remaining modules.”  [Materials and methods, p287]  “Control group participants had the possibility of e-mail contact with a personal therapist; however, the content of the correspondence was restricted to non-specific support.”  [Materials and methods, p287] |
| 2.2. Were carers and people delivering the interventions aware of participants' assigned intervention during the trial? | Y |  |
| 2.3. If Y/PY/NI to 2.1 or 2.2: Were there deviations from the intended intervention that arose because of the trial context? | NI | No comments in the papers found by reviewers on whether deviations arose because of the trial’s context. |
| 2.4. If Y/PY to 2.3: Were these deviations likely to have affected the outcome? | n/a | n/a |
| 2.5. If Y/PY/NI to 2.4: Were these deviations from intended intervention balanced between groups? | n/a | n/a |
| 2.6. Was an appropriate analysis used to estimate the effect of assignment to intervention? | PY | Not specified explicitly in the published manuscript, but appears that analyses were conducted based on the ITT principle. |
| 2.7. If N/PN/NI to 2.6: Was there potential for a substantial impact (on the result) of the failure to analyse participants in the group to which they were randomized? | n/a | n/a |
| Authors risk-of-bias judgement of domain: | **SOME CONCERNS** | |
| *Domain 3: Risk of bias due to missing outcome data* | | |
| **Signaling Question** | **Response** | **Description** |
| 3.1. Were data for this outcome available for all, or nearly all, participants randomized? | PY | “We obtained complete data on relapse from 67 (79.8%) of the 84 participants initially included in this two-year follow-up; 32/42 in the iCBT group and 35/42 in the control group. The remaining 17 contributed with data covering varying lengths of time until they dropped out”  [Results, p720] |
| 3.2. If N/PN/NI to 3.1: Is there evidence that the result was not biased by missing outcome data? | n/a | n/a |
| 3.3. If N/PN to 3.2: Could missingness in the outcome depend on its true value? | n/a | n/a |
| 3.4. If Y/PY/NI to 3.3: Is it likely that missingness in the outcome depended on its true value? | n/a |  |
| Authors risk-of-bias judgement of domain: | **LOW** | |
| *Domain 4: Risk of bias in measurement of the outcome* | | |
| **Signaling Question** | **Response** | **Description** |
| 4.1. Was the method of measuring the outcome inappropriate? | PN | The SCID-I, MADRS and BDI were used as main outcome measures, which have been shown to have adequate psychometric qualities.  Using the SCID-I to judge relapse is considered suitable for this outcome, by the review team. |
| 4.2. Could measurement or ascertainment of the outcome have differed between intervention groups? | PN | “All participants, irrespective of their MADRS-S score, were interviewed at post treatment as well as after 6, 12, and 24 months.”  [Material and methods, p720] |
| 4.3. If N/PN/NI to 4.1 and 4.2: Were outcome assessors aware of the intervention received by study participants? | Y | Checked with author: SCID assessors were not blind to treatment allocation, so they were aware of interventions received by participants. |
| 4.4. If Y/PY/NI to 4.3: Could assessment of the outcome have been influenced by knowledge of intervention received? | PY | This is possible, as relapse is an observer-reported outcome involving clinical judgement.  However, there is not enough information available to believe that undue influence was likely. That is, there is no strong level of belief that outcomes were influenced and not enough information available. NB. If bias was introduced, it is likely to be in favor of the intervention group. |
| 4.5. If Y/PY/NI to 4.4: Is it likely that assessment of the outcome was influenced by knowledge of intervention received? | NI |  |
| Authors risk-of-bias judgement of domain: | **HIGH** | |
| *Domain 5: Risk of bias in selection of the reported result* | | |
| **Signaling Question** | **Response** | **Description** |
| 5.1. Were the data that produced this result analysed in accordance with a pre-specified analysis plan that was finalized before unblinded outcome data were available for analysis? | NI | No detailed pre-specified intentions available to the review authors. Thus, planned outcome measures and analyses cannot be compared to those presented in the reviewed trials/articles. |
| Is the numerical result being assessed likely to have been selected, on the basis of the results, from... | | |
| 5.2. ...multiple eligible outcome measurements (e.g. scales, definitions, time points) within the outcome domain? | PN | Based on the reports across multiple articles - including those reporting on follow-up data - reviewers are fairly confident that all eligible results are reported. All eligible reported results for the outcome measurement seem correspond to all intended measurement. |
| 5.3. ...multiple eligible analyses of the data? | NI | Analysis intentions are not available or reported in sufficient detail to facilitate the assessment and reach a valid judgement. |
| Authors risk-of-bias judgement of domain: | **SOME CONCERNS** | |
| **Study: Huijbers et al. (2015)** | | |
| *Domain 1: Randomization Process* | | |
| **Signaling Question** | **Response** | **Description** |
| 1.1. Was the allocation sequence random? | Y | “Randomisation was performed using a website-based application, developed specifically for this study by an independent statistician, with a minimisation procedure for research centre, full versus partial remission”  “Randomisation took place after the clinical interview and participants were informed about the assigned treatment immediately by the research assistant”  [Methods, p55]  From the study protocol, Huijbers et al. 2012: “Randomization is performed online by the research assistant who conducts the baseline assessment by entering the required information on a randomization website specifically designed for this study.” [Randomization, p7] |
| 1.2. Was the allocation sequence concealed until participants were enrolled and assigned to interventions? | Y |  |
| 1.3. Did baseline differences between intervention groups suggest a problem with the randomization process? | N | No imbalances are apparent and observed imbalances are compatible with chance, as can be gathered from Table 1 on p58.  “There were no major imbalances in demographic or clinical characteristics between the combination and mADM only group (see Table 1), except for type of antidepressant.”  [Results, p56] |
| Authors risk-of-bias judgement of domain: | **LOW** | |
| *Domain 2: Deviations from intended interventions* | | |
| **Signaling Question** | **Response** | **Description** |
| 2.1. Were participants aware of their assigned intervention during the trial? | Y | Awareness of the assigned interventions is inherent to the design of the trial and the intervention examined.  From the study protocol, Huijbers et al. 2012: “The research assistant then communicates the treatment allocation to the patient, which means that he or she is no longer blind to the treatment condition. Unblinding of patients and research-assistants could not be avoided because the different conditions required different arrangements for treatment appointments, and separating this task from the assessments was logistically impossible in most research centers.” [Randomization, p7] |
| 2.2. Were carers and people delivering the interventions aware of participants' assigned intervention during the trial? | Y |  |
| 2.3. If Y/PY/NI to 2.1 or 2.2: Were there deviations from the intended intervention that arose because of the trial context? | NI | “In the MBCT+mADM condition, 28/33 patients (85%) completed MBCT. In the mADM condition, 8/35 patients (23%) received MBCT (protocol violation). Adherence to mADM did not differ between MBCT+mADM (n=28/33, 85%) and mADM (n= 28/35, 80%).” [Results, p57]. However, this seems to be an error and not something that arose due to the trial context, and if this introduced bias it would probably be in favor of the control condition. |
| 2.4. If Y/PY to 2.3: Were these deviations likely to have affected the outcome? | n/a | n/a |
| 2.5. If Y/PY/NI to 2.4: Were these deviations from intended intervention balanced between groups? | n/a | n/a |
| 2.6. Was an appropriate analysis used to estimate the effect of assignment to intervention? | Y | “In line with the protocol, we primarily report analyses based on intention-to-treat (ITT), followed by per-protocol (PP) analyses.” [Statistical analysis, p56] |
| 2.7. If N/PN/NI to 2.6: Was there potential for a substantial impact (on the result) of the failure to analyse participants in the group to which they were randomized? | n/a | n/a |
| Authors risk-of-bias judgement of domain: | **SOME CONCERNS** | |
| *Domain 3: Risk of bias due to missing outcome data* | | |
| **Signaling Question** | **Response** | **Description** |
| 3.1. Were data for this outcome available for all, or nearly all, participants randomized? | PN | In total, 68 patients were randomized. From the flowchart in Figure 1 we gather that at 12 months 10 participants were lost to follow-up. This means that data is available for approximately 85%, which is judged as borderline insufficient by the reviewers for this dichotomous outcome. |
| 3.2. If N/PN/NI to 3.1: Is there evidence that the result was not biased by missing outcome data? | NI | No evidence that results were not biased, as no bias correcting analysis methods or adequate sensitivity analyses are apparent to the review team |
| 3.3. If N/PN to 3.2: Could missingness in the outcome depend on its true value? | PY | It is possible that dropout, while somewhat comparable across groups, is related to participants depression status and it could thus be that missingness was influenced by its true value.  However, this is not judged as likely by the reviewers, as differences in dropout were small and there were no significant differences found between groups on outcome measures. |
| 3.4. If Y/PY/NI to 3.3: Is it likely that missingness in the outcome depended on its true value? | PN |  |
| Authors risk-of-bias judgement of domain: | **SOME CONCERNS** | |
| *Domain 4: Risk of bias in measurement of the outcome* | | |
| **Signaling Question** | **Response** | **Description** |
| 4.1. Was the method of measuring the outcome inappropriate? | N | “The primary outcome measure was relapse/recurrence as measured with the SCID-I by trained research assistants every three months during the 15-month follow-up period. We purposively selected a sample of audio taped SCID-I interviews (n¼35) across all study centres and across different levels of depression severity (mild, moderate, or severe). These were rated by blind raters. The inter-rater agreement between first and second ratings was found to be substantial (Landis and Koch, 1977), κ= 0.70, 95% confidence interval 0.46–0.94.”  [2.5.1. Primary outcome, p56]  Using the SCID-I to judge relapse is considered suitable for this outcome, by the review team. |
| 4.2. Could measurement or ascertainment of the outcome have differed between intervention groups? | N | Same measurement methods and thresholds were used, at comparable time points.  “Follow-up assessments took place 3, 6, 9, 12, and 15 months after this start date.” [2.3. Procedure, p55] |
| 4.3. If N/PN/NI to 4.1 and 4.2: Were outcome assessors aware of the intervention received by study participants? | Y | “The research assistants conducting the assessments could not be blinded to treatment condition since they were also involved in the practical organisation of the trial.” [2.3. Procedure, p55]  From the study protocol, Huijbers et al. 2012: “To assess the reliability of the follow-up assessments, all interviews are audio taped and a random selection of actual, borderline or probable cases of relapse/recurrence is rated by an independent assistant blind to treatment allocation.” [Randomization, p7] |
| 4.4. If Y/PY/NI to 4.3: Could assessment of the outcome have been influenced by knowledge of intervention received? | Y | Yes, it is possible that knowledge of treatment allocation could have influenced the assessment, as the research assistent conducting the follow-up assessment was to come to a judgement (relapse vs. no relapse), as opposed to a more objective outcome such as all-cause mortality.  However, there is not enough information available to believe that undue influence was likely. That is, there is no strong level of belief that outcomes were influenced and not enough information available. NB. If bias was introduced, it is likely to be in favor of the intervention group. |
| 4.5. If Y/PY/NI to 4.4: Is it likely that assessment of the outcome was influenced by knowledge of intervention received? | NI |  |
| Authors risk-of-bias judgement of domain: | **HIGH** | |
| *Domain 5: Risk of bias in selection of the reported result* | | |
| **Signaling Question** | **Response** | **Description** |
| 5.1. Were the data that produced this result analysed in accordance with a pre-specified analysis plan that was finalized before unblinded outcome data were available for analysis? | Y | Both a preregistration and prespecified protocol are available.  ClinicalTrials.gov: NCT00928980  Huijbers, M. J., Spijker, J., Donders, A. R. T., van Schaik, D. J., van Oppen, P., Ruhé, H. G., ... & Speckens, A. E. (2012). Preventing relapse in recurrent depression using mindfulness-based cognitive therapy, antidepressant medication or the combination: trial design and protocol of the MOMENT study. BMC psychiatry, 12, 1-11.  Researchers did indeed pre-specify their intentions in sufficient detail, and they match the published manuscript. |
| Is the numerical result being assessed likely to have been selected, on the basis of the results, from... | | |
| 5.2. ...multiple eligible outcome measurements (e.g. scales, definitions, time points) within the outcome domain? | PN | All eligible reported results for the outcome measurement seem to correspond to all intended measurements. |
| 5.3. ...multiple eligible analyses of the data? | PN | All eligible reported results for the outcome measurement seem to correspond to all intended analyses. |
| Authors risk-of-bias judgement of domain: | **LOW** | |
| **Study: Jarrett et al. (2001)** | | |
| *Domain 1: Randomization Process* | | |
| **Signaling Question** | **Response** | **Description** |
| 1.1. Was the allocation sequence random? | Y | “Responders were randomized using PROC PLAN in SAS statistical software, version 6.04 (SAS Institute Inc, Cary, NC) by strata that included … Research personnel and patients concealed assignment from blind evaluators.”  [Patients and methods, p382] |
| 1.2. Was the allocation sequence concealed until participants were enrolled and assigned to interventions? | Y |  |
| 1.3. Did baseline differences between intervention groups suggest a problem with the randomization process? | N | No imbalances are apparent that signal problems with the randomization process and observed imbalances are compatible with chance, as can be gathered from the manuscript and information in the table on p384. |
| Authors risk-of-bias judgement of domain: | **LOW** | |
| *Domain 2: Deviations from intended interventions* | | |
| **Signaling Question** | **Response** | **Description** |
| 2.1. Were participants aware of their assigned intervention during the trial? | Y | Awareness of the assigned interventions is inherent to the design of the trial and the intervention examined, given that participants could be allocated to 10 sessions of C-CT or control (evaluation without CT). |
| 2.2. Were carers and people delivering the interventions aware of participants' assigned intervention during the trial? | Y |  |
| 2.3. If Y/PY/NI to 2.1 or 2.2: Were there deviations from the intended intervention that arose because of the trial context? | N | “Prescribed control procedures prohibited the use of CT or other psychosocial interventions before relapse” [Methods, p383]  “At randomization, all patients planned to defer psychiatric treatment until they experienced a relapse or recurrence or the study ended. Only 10 patients reported seeking psychiatric “treatment” lasting 2 or more weeks before relapse or recurrence. During the experimental phase, 2 patients reported “treatment”: 1 C-CT patient took methylphenidate hydrochloride (Ritalin) for 8 weeks and 1 control patient had 4 sessions of “peer support” for grief.” [Patient compliance, p385]  “During the follow-up phase and before relapse or recurrence, 8 patients (4 in C-CT and 4 in control) reported “treatment” for 2 or more weeks. One patient from C-CT who reported depressive symptoms also received 4 sessions of psychotherapy. The following treatment occurred during intervals in which the patients did not report depressive symptoms (ie, PSR score $4): 1 C-CT patient sought marital therapy with the cognitive therapist during longitudinal follow-up (36 sessions); 1 C-CT patient attended 2 sessions in a “women’s employment support group”; 2 patients (1 C-CT and 1 control) received 2 to 3 psychotherapy or family therapy sessions; 1 control patient had 19 sessions of marital therapy; 1 control patient took 5 conflict resolution classes with his partner; and 1 control patient reported taking melatonin for 3 weeks.” [Patient compliance, p385] |
| 2.4. If Y/PY to 2.3: Were these deviations likely to have affected the outcome? | n/a | n/a |
| 2.5. If Y/PY/NI to 2.4: Were these deviations from intended intervention balanced between groups? | n/a | n/a |
| 2.6. Was an appropriate analysis used to estimate the effect of assignment to intervention? | Y | “All analyses were of an intention-to-treat strategy…”[Statistical analyses, p383] |
| 2.7. If N/PN/NI to 2.6: Was there potential for a substantial impact (on the result) of the failure to analyse participants in the group to which they were randomized? | n/a | n/a |
| Authors risk-of-bias judgement of domain: | **LOW** | |
| *Domain 3: Risk of bias due to missing outcome data* | | |
| **Signaling Question** | **Response** | **Description** |
| 3.1. Were data for this outcome available for all, or nearly all, participants randomized? | N | In total, 84 participants were randomized to C-CT (n = 41) or control (n = 43). Outcome data was available for n = 60 (71.43%). [Figure 1, p385] |
| 3.2. If N/PN/NI to 3.1: Is there evidence that the result was not biased by missing outcome data? | PN | No evidence that results were not biased, as no bias correcting analysis methods or adequate sensitivity analyses are apparent to the review team. |
| 3.3. If N/PN to 3.2: Could missingness in the outcome depend on its true value? | PY | It is possible that dropout, while somewhat comparable across groups, is related to participants depression status and it could thus be that missingness was influenced by its true value. However, this is not judged as likely by the reviewers. |
| 3.4. If Y/PY/NI to 3.3: Is it likely that missingness in the outcome depended on its true value? | PN |  |
| Authors risk-of-bias judgement of domain: | **SOME CONCERNS** | |
| *Domain 4: Risk of bias in measurement of the outcome* | | |
| **Signaling Question** | **Response** | **Description** |
| 4.1. Was the method of measuring the outcome inappropriate? | N | “The primary dependent variables were the proportion of DSM-IV diagnoses of MDD (ie, relapse or recurrence) as specified by an evaluator who was blind to cell assignment and used the Longitudinal Interval Follow-up Evaluation25 (LIFE)” [Outcome measures, p383]  This is considered suitable to judge this outcome, by the review team. |
| 4.2. Could measurement or ascertainment of the outcome have differed between intervention groups? | N | “Blind evaluations were conducted at (1) the end of A-CT; (2) any time the patient, therapist, or follow-up evaluators suspected relapse or recurrence; (3) early exit; (4) months 4 and 8 of the experimental phase; and (5) months 12 and 24 of follow-up. Assessment for months 13 to 20 was conducted by unblinded evaluators because of the high cost of blind evaluations. If relapse or recurrence was suspected, a blind evaluation occurred.” [Outcome measures, p383] |
| 4.3. If N/PN/NI to 4.1 and 4.2: Were outcome assessors aware of the intervention received by study participants? | N | “Blind evaluations were conducted at (1) the end of A-CT; (2) any time the patient, therapist, or follow-up evaluators suspected relapse or recurrence; (3) early exit; (4) months 4 and 8 of the experimental phase; and (5) months 12 and 24 of follow-up. Assessment for months 13 to 20 was conducted by unblinded evaluators because of the high cost of blind evaluations. If relapse or recurrence was suspected, a blind evaluation occurred.” [Outcome measures, p383] |
| 4.4. If Y/PY/NI to 4.3: Could assessment of the outcome have been influenced by knowledge of intervention received? | n/a | n/a |
| 4.5. If Y/PY/NI to 4.4: Is it likely that assessment of the outcome was influenced by knowledge of intervention received? | n/a |  |
| Authors risk-of-bias judgement of domain: | **LOW** | |
| *Domain 5: Risk of bias in selection of the reported result* | | |
| **Signaling Question** | **Response** | **Description** |
| 5.1. Were the data that produced this result analysed in accordance with a pre-specified analysis plan that was finalized before unblinded outcome data were available for analysis? | NI | No detailed pre-specified intentions available to the review authors. Thus, planned outcome measures and analyses cannot be compared to those presented in the reviewed trials/articles. |
| Is the numerical result being assessed likely to have been selected, on the basis of the results, from... | | |
| 5.2. ...multiple eligible outcome measurements (e.g. scales, definitions, time points) within the outcome domain? | NI | Pre-specified definitions of outcomes are not available or reported in sufficient detail to facilitate the assessment and reach a valid judgement. |
| 5.3. ...multiple eligible analyses of the data? | NI | Analysis intentions are not available or reported in sufficient detail to facilitate the assessment and reach a valid judgement. |
| Authors risk-of-bias judgement of domain: | **SOME CONCERNS** | |
| **Study: Jarrett et al. (2013)** | | |
| *Domain 1: Randomization Process* | | |
| **Signaling Question** | **Response** | **Description** |
| 1.1. Was the allocation sequence random? | Y | “The study statistician used a computer program to randomize patients within strata (site, number of depressive episodes, and presence/absence of dysthymia); assignments were implemented by the study coordinators and research pharmacists (see footnotes B and C in the eAppendix in Supplement). Only dispensing pharmacists knew assignment to fluoxetine or PBO and could break the blind during a clinical emergency. The integrity of the randomization was confirmed by the study statistician.”  [Methods, p1154] |
| 1.2. Was the allocation sequence concealed until participants were enrolled and assigned to interventions? | Y |  |
| 1.3. Did baseline differences between intervention groups suggest a problem with the randomization process? | N | No imbalances are apparent that signal problems with the randomization process and observed imbalances are compatible with chance, as can be gathered from the manuscript and tables in the online supplemental materials. |
| Authors risk-of-bias judgement of domain: | **LOW** | |
| *Domain 2: Deviations from intended interventions* | | |
| **Signaling Question** | **Response** | **Description** |
| 2.1. Were participants aware of their assigned intervention during the trial? | PY | Awareness of the assigned interventions is inherent to the design of the trial and the intervention examined in this IPD-MA, given that participants could be allocated to C-CT or fluoxetine. The pill-placebo control group was not included in the current analysis, while participant technically could not be aware of their assignment to fluoxetine vs. pill-placebo, we judge that both participants and clinicians were mostly aware of the possible interventions (at least C-CT vs. fluoxetine or pill-placebo). |
| 2.2. Were carers and people delivering the interventions aware of participants' assigned intervention during the trial? | PY |  |
| 2.3. If Y/PY/NI to 2.1 or 2.2: Were there deviations from the intended intervention that arose because of the trial context? | PN | “Use of Concomitant Nonprotocol Therapies During the experimental phase, 13 randomized patients reported using nonstudy medications that might have had psychoactive effects (C-CT, n = 7; fluoxetine, n = 2; PBO, n = 5). In most cases (n = 8), the medication was an over-the-counter sleeping pill; 1 patient in the C-CT arm deviated from the protocol by taking an antidepressant prescribed by a primary care physician. Two patients (1 each in the fluoxetine and PBO arms) deviated from the protocol by attending self-help support groups. No relationships were evident between usage and treatment cell (according to χ2 tests) or between usage and relapse/ recurrence (according to Cox regression).” [Results, p1156] |
| 2.4. If Y/PY to 2.3: Were these deviations likely to have affected the outcome? | n/a | n/a |
| 2.5. If Y/PY/NI to 2.4: Were these deviations from intended intervention balanced between groups? | n/a | n/a |
| 2.6. Was an appropriate analysis used to estimate the effect of assignment to intervention? | Y | “All analyses used the intention-to-treat sample, with cumulative relapse/recurrence as the primary outcomes for the survival analysis.” [Statistical Analyses, p1155] |
| 2.7. If N/PN/NI to 2.6: Was there potential for a substantial impact (on the result) of the failure to analyse participants in the group to which they were randomized? | n/a | n/a |
| Authors risk-of-bias judgement of domain: | **LOW** | |
| *Domain 3: Risk of bias due to missing outcome data* | | |
| **Signaling Question** | **Response** | **Description** |
| 3.1. Were data for this outcome available for all, or nearly all, participants randomized? | PY | “Follow-up Of the 181 who entered follow-up (70 in C-CT, 62 in fluoxetine, and 49 in PBO), 145 (80.1%) completed at least 12 months (56 in C-CT, 52 in fluoxetine, and 37 in PBO) and 124 (n = 68.5%) completed 24 months (47 in C-CT, 44 in fluoxetine, and 33 in PBO). The percentage of patients who completed longitudinal follow-up did not differ significantly as a function of continuation phase treatments or between sites.”  [Patient Disposition, p1155] |
| 3.2. If N/PN/NI to 3.1: Is there evidence that the result was not biased by missing outcome data? | n/a | n/a |
| 3.3. If N/PN to 3.2: Could missingness in the outcome depend on its true value? | n/a | n/a |
| 3.4. If Y/PY/NI to 3.3: Is it likely that missingness in the outcome depended on its true value? | n/a |  |
| Authors risk-of-bias judgement of domain: | **LOW** | |
| *Domain 4: Risk of bias in measurement of the outcome* | | |
| **Signaling Question** | **Response** | **Description** |
| 4.1. Was the method of measuring the outcome inappropriate? | N | “Primary Outcomes: Relapse/Recurrence Relapse, which designates an exacerbation of the presenting episode after a response but before recovery,47 was defined by DSM-IV criteria for MDD(ie,LIFE PSR score of 5 or 6 for 2 consecutive weeks).” [Primary Outcomes: Relapse/Recurrence, p1155]  This is considered suitable to judge this outcome, by the review team. |
| 4.2. Could measurement or ascertainment of the outcome have differed between intervention groups? | PN | “Outcome Assessments At the end of months 4 and 8 of the experimental phase, an independent evaluator assessed DSM-IV criteria for MDD using the Structured Clinical Interview and Psychiatric Status Ratings (PSR)46 from the Longitudinal Interval Follow-up Evaluation (LIFE). Interim or emergency evaluations were also performed if a relapse/recurrence was suspected. Assessments were conducted without knowledge of treatment assignment. Infrequently, telephone assessments were performed when patients were not available for in-clinic assessments. During the longitudinal follow-up, all protocol treatments were discontinued and independent evaluators used the same methods to evaluate patients every 4 months (ie, 12, 16, 20, 24, 28, and 32 months after randomization). Patients were encouraged to contact study staff if they were experiencing depressive symptoms or worsening in some other way so that an interim blinded evaluation could be completed.” [Outcome Assessments, p1154] |
| 4.3. If N/PN/NI to 4.1 and 4.2: Were outcome assessors aware of the intervention received by study participants? | N | “Outcome Assessments At the end of months 4 and 8 of the experimental phase, an independent evaluator assessed DSM-IV criteria for MDD using the Structured Clinical Interview and Psychiatric Status Ratings (PSR)46 from the Longitudinal Interval Follow-up Evaluation (LIFE). Interim or emergency evaluations were also performed if a relapse/recurrence was suspected. Assessments were conducted without knowledge of treatment assignment.” [Outcome Assessments, p1154] |
| 4.4. If Y/PY/NI to 4.3: Could assessment of the outcome have been influenced by knowledge of intervention received? | n/a | n/a |
| 4.5. If Y/PY/NI to 4.4: Is it likely that assessment of the outcome was influenced by knowledge of intervention received? | n/a |  |
| Authors risk-of-bias judgement of domain: | **LOW** | |
| *Domain 5: Risk of bias in selection of the reported result* | | |
| **Signaling Question** | **Response** | **Description** |
| 5.1. Were the data that produced this result analysed in accordance with a pre-specified analysis plan that was finalized before unblinded outcome data were available for analysis? | Y | Both preregistrations and a detailed protocol paper are available.  Clinicaltrials.gov Identifiers: NCT00118404, NCT00183664, and NCT00218764  Jarrett, R. B., & Thase, M. E. (2010). Comparative efficacy and durability of continuation phase cognitive therapy for preventing recurrent depression: design of a double-blinded, fluoxetine-and pill placebo-controlled, randomized trial with 2-year follow-up. Contemporary Clinical Trials, 31(4), 355-377.  Researchers did indeed pre-specify their intentions in sufficient detail, and they match the published manuscript. |
| Is the numerical result being assessed likely to have been selected, on the basis of the results, from... | | |
| 5.2. ...multiple eligible outcome measurements (e.g. scales, definitions, time points) within the outcome domain? | PN | All eligible reported results for the outcome measurement seem to correspond to all intended measurements. |
| 5.3. ...multiple eligible analyses of the data? | PN | All eligible reported results for the outcome measurement seem to correspond to all intended analyses. |
| Authors risk-of-bias judgement of domain: | **LOW** | |
| **Study: de Jonge et al. (2019)** | | |
| *Domain 1: Randomization Process* | | |
| **Signaling Question** | **Response** | **Description** |
| 1.1. Was the allocation sequence random? | Y | “An independent researcher using a computer-generated random schedule undertook randomization at the patient level.”  [Method, p523]  “The randomization was accessible solely by the independent researcher.”  [Method, p523] |
| 1.2. Was the allocation sequence concealed until participants were enrolled and assigned to interventions? | Y |  |
| 1.3. Did baseline differences between intervention groups suggest a problem with the randomization process? | N | No baseline differences apparent, as can be gathered from the manuscript or seen in Table 1 on p.526.  “As shown in Table 1, the treatment conditions were comparable on all characteristics; there were no significant differences between the conditions.”  [Results, p524] |
| Authors risk-of-bias judgement of domain: | **LOW** | |
| *Domain 2: Deviations from intended interventions* | | |
| **Signaling Question** | **Response** | **Description** |
| 2.1. Were participants aware of their assigned intervention during the trial? | Y | Awareness of the assigned interventions is inherent to the design of the trial and the intervention examined, given that participants could be allocated to PCT or treatment-as-usual. |
| 2.2. Were carers and people delivering the interventions aware of participants' assigned intervention during the trial? | Y |  |
| 2.3. If Y/PY/NI to 2.1 or 2.2: Were there deviations from the intended intervention that arose because of the trial context? | NI | Not enough data available to judge whether deviations arouse because of the trial context. |
| 2.4. If Y/PY to 2.3: Were these deviations likely to have affected the outcome? | n/a | n/a |
| 2.5. If Y/PY/NI to 2.4: Were these deviations from intended intervention balanced between groups? | n/a | n/a |
| 2.6. Was an appropriate analysis used to estimate the effect of assignment to intervention? | Y | “The data was analyzed by intention to treat, including all randomized subjects in the study. Patients who dropped out were considered censored at the last evaluation.”  [ Statistical analysis, p524] |
| 2.7. If N/PN/NI to 2.6: Was there potential for a substantial impact (on the result) of the failure to analyse participants in the group to which they were randomized? | n/a | n/a |
| Authors risk-of-bias judgement of domain: | **SOME CONCERNS** | |
| *Domain 3: Risk of bias due to missing outcome data* | | |
| **Signaling Question** | **Response** | **Description** |
| 3.1. Were data for this outcome available for all, or nearly all, participants randomized? | Y | In total, 214 patients were randomized to CAU or CAU+PCT. Across both groups a total of 195 participants completed the 15 months follow-up (91.1%) and data availability is considered adequate. |
| 3.2. If N/PN/NI to 3.1: Is there evidence that the result was not biased by missing outcome data? | n/a | n/a |
| 3.3. If N/PN to 3.2: Could missingness in the outcome depend on its true value? | n/a | n/a |
| 3.4. If Y/PY/NI to 3.3: Is it likely that missingness in the outcome depended on its true value? | n/a |  |
| Authors risk-of-bias judgement of domain: | **LOW** | |
| *Domain 4: Risk of bias in measurement of the outcome* | | |
| **Signaling Question** | **Response** | **Description** |
| 4.1. Was the method of measuring the outcome inappropriate? | N | The primary outcome measure was time-to-relapse/recurrence of depression meeting DSM–IV criteria for an MDE according to the SCID-I over a 15-month follow-up (Spitzer et al., 1992). Although we do not have reliability data from our own study, the SCID-I is considered the gold standard in semistructured instruments for depression. For the Dutch version of the SCID-I, the interrater reliability has been shown to be fair to excellent (Lobbestael, Leurgans, & Arntz, 2011). [Primary Outcome, p524]  Using the SCID-I to judge relapse is considered suitable for this outcome, by the review team. |
| 4.2. Could measurement or ascertainment of the outcome have differed between intervention groups? | PN | “The SCID-I was administered at baseline and at follow-up 15 months later.” [Primary Outcome, p524] |
| 4.3. If N/PN/NI to 4.1 and 4.2: Were outcome assessors aware of the intervention received by study participants? | N | “All interviews were done by trained assessors who were “blind” to treatment allocation and who attended regular consensus meetings to enhance interrater agreement. After the interview, the assessors were asked to guess the treatment allocation. The assessors guessed correctly in 52.3% of the cases, which is what was to be expected based on chance.” [Randomization and Masking, p523] |
| 4.4. If Y/PY/NI to 4.3: Could assessment of the outcome have been influenced by knowledge of intervention received? | n/a | n/a |
| 4.5. If Y/PY/NI to 4.4: Is it likely that assessment of the outcome was influenced by knowledge of intervention received? | n/a |  |
| Authors risk-of-bias judgement of domain: | **LOW** | |
| *Domain 5: Risk of bias in selection of the reported result* | | |
| **Signaling Question** | **Response** | **Description** |
| 5.1. Were the data that produced this result analysed in accordance with a pre-specified analysis plan that was finalized before unblinded outcome data were available for analysis? | Y | Trial registry (https://trialsearch.who.int/Trial2.aspx?TrialI  D=NTR2599) and the published protocol paper were consulted. The trial register was pre-registered on 2010-11-11 and the protocol paper published in 2015. See de Jonge et al. (2015). Preventive cognitive therapy versus treatment as usual in preventing recurrence of depression: Protocol of a multi-centered randomized controlled trial. BMC Psychiatry, 15, 139. http://dx.doi.org/10 .1186/s12888-015-0508-8 |
| Is the numerical result being assessed likely to have been selected, on the basis of the results, from... | | |
| 5.2. ...multiple eligible outcome measurements (e.g. scales, definitions, time points) within the outcome domain? | PN | All eligible reported results for the outcome measurement seem to correspond to all intended measurements. |
| 5.3. ...multiple eligible analyses of the data? | PN | All eligible reported results for the outcome measurement seem to correspond to all intended analyses. Additionally, analyses are consistent across several different reports on this trial. |
| Authors risk-of-bias judgement of domain: | **LOW** | |
| **Study: Klein et al. (2004)** | | |
| *Domain 1: Randomization Process* | | |
| **Signaling Question** | **Response** | **Description** |
| 1.1. Was the allocation sequence random? | NI | Only information available to reviewers about the randomization methods is the statement that the study is randomized.  “…continuation phase CBASP were randomized to monthly CBASP or assessment only for 1 year.”  [Abstract, p681] |
| 1.2. Was the allocation sequence concealed until participants were enrolled and assigned to interventions? | NI |  |
| 1.3. Did baseline differences between intervention groups suggest a problem with the randomization process? | N | No baseline imbalances apparent that signal a problem with the randomization procedure. Groups are mostly comparable.  “Second, we compared the 42 patients who were randomized to continue CBASP in the maintenance phase to the 40 patients who were randomized to the assessment only condition on demographic and clinical features. At entry into the acute phase of the study, patients ultimately assigned to the CBASP and assessment only conditions did not differ on age, race, marital status, type of chronic depression diagnosis, HRSD-24 score, age of onset of MDD, duration of index MDD episode, lifetime number of episodes of MDD, history of anxiety disorder, and history of substance use disorder (see Table 2). In addition, the two groups did not differ on HRSD-24 and IDS-SR-30 scores at maintenance phase baseline. Despite randomization, the two groups differed on gender, with a significantly greater proportion of women receiving CBASP, X2(1, N = 82) 6.28, p = .01.”  [Results, p683] |
| Authors risk-of-bias judgement of domain: | **SOME CONCERNS** | |
| *Domain 2: Deviations from intended interventions* | | |
| **Signaling Question** | **Response** | **Description** |
| 2.1. Were participants aware of their assigned intervention during the trial? | Y | Awareness of the assigned interventions is inherent to the design of the trial and the intervention examined, given that participants could be allocated to CBASP or assessment only. |
| 2.2. Were carers and people delivering the interventions aware of participants' assigned intervention during the trial? | Y |  |
| 2.3. If Y/PY/NI to 2.1 or 2.2: Were there deviations from the intended intervention that arose because of the trial context? | NI | “In both conditions, all psychotropic medication and nonprotocol psychotherapy were prohibited.” [Treatment Conditions, p682]  No adequate judgement can be made, as trialist did not report specifically whether deviations arose because of trial context. Not clear to reviewers. |
| 2.4. If Y/PY to 2.3: Were these deviations likely to have affected the outcome? | n/a | n/a |
| 2.5. If Y/PY/NI to 2.4: Were these deviations from intended intervention balanced between groups? | n/a | n/a |
| 2.6. Was an appropriate analysis used to estimate the effect of assignment to intervention? | Y | ITT analysis seems to be used.  “Patients who failed to complete the maintenance phase were included in these analyses using all available data up to the time of exiting the study.” [Recurrence, p684] |
| 2.7. If N/PN/NI to 2.6: Was there potential for a substantial impact (on the result) of the failure to analyse participants in the group to which they were randomized? | n/a | n/a |
| Authors risk-of-bias judgement of domain: | **SOME CONCERNS** | |
| *Domain 3: Risk of bias due to missing outcome data* | | |
| **Signaling Question** | **Response** | **Description** |
| 3.1. Were data for this outcome available for all, or nearly all, participants randomized? | PN | In total, 82 participants were allocated to either CBASP or an assessment only condition. “Seventh, 21 of the 82 patients (25.6%) entering the maintenance phase dropped out of the study at some point before recurrence or completion. Although we used data analytic techniques that made use of all data up to the point of dropout, we cannot rule out the possibility that the findings were biased in unknown ways by these early exits” [Discussion, p687] |
| 3.2. If N/PN/NI to 3.1: Is there evidence that the result was not biased by missing outcome data? | PN | No evidence that results were not biased, as no bias correcting analysis methods or adequate sensitivity analyses are apparent to the review team. |
| 3.3. If N/PN to 3.2: Could missingness in the outcome depend on its true value? | PY | It is possible that dropout, while somewhat comparable across groups, is related to participants depression status and it could thus be that missingness was influenced by its true value. However, this is not judged as likely by the reviewers. |
| 3.4. If Y/PY/NI to 3.3: Is it likely that missingness in the outcome depended on its true value? | PN |  |
| Authors risk-of-bias judgement of domain: | **SOME CONCERNS** | |
| *Domain 4: Risk of bias in measurement of the outcome* | | |
| **Signaling Question** | **Response** | **Description** |
| 4.1. Was the method of measuring the outcome inappropriate? | PN | “The primary outcome measure throughout all phases of the study was the HRSD-24, which was administered by certified raters who were unaware of patients’ treatment conditions (Keller et al., 2000). The HRSD-24 was supplemented by a self-rated measure, the 30-item Inventory of Depressive Symptoms, Self-Report version (IDS-SR-30; Rush, Guillion, Basco, Jarrett, & Trivedi, 1996). Both measures were administered every 4 weeks during the maintenance phase. If depressive symptoms began to emerge, as evidenced by a HRSD-24 score of 16 or greater, another evaluation was scheduled within 2 weeks. Evaluations continued every 2 weeks until either the symptoms subsided or protocol recurrence criteria were met. Recurrence was defined in the protocol as a HRSD-24 score of 16 or greater on two consecutive visits and a diagnosis of MDD as determined from a DSM–IV MDD checklist administered by the independent evaluator.” [Measures, p683] |
| 4.2. Could measurement or ascertainment of the outcome have differed between intervention groups? | PN | “… Both measures were administered every 4 weeks during the maintenance phase. If depressive symptoms began to emerge, as evidenced by a HRSD-24 score of 16 or greater, another evaluation was scheduled within 2 weeks. Evaluations continued every 2 weeks until either the symptoms subsided or protocol recurrence criteria were met. Recurrence was defined in the protocol as a HRSD-24 score of 16 or greater on two consecutive visits and a diagnosis of MDD as determined from a DSM–IV MDD checklist administered by the independent evaluator.” [Measures, p683] |
| 4.3. If N/PN/NI to 4.1 and 4.2: Were outcome assessors aware of the intervention received by study participants? | PN | “The primary outcome measure throughout all phases of the study was the HRSD-24, which was administered by certified raters who were unaware of patients’ treatment conditions” [Measures, p683] |
| 4.4. If Y/PY/NI to 4.3: Could assessment of the outcome have been influenced by knowledge of intervention received? | n/a | n/a |
| 4.5. If Y/PY/NI to 4.4: Is it likely that assessment of the outcome was influenced by knowledge of intervention received? | n/a |  |
| Authors risk-of-bias judgement of domain: | **LOW** | |
| *Domain 5: Risk of bias in selection of the reported result* | | |
| **Signaling Question** | **Response** | **Description** |
| 5.1. Were the data that produced this result analysed in accordance with a pre-specified analysis plan that was finalized before unblinded outcome data were available for analysis? | NI | No detailed pre-specified intentions available to the review authors. Thus, planned outcome measures and analyses cannot be compared to those presented in the reviewed trials/articles. |
| Is the numerical result being assessed likely to have been selected, on the basis of the results, from... | | |
| 5.2. ...multiple eligible outcome measurements (e.g. scales, definitions, time points) within the outcome domain? | NI | Pre-specified definitions of outcomes are not available or reported in sufficient detail to facilitate the assessment and reach a valid judgement. |
| 5.3. ...multiple eligible analyses of the data? | NI | Analysis intentions are not available or reported in sufficient detail to facilitate the assessment and reach a valid judgement. |
| Authors risk-of-bias judgement of domain: | **SOME CONCERNS** | |
| **Study: Klein et al. (2018)** | | |
| *Domain 1: Randomization Process* | | |
| **Signaling Question** | **Response** | **Description** |
| 1.1. Was the allocation sequence random? | Y | “Simple randomization was undertaken (1:1 ratio) by an independent researcher using computer-generated random numbers with STATA”  [Manuscript, p55] |
| 1.2. Was the allocation sequence concealed until participants were enrolled and assigned to interventions? | Y |  |
| 1.3. Did baseline differences between intervention groups suggest a problem with the randomization process? | N | “Baseline characteristics in the ITT sample and of participants with follow-up data were comparable and balanced over treatment conditions, except for a slight imbalance in gender and severity of the last depressive episode.”  No significant baseline imbalances apparent to reviewers. |
| Authors risk-of-bias judgement of domain: | **LOW** | |
| *Domain 2: Deviations from intended interventions* | | |
| **Signaling Question** | **Response** | **Description** |
| 2.1. Were participants aware of their assigned intervention during the trial? | Y | Awareness of the assigned interventions is inherent to the design of the trial and the intervention examined, given that participants could be allocated to mPCT + TAU vs TAU only. |
| 2.2. Were carers and people delivering the interventions aware of participants' assigned intervention during the trial? | Y |  |
| 2.3. If Y/PY/NI to 2.1 or 2.2: Were there deviations from the intended intervention that arose because of the trial context? | NI | Not enough information available for the reviewers to make an adequate judgement; trialist did not adequately report this in the published manuscript or the online supplemental materials that we were able to review. |
| 2.4. If Y/PY to 2.3: Were these deviations likely to have affected the outcome? | n/a | n/a |
| 2.5. If Y/PY/NI to 2.4: Were these deviations from intended intervention balanced between groups? | n/a | n/a |
| 2.6. Was an appropriate analysis used to estimate the effect of assignment to intervention? | Y | “The intention-to-treat (ITT) principle was used, analyzing all participants regardless of adherence to the randomized condition.” [p55] |
| 2.7. If N/PN/NI to 2.6: Was there potential for a substantial impact (on the result) of the failure to analyse participants in the group to which they were randomized? | n/a | n/a |
| Authors risk-of-bias judgement of domain: | **SOME CONCERNS** | |
| *Domain 3: Risk of bias due to missing outcome data* | | |
| **Signaling Question** | **Response** | **Description** |
| 3.1. Were data for this outcome available for all, or nearly all, participants randomized? | PN | “Overall, 29 participants dropped out immediately after randomization and 24 were lost to follow-up.” [p55]  In total, 264 were randomized to either mPCT+ TAU or TAU only. Of these, 103 in the mPCT+ TAU group and 99 in the TAU only group had completed the 12-month follow-up, with 110 and 101 respectively at the 24 month follow-up. This is equal to approximately 76.52%. |
| 3.2. If N/PN/NI to 3.1: Is there evidence that the result was not biased by missing outcome data? | PN | No evidence that results were not biased, as no bias correcting analysis methods or adequate sensitivity analyses are apparent to the review team. |
| 3.3. If N/PN to 3.2: Could missingness in the outcome depend on its true value? | PY | It is possible that dropout, while somewhat comparable across groups, is related to participants depression status and it could thus be that missingness was influenced by its true value. However, this is not judged as likely by the reviewers. |
| 3.4. If Y/PY/NI to 3.3: Is it likely that missingness in the outcome depended on its true value? | PN |  |
| Authors risk-of-bias judgement of domain: | **SOME CONCERNS** | |
| *Domain 4: Risk of bias in measurement of the outcome* | | |
| **Signaling Question** | **Response** | **Description** |
| 4.1. Was the method of measuring the outcome inappropriate? | N | “The primary outcome was time-related proportion of relapse/recurrence according to the DSM-IV assessed with the SCID-I by blinded interviewers after 3, 12, and 24 months.”  This is considered suitable to judge this outcome, by the review team. |
| 4.2. Could measurement or ascertainment of the outcome have differed between intervention groups? | PN | “… blinded interviewers after 3, 12, and 24 months.” |
| 4.3. If N/PN/NI to 4.1 and 4.2: Were outcome assessors aware of the intervention received by study participants? | PN | “… blinded interviewers after 3, 12, and 24 months.” |
| 4.4. If Y/PY/NI to 4.3: Could assessment of the outcome have been influenced by knowledge of intervention received? | n/a | n/a |
| 4.5. If Y/PY/NI to 4.4: Is it likely that assessment of the outcome was influenced by knowledge of intervention received? | n/a |  |
| Authors risk-of-bias judgement of domain: | **LOW** | |
| *Domain 5: Risk of bias in selection of the reported result* | | |
| **Signaling Question** | **Response** | **Description** |
| 5.1. Were the data that produced this result analysed in accordance with a pre-specified analysis plan that was finalized before unblinded outcome data were available for analysis? | Y | Both preregistrations and a detailed protocol paper are available, with a statistical analysis plan (SAP) as an appendix to the protocol.  Trial registration: NTR2503  Bockting, C. L., Kok, G. D., Kamp, L. V. D., Smit, F., van Valen, E., Schoevers, R., ... & Beck, A. T. (2011). Disrupting the rhythm of depression using Mobile Cognitive Therapy for recurrent depression: randomized controlled trial design and protocol. BMC psychiatry, 11(1), 1-9.  Researchers did indeed pre-specify their intentions in sufficient detail, and they match the published manuscript. |
| Is the numerical result being assessed likely to have been selected, on the basis of the results, from... | | |
| 5.2. ...multiple eligible outcome measurements (e.g. scales, definitions, time points) within the outcome domain? | PN | All eligible reported results for the outcome measurement seem to correspond to all intended measurements. |
| 5.3. ...multiple eligible analyses of the data? | PN | All eligible reported results for the outcome measurement seem to correspond to all intended analyses. |
| Authors risk-of-bias judgement of domain: | **LOW** | |
| **Study: Ma et al. (2004)** | | |
| *Domain 1: Randomization Process* | | |
| **Signaling Question** | **Response** | **Description** |
| 1.1. Was the allocation sequence random? | NI | “Randomization of patients to treatment condition was by a statistician, who was not part of the research team, on receipt of the participant’s date of birth, gender, date of assessment, number of previous episodes of depression, and severity of last episode.”  [Method, p32]  However, not enough information available to reviewers with regard to the randomization sequence used. |
| 1.2. Was the allocation sequence concealed until participants were enrolled and assigned to interventions? | Y |  |
| 1.3. Did baseline differences between intervention groups suggest a problem with the randomization process? | N | “Table 1 shows baseline characteristics of the intent-to-treat sample. TAU and MBCT groups were closely similar on each of the baseline variables.”  [Results, p34]  No significant baseline imbalances apparent that could indicate randomization problems. |
| Authors risk-of-bias judgement of domain: | **LOW** | |
| *Domain 2: Deviations from intended interventions* | | |
| **Signaling Question** | **Response** | **Description** |
| 2.1. Were participants aware of their assigned intervention during the trial? | Y | Awareness of the assigned interventions is inherent to the design of the trial and the intervention examined, given that participants could be allocated to MBCT + TAU vs TAU only. |
| 2.2. Were carers and people delivering the interventions aware of participants' assigned intervention during the trial? | Y |  |
| 2.3. If Y/PY/NI to 2.1 or 2.2: Were there deviations from the intended intervention that arose because of the trial context? | NI | “The treatment patients in the TAU and MBCT groups received was monitored at the 3-month assessment sessions and is described in the Results section.” [Method, p34]  “Treatment Received Nontrial treatment for depression was monitored in the trimonthly interviews. There were no significant differences between the TAU and MBCT groups for any of the measures of nontrial treatment received (all ps > .10; see Table 2).”  [Results, p35]  From Table 2 in the published manuscript we gather that patients in the TAU condition used both medication for depression and counseling/psychotherapy at a higher frequency. While this may be considered part of TAU, this could possibly be due to the trial context. However, not enough information available for the reviewers to make an adequate judgement. |
| 2.4. If Y/PY to 2.3: Were these deviations likely to have affected the outcome? | n/a | n/a |
| 2.5. If Y/PY/NI to 2.4: Were these deviations from intended intervention balanced between groups? | n/a | n/a |
| 2.6. Was an appropriate analysis used to estimate the effect of assignment to intervention? | Y | “Normally, outcome analyses would be conducted for both a per-protocol sample (which would comprise all patients allocated to TAU, together with those patients allocated to MBCT who received a “minimally adequate dose” of MBCT by attending at least four MBCT sessions) and an intent-to-treat sample (which would comprise all patients included in the random allocation). However, in our study, for individuals with three or more episodes, the per-protocol and intent-to-treat samples differed by only 1 participant. Hence, it was deemed unnecessary to conduct analyses for both samples, and only the intent-to-treat sample was analyzed. For those with only two episodes, analyses for both samples were conducted.”  [Results, p34] |
| 2.7. If N/PN/NI to 2.6: Was there potential for a substantial impact (on the result) of the failure to analyse participants in the group to which they were randomized? | n/a | n/a |
| Authors risk-of-bias judgement of domain: | **SOME CONCERNS** | |
| *Domain 3: Risk of bias due to missing outcome data* | | |
| **Signaling Question** | **Response** | **Description** |
| 3.1. Were data for this outcome available for all, or nearly all, participants randomized? | Y | In total, 75 participants were randomized. “Complete data on relapse/recurrence were available for 73 (97%) of the 75 patients in the intent-to-treat sample…” [Results, p34] |
| 3.2. If N/PN/NI to 3.1: Is there evidence that the result was not biased by missing outcome data? | n/a | n/a |
| 3.3. If N/PN to 3.2: Could missingness in the outcome depend on its true value? | n/a | n/a |
| 3.4. If Y/PY/NI to 3.3: Is it likely that missingness in the outcome depended on its true value? | n/a |  |
| Authors risk-of-bias judgement of domain: | **LOW** | |
| *Domain 4: Risk of bias in measurement of the outcome* | | |
| **Signaling Question** | **Response** | **Description** |
| 4.1. Was the method of measuring the outcome inappropriate? | N | “Relapse/Recurrence The primary treatment outcome variable was the occurrence of relapse or recurrence meeting DSM–IV criteria for major depressive episode (American Psychiatric Association, 1994). Assessments were modeled on the Structured Clinical Interview for DSM–III–R (Spitzer, Williams, Gibbon, & First, 1992) and done by a clinical psychologist blind to patients’ treatment condition.” [Method, p33] |
| 4.2. Could measurement or ascertainment of the outcome have differed between intervention groups? | PN | “Following baseline assessment, interviews were scheduled at points corresponding to the completion of the initial eight MBCT training sessions, and every 3 months thereafter over the course of the follow-up year.” [Method, p33] |
| 4.3. If N/PN/NI to 4.1 and 4.2: Were outcome assessors aware of the intervention received by study participants? | PN | “… done by a clinical psychologist blind to patients’ treatment condition. To examine interrater reliability, we audiotaped interviews, and those in which patients met screening criteria for major depression were evaluated by an independent, blind, experienced research psychiatrist. Any information that might prejudice blindness was erased from the tapes presented to the independent assessor.” [Method, p33] |
| 4.4. If Y/PY/NI to 4.3: Could assessment of the outcome have been influenced by knowledge of intervention received? | n/a | n/a |
| 4.5. If Y/PY/NI to 4.4: Is it likely that assessment of the outcome was influenced by knowledge of intervention received? | n/a |  |
| Authors risk-of-bias judgement of domain: | **LOW** | |
| *Domain 5: Risk of bias in selection of the reported result* | | |
| **Signaling Question** | **Response** | **Description** |
| 5.1. Were the data that produced this result analysed in accordance with a pre-specified analysis plan that was finalized before unblinded outcome data were available for analysis? | NI | No detailed pre-specified intentions available to the review authors. Thus, planned outcome measures and analyses cannot be compared to those presented in the reviewed trials/articles. |
| Is the numerical result being assessed likely to have been selected, on the basis of the results, from... | | |
| 5.2. ...multiple eligible outcome measurements (e.g. scales, definitions, time points) within the outcome domain? | NI | Pre-specified definitions of outcomes are not available or reported in sufficient detail to facilitate the assessment and reach a valid judgement. |
| 5.3. ...multiple eligible analyses of the data? | NI | Analysis intentions are not available or reported in sufficient detail to facilitate the assessment and reach a valid judgement. |
| Authors risk-of-bias judgement of domain: | **SOME CONCERNS** | |
| **Study: Moore et al. (2022)** | | |
| *Domain 1: Randomization Process* | | |
| **Signaling Question** | **Response** | **Description** |
| 1.1. Was the allocation sequence random? | Y | “A research assistant assigned participants to groups with randomization achieved by computer-generated random number sequencing”  However, not enough information available to reviewers about the concealment of sequences until enrollment and assignment were completed. Randomization was conducted by a research assistant, but no remote or centrally administered method to allocate seems to be used.  [Method, p11] |
| 1.2. Was the allocation sequence concealed until participants were enrolled and assigned to interventions? | NI |  |
| 1.3. Did baseline differences between intervention groups suggest a problem with the randomization process? | N | No imbalances apparent and any observed imbalances are compatible with chance. |
| Authors risk-of-bias judgement of domain: | **SOME CONCERNS** | |
| *Domain 2: Deviations from intended interventions* | | |
| **Signaling Question** | **Response** | **Description** |
| 2.1. Were participants aware of their assigned intervention during the trial? | Y | Awareness of the assigned interventions is inherent to the design of the trial and the intervention examined, given that participants could be allocated to MBCT, TAU or RGT. |
| 2.2. Were carers and people delivering the interventions aware of participants' assigned intervention during the trial? | Y |  |
| 2.3. If Y/PY/NI to 2.1 or 2.2: Were there deviations from the intended intervention that arose because of the trial context? | NI | Not enough information available for the reviewers to make an adequate judgement; trialist did not report this in the published manuscript. |
| 2.4. If Y/PY to 2.3: Were these deviations likely to have affected the outcome? | n/a | n/a |
| 2.5. If Y/PY/NI to 2.4: Were these deviations from intended intervention balanced between groups? | n/a | n/a |
| 2.6. Was an appropriate analysis used to estimate the effect of assignment to intervention? | Y | “All analyses were conducted on the intention to treat (ITT) sample and are reported below.” [Results, p17] |
| 2.7. If N/PN/NI to 2.6: Was there potential for a substantial impact (on the result) of the failure to analyse participants in the group to which they were randomized? | n/a | n/a |
| Authors risk-of-bias judgement of domain: | **SOME CONCERNS** | |
| *Domain 3: Risk of bias due to missing outcome data* | | |
| **Signaling Question** | **Response** | **Description** |
| 3.1. Were data for this outcome available for all, or nearly all, participants randomized? | PN | In total, 227 patients were randomized to either one of the 3 conditions. At follow-up, 25 were lost in the MBCT group, 37 in the RGT group and 16 in the TAU group, totaling 78 participants. Thus, data was available at follow-up for your primary outcome for about 65% |
| 3.2. If N/PN/NI to 3.1: Is there evidence that the result was not biased by missing outcome data? | PN | No evidence that results were not biased, as no bias correcting analysis methods or adequate sensitivity analyses are apparent to the review team. |
| 3.3. If N/PN to 3.2: Could missingness in the outcome depend on its true value? | PY | It is possible that dropout, while somewhat comparable across groups, is related to participants depression status and it could thus be that missingness was influenced by its true value.  “With regard to completion of the follow-up assessment, comparison of all three groups was statistically significant, Χ2 (2, N = 227) = 12.38, p = .002, while the comparison of MBCT and RGT just missed this cut-off, Χ2 (1, N = 151) = 3.17, p = .075. Completion was highest for TAU participants (79%), followed by MBCT participants (66%), and RGT participants (52%). One possible explanation for higher completion rate for TAU participants may be due to the fact that MBCT treatment was offered to these participants at the end of the follow-up period, acting as an incentive for TAU participants to complete all waves of data collection.” [Missing data, p16]  In conclusion, there seem to be differences in the proportion of missing outcome data between groups, and is therefore judged likely that missingness is influenced by its true value. |
| 3.4. If Y/PY/NI to 3.3: Is it likely that missingness in the outcome depended on its true value? | PY |  |
| Authors risk-of-bias judgement of domain: | **HIGH** | |
| *Domain 4: Risk of bias in measurement of the outcome* | | |
| **Signaling Question** | **Response** | **Description** |
| 4.1. Was the method of measuring the outcome inappropriate? | N | “Primary and Secondary Outcomes. The primary outcome was incidence of depression relapse and time-to-relapse over 12 months of follow-up. DSM-IV-TR criteria for a major depressive episode (APA, 2000) were assessed by the mood disorders module of the SCID-IV (First et al, 1994).” [Measures, p15]  Using the SCID-IV to judge relapse is considered suitable for this outcome, by the review team. |
| 4.2. Could measurement or ascertainment of the outcome have differed between intervention groups? | N | Were conducted at the 12-month follow-up. |
| 4.3. If N/PN/NI to 4.1 and 4.2: Were outcome assessors aware of the intervention received by study participants? | PN | “Clinical interviews were conducted by a research assistant blind to group assignment.” [Measures, p15] |
| 4.4. If Y/PY/NI to 4.3: Could assessment of the outcome have been influenced by knowledge of intervention received? | n/a | n/a |
| 4.5. If Y/PY/NI to 4.4: Is it likely that assessment of the outcome was influenced by knowledge of intervention received? | n/a |  |
| Authors risk-of-bias judgement of domain: | **LOW** | |
| *Domain 5: Risk of bias in selection of the reported result* | | |
| **Signaling Question** | **Response** | **Description** |
| 5.1. Were the data that produced this result analysed in accordance with a pre-specified analysis plan that was finalized before unblinded outcome data were available for analysis? | NI | In an author’s note online we found: “Because the trial was conceived prior to 2008, pre-registration was not possible. However, the trial was registered on ClinicalTrials.gov after data collection and analysis (Title: “Decentering and Relapse/Recurrence in MBCT for Depression in Adults”, Identifier: NCT05111665).” No detailed pre-specified intentions are available to the review authors. Thus, planned outcome measures and analyses cannot be compared to those presented in the reviewed trials/articles. |
| Is the numerical result being assessed likely to have been selected, on the basis of the results, from... | | |
| 5.2. ...multiple eligible outcome measurements (e.g. scales, definitions, time points) within the outcome domain? | NI | Pre-specified definitions of outcomes are not available or reported in sufficient detail to facilitate the assessment and reach a valid judgement. |
| 5.3. ...multiple eligible analyses of the data? | NI | Analysis intentions are not available or reported in sufficient detail to facilitate the assessment and reach a valid judgement. |
| Authors risk-of-bias judgement of domain: | **SOME CONCERNS** | |
| **Study: Teasdale et al. (2010)** | | |
| *Domain 1: Randomization Process* | | |
| **Signaling Question** | **Response** | **Description** |
| 1.1. Was the allocation sequence random? | NI | “Randomization involved treatment sites faxing patient initials, date of birth, gender, date of assessment, and details of number and recency of previous episodes of depression to a central independent allocator. Information was sent for groups of eligible patients at a time. The central allocator randomly allocated patients to treatment condition, gave each a study number, and faxed the allocations and study numbers back to treatment sites.”  [Method, p617] |
| 1.2. Was the allocation sequence concealed until participants were enrolled and assigned to interventions? | Y |  |
| 1.3. Did baseline differences between intervention groups suggest a problem with the randomization process? | N | “Baseline characteristics of the intent-to-treat sample are given in Table 1. The TAU and MBCT treatment groups were closely similar on each of the baseline variables, with the exception of age.”  [Results, p619]  No significant baseline imbalances apparent from the manuscript or Table 1, that signal randomization issues. |
| Authors risk-of-bias judgement of domain: | **LOW** | |
| *Domain 2: Deviations from intended interventions* | | |
| **Signaling Question** | **Response** | **Description** |
| 2.1. Were participants aware of their assigned intervention during the trial? | Y | Awareness of the assigned interventions is inherent to the design of the trial and the intervention examined, given that participants could be allocated to MBCT + TAU vs TAU only. |
| 2.2. Were carers and people delivering the interventions aware of participants' assigned intervention during the trial? | Y |  |
| 2.3. If Y/PY/NI to 2.1 or 2.2: Were there deviations from the intended intervention that arose because of the trial context? | NI | “TAU The treatment for depression actually received by patients in the TAU condition was monitored at the bimonthly assessment interviews over the follow-up period and is summarized in Table 2. The corresponding data for patients in the MBCT condition are also shown for comparison. There were no statistically significant differences between the TAU and MBCT conditions for any of these measures of treatment received (all ps > .10).” [Results, TAU, p619]  However, TAU is considered part of the trial context. Thus, not enough information available for the reviewers to make an adequate judgement about any intervention outside of trial context that were sought. |
| 2.4. If Y/PY to 2.3: Were these deviations likely to have affected the outcome? | n/a | n/a |
| 2.5. If Y/PY/NI to 2.4: Were these deviations from intended intervention balanced between groups? | n/a | n/a |
| 2.6. Was an appropriate analysis used to estimate the effect of assignment to intervention? | Y | Analyses were conducted on the ITT principle, as well as per-protocol.  “Results were analyzed separately for an intent-to-treat sample (n — 145), comprising all of the patients included in the random allocation, and a per-protocol sample (n ~ 132), comprising (a) all of the patients allocated to the TAU condition (n = 69) and (b) those patients allocated to MBCT who received a predetermined "minimum effective dose" of MBCT (at least four of the eight weekly MBCT sessions; n = 63). The results from these two samples are complementary: …” [Results, p618] |
| 2.7. If N/PN/NI to 2.6: Was there potential for a substantial impact (on the result) of the failure to analyse participants in the group to which they were randomized? | n/a | n/a |
| Authors risk-of-bias judgement of domain: | **SOME CONCERNS** | |
| *Domain 3: Risk of bias due to missing outcome data* | | |
| **Signaling Question** | **Response** | **Description** |
| 3.1. Were data for this outcome available for all, or nearly all, participants randomized? | Y | In total, 145 patients were randomized.  “At three treatment sites, 145 patients, currently in remission or recovery from major depression at the time of the baseline assessment, were randomized to continue with TAU or, additionally, to receive MBCT training.” [Method, p617]  “Complete data on relapse or recurrence were available for 137 (95%) of the 145 patients in the intent-to-treat sample and 128 (97%) of the 132 patients in the per-protocol sample; data were incomplete for 3 TAU patients, 4 "insufficient treatment" MBCT patients, and 1 "adequate treatment" MBCT patient.”  [Results, p619] |
| 3.2. If N/PN/NI to 3.1: Is there evidence that the result was not biased by missing outcome data? | n/a | n/a |
| 3.3. If N/PN to 3.2: Could missingness in the outcome depend on its true value? | n/a | n/a |
| 3.4. If Y/PY/NI to 3.3: Is it likely that missingness in the outcome depended on its true value? | n/a |  |
| Authors risk-of-bias judgement of domain: | **LOW** | |
| *Domain 4: Risk of bias in measurement of the outcome* | | |
| **Signaling Question** | **Response** | **Description** |
| 4.1. Was the method of measuring the outcome inappropriate? | N | “Relapse/recurrence. The primary-outcome variable was the occurrence of relapse or recurrence meeting DSM-IH-R criteria for major depressive episode (American Psychiatric Association, 1987), as assessed by the Structured Clinical Interview for DSM-IH-R (SCID; Spitzer, Williams, Gibbon, & First, 1992)” [Method, p618]  Using the SCID to judge relapse is considered suitable for this outcome, by the review team. |
| 4.2. Could measurement or ascertainment of the outcome have differed between intervention groups? | PN | “… administered at bimonthly assessments through the follow-up period and covering the period from the previous assessment.” |
| 4.3. If N/PN/NI to 4.1 and 4.2: Were outcome assessors aware of the intervention received by study participants? | PN | “To maintain blindness of assessors to treatment condition, we instructed patients not to reveal whether they were receiving MBCT or any details that might prejudice blindness. Nonetheless, assessors occasionally became aware of a patient's treatment condition. To overcome such occasional unwinding, and to examine interrater reliability, interviews were audiotaped and all 133 occasions on which patients met the screening criteria for major depression were evaluated by an independent, blind, experienced research psychiatrist (any information potentially revealing patients' treatment allocation was excluded from the taped interview presented to the blind assessor).” |
| 4.4. If Y/PY/NI to 4.3: Could assessment of the outcome have been influenced by knowledge of intervention received? | n/a | n/a |
| 4.5. If Y/PY/NI to 4.4: Is it likely that assessment of the outcome was influenced by knowledge of intervention received? | n/a |  |
| Authors risk-of-bias judgement of domain: | **LOW** | |
| *Domain 5: Risk of bias in selection of the reported result* | | |
| **Signaling Question** | **Response** | **Description** |
| 5.1. Were the data that produced this result analysed in accordance with a pre-specified analysis plan that was finalized before unblinded outcome data were available for analysis? | NI | No detailed pre-specified intentions available to the review authors. Thus, planned outcome measures and analyses cannot be compared to those presented in the reviewed trials/articles. |
| Is the numerical result being assessed likely to have been selected, on the basis of the results, from... | | |
| 5.2. ...multiple eligible outcome measurements (e.g. scales, definitions, time points) within the outcome domain? | NI | Pre-specified definitions of outcomes are not available or reported in sufficient detail to facilitate the assessment and reach a valid judgement. |
| 5.3. ...multiple eligible analyses of the data? | NI | Analysis intentions are not available or reported in sufficient detail to facilitate the assessment and reach a valid judgement. |
| Authors risk-of-bias judgement of domain: | **SOME CONCERNS** | |
| **Study: Williams et al. (2014)** | | |
| *Domain 1: Randomization Process* | | |
| **Signaling Question** | **Response** | **Description** |
| 1.1. Was the allocation sequence random? | Y | “Randomization was by e-mail to the North Wales Organisation for Randomised Trials in Health, which used dynamic allocation (Russell, Hoare, Whitaker, Whitaker, & Russell, 2011) to stratify by two variables in addition to site and recruitment cohort:…”  [Method, p277]  “Participants were informed of the outcome of randomization by letter and also by e-mail or telephone if desired. Once they had been notified of their allocation, ….”  [Method, p277] |
| 1.2. Was the allocation sequence concealed until participants were enrolled and assigned to interventions? | Y |  |
| 1.3. Did baseline differences between intervention groups suggest a problem with the randomization process? | N | “Table 1 shows the baseline characteristics of those who provided follow-up data by treatment group. The 19 participants lost to follow-up were significantly younger than those who provided follow-up data, by 5.6 years (95% CI [1.5, 9.7]). There were no other significant differences between the groups.”  [Results, p279]  No baseline imbalances apparent that could indicate problems with the randomization procedures employed. |
| Authors risk-of-bias judgement of domain: | **LOW** | |
| *Domain 2: Deviations from intended interventions* | | |
| **Signaling Question** | **Response** | **Description** |
| 2.1. Were participants aware of their assigned intervention during the trial? | y | Awareness of the assigned interventions is inherent to the design of the trial and the intervention examined, given that participants could be allocated to MBCT, CPE, or TAU. |
| 2.2. Were carers and people delivering the interventions aware of participants' assigned intervention during the trial? | Y |  |
| 2.3. If Y/PY/NI to 2.1 or 2.2: Were there deviations from the intended intervention that arose because of the trial context? | PN | “… records were kept of the treatment sought at each assessment point. These data indicated that 21% of patients in TAU received one or more new antidepressant prescriptions during the follow-up phase, in comparison to 18% of participants in MBCT and 13% of those in CPE. Similarly, 11% of those in TAU saw a psychiatrist or community psychiatric nurse regarding their depression at least once during the follow-up phase in comparison to 10% of those allocated to MBCT and 9% of those allocated to CPE. Finally, 21% of those allocated to TAU saw a counselor, psychologist, or psychotherapist at least once during the follow-up phase, compared with 18% of those allocated to MBCT and 12% of those allocated to CPE. There were no significant differences between groups in pharmacological or psychiatric/psychological treatment received. All participants were offered their treatment of choice once data collection was over, regardless of whether they were in the MBCT, CPE, or TAU arm initially.” [Method, p278]  “Nineteen (7%) left the trial before any follow-up data could be collected: nine from MBCT, seven from CPE, and three from TAU. Of these, two explicitly withdrew from the trial, one moved away, and the remaining 16 repeatedly failed to attend scheduled follow-up appointments.” [Results, 279] |
| 2.4. If Y/PY to 2.3: Were these deviations likely to have affected the outcome? | n/a | n/a |
| 2.5. If Y/PY/NI to 2.4: Were these deviations from intended intervention balanced between groups? | n/a | n/a |
| 2.6. Was an appropriate analysis used to estimate the effect of assignment to intervention? | Y | “Data were analyzed by treatment allocated.”  [Method, p279] |
| 2.7. If N/PN/NI to 2.6: Was there potential for a substantial impact (on the result) of the failure to analyse participants in the group to which they were randomized? | n/a | n/a |
| Authors risk-of-bias judgement of domain: | **LOW** | |
| *Domain 3: Risk of bias due to missing outcome data* | | |
| **Signaling Question** | **Response** | **Description** |
| 3.1. Were data for this outcome available for all, or nearly all, participants randomized? | Y | In total, 274 participants were randomly allocated to either one of the conditions.  “Thus, follow-up data were available for 255 participants: 99 receiving MBCT, 103 CPE, and 53 TAU.” [Results, 279]  Thus, outcome data is available for 93.07% of participants. |
| 3.2. If N/PN/NI to 3.1: Is there evidence that the result was not biased by missing outcome data? | n/a | n/a |
| 3.3. If N/PN to 3.2: Could missingness in the outcome depend on its true value? | n/a | n/a |
| 3.4. If Y/PY/NI to 3.3: Is it likely that missingness in the outcome depended on its true value? | n/a |  |
| Authors risk-of-bias judgement of domain: | **LOW** | |
| *Domain 4: Risk of bias in measurement of the outcome* | | |
| **Signaling Question** | **Response** | **Description** |
| 4.1. Was the method of measuring the outcome inappropriate? | N | “The primary outcome was time until relapse to major depression, defined as meeting relevant SCID criteria for at least 2 weeks since the previous assessment. Participants were asked to date the onset of their episode as accurately as possible. Where they could not give a precise date of onset, we used an algorithm to approximate the date of onset for derivation of “a days to relapse” variable.1 We recorded all SCIDs and recruited two independent psychiatrists to reassess a sample of 91 follow-up interviews. Psychiatrists received only the audio-recording of the clinical interview. Cases for independent review were selected by a researcher blind to all participant details, who had not listened to the audiorecordings, with the aim of including approximately two relapses per assessor, per cohort, per assessment wave (posttreatment, 3, 6, 9, 12 months). Once each relapse interview had been identified, the next available case within a cohort and without a relapse at the assessment in question was selected as a control. Interrater reliability between the original assessor and an independent psychiatrist was 0.74, 95% CI [0.60, 0.87], with 87% agreement on whether relapse had occurred.” [Method, p279]  Using the SCID to judge relapse is considered suitable for this outcome, by the review team. |
| 4.2. Could measurement or ascertainment of the outcome have differed between intervention groups? | N | “… using fully trained assessors blind to treatment allocation before randomization; immediately after treatment (or at the equivalent point for the TAU group); and then at 3, 6, 9, and 12 months after treatment or equivalent.” [Method, p278] |
| 4.3. If N/PN/NI to 4.1 and 4.2: Were outcome assessors aware of the intervention received by study participants? | N | “… using fully trained assessors blind to treatment allocation before randomization; immediately after treatment (or at the equivalent point for the TAU group); and then at 3, 6, 9, and 12 months after treatment or equivalent. We strove to maintain blinding: For example, we assessed participants in different buildings from those in which treatment took place and checked assessor blindness at the end of every assessment session. On the rare occasions on which an assessor became unblinded due to overt disclosure by a participant, we used an alternative assessor for all subsequent assessments.” [Method, p278] |
| 4.4. If Y/PY/NI to 4.3: Could assessment of the outcome have been influenced by knowledge of intervention received? | n/a | n/a |
| 4.5. If Y/PY/NI to 4.4: Is it likely that assessment of the outcome was influenced by knowledge of intervention received? | n/a |  |
| Authors risk-of-bias judgement of domain: | **LOW** | |
| *Domain 5: Risk of bias in selection of the reported result* | | |
| **Signaling Question** | **Response** | **Description** |
| 5.1. Were the data that produced this result analysed in accordance with a pre-specified analysis plan that was finalized before unblinded outcome data were available for analysis? | Y | Trial registry (ISRCTN97185214) and published protocol were consulted. The trial register was pre-registered on 04/01/2010.  Williams, J. M. G., Russell, I. T., Crane, C., Russell, D., Whitaker, C. J., Duggan, D. S., ... & Silverton, S. (2010). Staying well after depression: trial design and protocol. BMC psychiatry, 10, 1-10.  Researchers did indeed pre-specify their intentions in sufficient detail, and they match the published manuscript. |
| Is the numerical result being assessed likely to have been selected, on the basis of the results, from... | | |
| 5.2. ...multiple eligible outcome measurements (e.g. scales, definitions, time points) within the outcome domain? | PN | All eligible reported results for the outcome measurement seem to correspond to all intended measurements. |
| 5.3. ...multiple eligible analyses of the data? | PN | All eligible reported results for the outcome measurement seem to correspond to all intended analyses. |
| Authors risk-of-bias judgement of domain: | **LOW** | |

# Supplementary Appendix 6. Overview of follow-up times in included studies and censoring of data conducted.

| **Study** | **Maximum follow-up available** | **Included in censoring at 12 months** |
| --- | --- | --- |
| Bockting et al. (2005) | 24 | Yes |
| Bockting et al. (2018) | 24 | Yes |
| Bondolfi et al. (2010) | 15 | Yes |
| De Jonge et al. (2019) | 15 | Yes |
| Godfrin et al. (2010) | 26 | Yes |
| Hitchcock et al. (2021) | 12 | Yes |
| Holländare et al. (2013) | 26 | Yes |
| Huijbers et al. (2015) | 15 | Yes |
| Jarrett et al. (2001) | 24 | Yes |
| Jarrett et al. (2013) | 32 | Yes |
| Klein et al. (2004) | 12 | Yes |
| Klein et al. (2018) | 23 | Yes |
| Teasdale et al. (2000) | 14 | Yes |
| Ma et al. (2004) | 14 | Yes |
| Moore et al. (2022) | 12 | Yes |
| Williams et al. (2014) | 14 | Yes |

# Supplementary Appendix 7. Studies with IPD not provided and reasons given.

| **Study** | **Agreed to participate** | **Data shared** | **Status** |
| --- | --- | --- | --- |
| Brakemeier 2014 | Yes | No | Ethical/data sharing regulations: Unable to take part due to data transfer regulations and patient consent restrictions. |
| Frank et al. (1990) | No | No | Data lost/unavailable: Access issues, resource and staff moved; the data was no longer available. |
| Katon et al. (2001) | No | No | Data lost/unavailable: Lab closed. |
| Meadows et al. (2014) | No | No | Ethical/data sharing regulations: Data was unavailable due to ethics regulations. |
| Paykel et al. (1999, 2005) | No | No | Reason unclear: Unable to provide data (reason unclear/no further rationale provided). |
| Perlis et al. (2002) | No | No | Reason unclear: Unable to provide data (reason unclear/no further rationale provided). |
| Petersen et al. (2010) | No | No | Time constraints: Unable to provide data (reason unclear/no further rationale provided). |
| Sheets et al. (2013) | No | No | Data lost/unavailable: Data not available at this point. |
| Stangier et al. (2013) | Yes | No | Time constraints: Data transfer agreement (DTA) not signed in time. |
| Teismann et al. (2014) | No | No | Reason unclear: Unable to share data due to other reasons. |

| Supplementary Table 1. PRISMA-IPD Checklist. | | | |
| --- | --- | --- | --- |
| **PRISMA-IPD**  **Section/topic** | **Item No** | **Checklist item** | **Reported on page** |
| *Title* | | | |
| Title | 1 | Identify the report as a systematic review and meta-analysis of individual participant data. | p. 1 |
| *Abstract* | | | |
| Structured summary | 2 | Provide a structured summary including as applicable: | p. 2 |
|  |  | *Background*: state research question and main objectives, with information on participants, interventions, comparators and outcomes. |  |
|  |  | *Methods*: report eligibility criteria; data sources including dates of last bibliographic search or elicitation, noting that IPD were sought; methods of assessing risk of bias. |  |
|  |  | *Results*: provide number and type of studies and participants identified and number (%) obtained; summary effect estimates for main outcomes (benefits and harms) with confidence intervals and measures of statistical heterogeneity. Describe the direction and size of summary effects in terms meaningful to those who would put findings into practice. |  |
|  |  | *Discussion***:** state main strengths and limitations of the evidence, general interpretation of the results and any important implications. |  |
|  |  | *Other***:** report primary funding source, registration number and registry name for the systematic review and IPD meta-analysis. |  |
| *Introduction* | | | |
| Rationale | 3 | Describe the rationale for the review in the context of what is already known. | p. 3-5 |
| Objectives | 4 | Provide an explicit statement of the questions being addressed with reference, as applicable, to participants, interventions, comparisons, outcomes and study design (PICOS). Include any hypotheses that relate to particular types of participant-level subgroups. | p. 5 |
| *Methods* | | | |
| Protocol and registration | 5 | Indicate if a protocol exists and where it can be accessed. If available, provide registration information including registration number and registry name. Provide publication details, if applicable. | p. 5 |
| Eligibility criteria | 6 | Specify inclusion and exclusion criteria including those relating to participants, interventions, comparisons, outcomes, study design and characteristics (e.g. years when conducted, required minimum follow-up). Note whether these were applied at the study or individual level i.e. whether eligible participants were included (and ineligible participants excluded) from a study that included a wider population than specified by the review inclusion criteria. The rationale for criteria should be stated. | p. 5 |
| Identifying studies - information sources | 7 | Describe all methods of identifying published and unpublished studies including, as applicable: which bibliographic databases were searched with dates of coverage; details of any hand searching including of conference proceedings; use of study registers and agency or company databases; contact with the original research team and experts in the field; open adverts and surveys. Give the date of last search or elicitation. | p. 5 |
| Identifying studies - search | 8 | Present the full electronic search strategy for at least one database, including any limits used, such that it could be repeated. | p. 5 |
| Study selection processes | 9 | State the process for determining which studies were eligible for inclusion. | p. 5 |
| Data collection processes | 10 | Describe how IPD were requested, collected and managed, including any processes for querying and confirming data with investigators. If IPD were not sought from any eligible study, the reason for this should be stated (for each such study). | p. 5-6 |
|  |  | If applicable, describe how any studies for which IPD were not available were dealt with. This should include whether, how and what aggregate data were sought or extracted from study reports and publications (such as extracting data independently in duplicate) and any processes for obtaining and confirming these data with investigators. |  |
| Data items | 11 | Describe how the information and variables to be collected were chosen. List and define all study level and participant level data that were sought, including baseline and follow-up information. If applicable, describe methods of standardising or translating variables within the IPD datasets to ensure common scales or measurements across studies. | p. 5-6 |
| IPD integrity | A1 | Describe what aspects of IPD were subject to data checking (such as sequence generation, data consistency and completeness, baseline imbalance) and how this was done. | p. 5-6 |
| Risk of bias assessment in individual studies. | 12 | Describe methods used to assess risk of bias in the individual studies and whether this was applied separately for each outcome. If applicable, describe how findings of IPD checking were used to inform the assessment. Report if and how risk of bias assessment was used in any data synthesis. | p. 6 |
| Specification of outcomes and effect measures | 13 | State all treatment comparisons of interests. State all outcomes addressed and define them in detail. State whether they were pre-specified for the review and, if applicable, whether they were primary/main or secondary/additional outcomes. Give the principal measures of effect (such as risk ratio, hazard ratio, difference in means) used for each outcome. | p. 5-8 |
| Synthesis methods | 14 | Describe the meta-analysis methods used to synthesise IPD. Specify any statistical methods and models used. Issues should include (but are not restricted to):   - Use of a one-stage or two-stage approach. - How effect estimates were generated separately within each study and combined across studies (where applicable). - Specification of one-stage models (where applicable) including how clustering of patients within studies was accounted for. - Use of fixed or random effects models and any other model assumptions, such as proportional hazards. - How (summary) survival curves were generated (where applicable). - Methods for quantifying statistical heterogeneity (such as I^2^ and τ^2^). - How studies providing IPD and not providing IPD were analysed together (where applicable). - How missing data within the IPD were dealt with (where applicable). | p. 6-8 |
| Exploration of variation in effects | A2 | If applicable, describe any methods used to explore variation in effects by study or participant level characteristics (such as estimation of interactions between effect and covariates). State all participant-level characteristics that were analysed as potential effect modifiers, and whether these were pre-specified. | p. 6-8 |
| Risk of bias across studies | 15 | Specify any assessment of risk of bias relating to the accumulated body of evidence, including any pertaining to not obtaining IPD for particular studies, outcomes or other variables. | p. 6-8 |
| Additional analyses | 16 | Describe methods of any additional analyses, including sensitivity analyses. State which of these were pre-specified. | p. 6-8 |
| *Results* | | | |
| Study selection and IPD obtained | 17 | Give numbers of studies screened, assessed for eligibility, and included in the systematic review with reasons for exclusions at each stage. Indicate the number of studies and participants for which IPD were sought and for which IPD were obtained. For those studies where IPD were not available, give the numbers of studies and participants for which aggregate data were available. Report reasons for non-availability of IPD. Include a flow diagram. | p. 8-9  Figure 1  sTable 4  sAppx 7 |
| Study characteristics | 18 | For each study, present information on key study and participant characteristics (such as description of interventions, numbers of participants, demographic data, unavailability of outcomes, funding source, and if applicable duration of follow-up). Provide (main) citations for each study. Where applicable, also report similar study characteristics for any studies not providing IPD. | p. 8-9  sTable 4  sAppx 6 |
| IPD integrity | A3 | Report any important issues identified in checking IPD or state that there were none. | sAppx 3 |
| Risk of bias within studies | 19 | Present data on risk of bias assessments. If applicable, describe whether data checking led to the up-weighting or down-weighting of these assessments. Consider how any potential bias impacts on the robustness of meta-analysis conclusions. | p. 11-12  sFigure 5  sAppx 4-5 |
| Results of individual studies | 20 | For each comparison and for each main outcome (benefit or harm), for each individual study report the number of eligible participants for which data were obtained and show simple summary data for each intervention group (including, where applicable, the number of events), effect estimates and confidence intervals. These may be tabulated or included on a forest plot. | p. 9-11  Figure 2  Table 2-3  sFigure 1  sTable 5-8 |
| Results of syntheses | 21 | Present summary effects for each meta-analysis undertaken, including confidence intervals and measures of statistical heterogeneity. State whether the analysis was pre-specified, and report the numbers of studies and participants and, where applicable, the number of events on which it is based. | p. 9-11  Figure 2  Table 2-3  sFigure 1  sTable 5-8 |
|  |  | When exploring variation in effects due to patient or study characteristics, present summary interaction estimates for each characteristic examined, including confidence intervals and measures of statistical heterogeneity. State whether the analysis was pre-specified. State whether any interaction is consistent across trials. |  |
|  |  | Provide a description of the direction and size of effect in terms meaningful to those who would put findings into practice. |  |
| Risk of bias across studies | 22 | Present results of any assessment of risk of bias relating to the accumulated body of evidence, including any pertaining to the availability and representativeness of available studies, outcomes or other variables. | p. 11-12  sFigure 5  sAppx 4-5 |
| Additional analyses | 23 | Give results of any additional analyses (e.g. sensitivity analyses). If applicable, this should also include any analyses that incorporate aggregate data for studies that do not have IPD. If applicable, summarise the main meta-analysis results following the inclusion or exclusion of studies for which IPD were not available. | p. 9-11  Figure 2  Table 2-3  sFigure 1  sTable 5-8 |
| *Discussion* | | | |
| Summary of evidence | 24 | Summarise the main findings, including the strength of evidence for each main outcome. | p. 12-16 |
| Strengths and limitations | 25 | Discuss any important strengths and limitations of the evidence including the benefits of access to IPD and any limitations arising from IPD that were not available. | p. 14-15 |
| Conclusions | 26 | Provide a general interpretation of the findings in the context of other evidence. | p. 12-16 |
| Implications | A4 | Consider relevance to key groups (such as policy makers, service providers and service users). Consider implications for future research. | p. 12-16 |
| *Funding* | | | |
| Funding | 27 | Describe sources of funding and other support (such as supply of IPD), and the role in the systematic review of those providing such support. | p. 17 |

| Supplementary Table 2. Depression symptom severity transformation table used for values available. | | | | |
| --- | --- | --- | --- | --- |
| HDRS-17 | BDI-I ^a^ | BDI-II ^a^ | HDRS-24 ^b^ | IDS-C ^b^ |
| 0 | 0 | 0 | 0-1 | 0-3 |
| 1 | 1 | 1 |  |  |
| 1.5 * |  |  | 2 * | 4-5 * |
| 2 | 2 | 2 |  |  |
| 3 | 3 | 3 | 3-4 | 6 |
| 3.5 * |  | 4 * |  |  |
| 4 | 4 | 5 | 5 | 7-8 |
| 5 | 5 | 6 |  |  |
| 5.5 * |  | 7 * | 6-7 * | 9-11 * |
| 6 | 6 | 8 |  |  |
| 7 | 7 | 9 | 8-9 | 12-13 |
| 7.5 * |  | 10 * |  |  |
| 8 | 8 | 11 | 10-11 | 14-16 |
| 9 | 9 | 12 |  |  |
| 9.5 * |  |  | 12 * | 17-18 |
| 10 | 10 | 13 |  |  |
| 10.5 * | 11 * | 14 * |  |  |
| 11 | 12 | 15 | 13-14 | 19-21 |
| 12 | 13 | 16 | 15 | 22-23 |
| 12.5 * | 14 * | 17 * |  |  |
| 13 | 15 | 18 |  | 24-25 |
| 13.5 * |  | 19 * |  |  |
| 14 | 16 | 20 |  |  |
| 15 | 17 | 21 |  |  |
| 15.5 * | 18 * | 22 * |  |  |
| 16 | 19 | 23 |  | 29-30 |
| 16.5 * | 20 * | 24 |  |  |
| 17 | 21 | 25 |  | 32-33 |
| 17.5 * | 22 * | 26 * |  |  |
| 18 | 23 | 27 |  |  |
| 18.5 * | 24 * | 28-29 * |  | 34-36 * |
| 19 | 25 | 30 |  |  |
| 19.5 * | 26 * | 31 * |  |  |
| 20 | 27 | 32 |  |  |
| 20.5 * |  | 33 * |  |  |
| 21 | 28 | 34 |  |  |
| 21.5 * | 29 * | 35 |  |  |
| 22 | 30 | 36 |  |  |
| 22.5 * |  | 37 * |  |  |
| 23 | 31 | 38 |  |  |
| 23.5 * |  | 39 * |  |  |
| 24 | 33 | 40 |  |  |
| 24.5 * |  | 41 * |  |  |
| 25 | 34 | 42 |  |  |
| 25.5 * | 35 * | 43 * |  |  |
| 26 | 36 | 44 |  |  |
| 27 | 38 | 45 |  |  |
| 28 | 39 | 47 |  |  |
| 28.5 * | 40 * | 48 * |  |  |
| 29 | 41 | 49 |  |  |
| 30 | 42 | 50 |  |  |
| * Calculated using the median of the two adjacent numbers.  ^a^ Using Furukawa TA et al (2020). *Epidemiology and Psychiatric Sciences*.  ^b^ Using Rush AJ et al (2003). *Biological Psychiatry*. | | | | |

| Supplementary Table 3. Sensitivity analysis excluding patients with HDRS-17 scores of 16 and above, on the primary outcome of time-to-relapse at 12 months. | | | | |
| --- | --- | --- | --- | --- |
| **Comparisons & Models** | **HR (95% CI)** | ***p*** | ***n* participants (relapsed)** | ***k* studies** |
| *Psychological interventions vs. non-psychological control conditions* | | | | |
| 2-stage random effects model | 0.73 (0.53–0.99) | .042 | 542 (233) | 14 ^a^ |
| 2-stage fixed effects model | 0.73 (0.60–0.96) | .023 | 542 (233) | 14 ^a^ |
| *Psychological interventions plus TAU vs. TAU only* | | | | |
| 2-stage random effects model | 0.60 (0.45–0.78) | .003 | 312 (172) | 8 |
| 2-stage fixed effects model | 0.60 (0.43–0.84) | .003 | 312 (172) | 8 |
| ^a^ Jarrett et al. (2001) and Klein et al. (2004) were excluded from the two-stage models, as there were too few relapses observed. | | | | |

| Supplementary Table 4. Characteristics of included studies. | | | | | | | | | | | | | | | |  |
| --- | --- | --- | --- | --- | --- | --- | --- | --- | --- | --- | --- | --- | --- | --- | --- | --- |
| **Study** | **Country** | **Intervention** | **Control Group** | **Total FU (wk)** | **MDE Required at Inclusion** | **Symptom Severity Inclusion Criteria** | **RoB** ^a^ | **Partial / Full Remission** | **Relapse 12 mo / Partial Remitters** | **Relapse** | **Depressive Symptom Severity Outcome** | **Quality of Life Outcome** | **Mean HDRS-17^b^**  **Partial Remitters** |  |  |  |
| Bockting et al. (2005) | NL | *PCT + TAU*: 8 weekly 2h cognitive therapy group sessions + TAU. ADM = 52%; PT = 29%. | *TAU*: Standard treatment, e.g. ADM, psychotherapy or no treatment, no restrictions. ADM = 50%; PT = 33%. | 1040 | 2 | HDRS-17 <10 | +/- | 30/142 | 17/30 | SCID-I |  |  | 8.33 |  |  |  |
| Bockting et al. (2018) | NL | *(1) PCT + ADM*: 8 weekly 2h cognitive therapy group or individual sessions + ADM. ADM = 100%; PT = 31%.  *(2) PCT + tapering ^c^*: 8 weekly 2h cognitive therapy group or individual sessions + supervised tapering. | *ADM*: ADM continuation at min. required adequate doses (≥20 mg fluoxetine equivalent), guided by GPs or psychiatrists. ADM = 100%; PT = 31%. | 104 | 2 | HDRS-17 ≤10 | - | 24/123 | 13/24 | SCID-I | HDRS-17 | EQ-5D-5L | 8.88 |  |  |  |
| Bondolfi et al. (2010) | CH | *MBCT + TAU*: 8 weekly 2h group training sessions + TAU. ADM = 36%; PT = 46%. | *TAU*: Care from GP or other sources as participants would normally seek. ADM = 31%; PT = 55%. | 56 | 3 | MADRS ≤13 | +/- | 21/34 | 10/21 | SCID-I | MADRS |  | 14.10 |  |  |  |
| Godfrin et al. (2010) | BE | *MBCT + TAU*: 8 weekly 2¾h group training sessions + TAU. ADM = 73%; PT = 27%. | *TAU*: Waiting-list, but non-trial treatment allowed and monitored across follow-up. ADM = 61%; PT = 13%. | 56 | 3 | HDRS-17 <14 | - | 64/22 | 37/64 | SCID-I | BDI-II |  | 16.42 |  |  |  |
| Hitchcock et al. (2021) | UK | *MemFlex*: 8 sessions of self-guided workbook exercises over 4 wk to improve memory skills, with 30-45 min face-to-face session introduction. ADM = 73%; PT = 30%. | *PE*: 8 sessions of workbook psychoeducation, with 30-45 min face-to-face introduction and final session focused on relapse prevention plan. ADM = 51%; PT = 22%. | 56 | n.s. | n.s. | + | 38/18 | 31/38 | LIFE | BDI-II |  | 15.08 |  |  |  |
| Holländare et al. (2013) | SE | *CBT*: 16 modules over 10 wk guided self-help CBT. Unrestricted email communication with personal therapist: behavioral activation, cognitive restructuring, preventive strategies and skills training. ADM = 43%; PT = 0%. | *TAU*: Limited non-specific general support contact with therapist, for 10 wk. ADM = 57%; PT = 0%. | 104 | 1 | MADRS ≤19 | - | 72/12 | 16/72 | SCID-I | BDI-II | WHOQOL-BREF | 13.42 |  |  |  |
| Huijbers et al. (2015) | NL | *MBCT + ADM*: 8 weekly 2½h group sessions and 1 day of silent practice + ADM. ADM = 85%; PT = unclear. | *ADM*: Maintaining or reinstate an adequate dose of ADM, managing side effects, guided by psychiatrists. ADM = 80%; PT = unclear. | 60 | 3 | n.s. | - | 25/43 | 10/25 | SCID-I | IDS-C | EQ-5D-VAS | 12.18 |  |  |  |
| Jarrett et al. (2001) | USA | *C-CT*: 10 sessions of 60-90 min of manualized therapy, to prevent relapse and recurrence, review strategies associated with effective symptom reduction, maintain skills acquired during acute-phase CT, and develop coping strategies. First 4 sessions biweekly, last 6 sessions monthly. ADM = 0%; PT = 0%. | *Eval only / TAU ^d^*: 10 assessments at same frequency as C-CT for 8 mo; no CT or psychosocial interventions before relapse. ADM = 0%; PT = 0%. | 32 | 2 | HDRS-17 ≤9 | - | 11/73 | 1/11 | SCID-I | HDRS-17 | SAS-SR | 8.36 |  |  |  |
| Jarrett et al. (2013) | USA | *C-CT*: 10 sessions of 60 min manualized individual therapy, aimed to prevent relapse and promote remission/recovery. First 4 sessions biweekly, last 6 sessions monthly. ADM = 0%; PT = unclear. | *(1) ADM*: Fluoxetine, guided by pharmacotherapists, incl. clinical management, at same frequency as C-CT. ADM = 100%; PT = unclear.  *(2) P-ADM ^c^*: pill placebo, guided by pharmacotherapists, incl. clinical management, at same frequency as C-CT. ADM = 0%; PT = unclear. | 128 | 1 | HDRS-17 ≤12 | + | 96/76 | 21/96 | LIFE | HDRS-17 | SAS-SR | 10.14 |  |  |  |
| de Jonge et al. (2019) | NL | *PCT + TAU*: 8 weekly 2h individual cognitive therapy sessions + TAU. ADM = 23%; PT = unclear. | *TAU*: Usual primary/specialty care after CBT, consists of no care at all; if provided, it is usually ADM. ADM = 34%; PT = unclear. | 60 | 2 | HDRS-17 <14 | +/- | 45/150 | 17/45 | SCID-I | HDRS-17 | EQ-5D-3L | 9.67 |  |  |  |
| Klein et al. (2004) | USA | *CBASP*: 1 session per 4 wk for a total of up to 13 sessions. Integrative, manualized, psychotherapy to treat chronic depression. All psychotropic medication and non-protocol psychotherapy prohibited. ADM = 0%; PT = 0%. | *Eval only*: Meeting every 4 wk with evaluator. All psychotropic medication and non-protocol psychotherapy prohibited. ADM = 0%; PT = 0%. | 52 | n.s. | HDRS-24 ≤15 | - | 18/64 | 3/18 | SCID-I | HDRS-24 |  | 9.94 |  |  |  |
| Klein et al. (2018) | NL | *PCT + TAU*: 8 online modules based on PCT, targeting cognitive vulnerability factors to prevent relapse + TAU. ADM = 55%; PT = 40%. | *TAU*: No treatment, (after)care by GP, or (after)care in specialized mental health care. ADM = 51%; PT = 36%. | 96 | 2 | HDRS-17 ≤10 | - | 31/187 | 18/31 | SCID-I | HDRS-17 | EQ-5D-5L | 9.06 |  |  |  |
| Ma et al. (2004) | UK | *MBCT + TAU*: 8 weekly 2h, group training sessions with follow-up meetings at 1 and 6 months + TAU. ADM = 13-21%; PT = 13-21%. | *TAU*: Care from GP or other sources as participants would normally seek. ADM = 33-36%; PT = 19-30%. | 60 | 2 | HDRS-17 <10 | - | 56/17 | 28/56 | SCID-I | BDI |  | 14.35 |  |  |  |
| Moore et al. (2022) | CA | *MBCT*: 8 weekly 2h group training sessions. ADM = unclear; PT = unclear. | *(1) TAU*: Care from GP or other sources as participants would normally seek. ADM = unclear; PT = unclear.  *(2) RGT*: 8 weekly, 2 hour relaxation sessions. Active comparator controlling for non-specific group factors, based on Changeways Relaxation Programme. ADM = unclear; PT = unclear. | 52 | 3 | HDRS-17 <10 | - | 19/130 | 13/19 | SCID | HDRS-17 |  | 9.53 |  |  |  |
| Teasdale et al. (2000) | UK | *MBCT + TAU*: Orientation session, 8 weekly 2h group sessions + TAU. ADM = 45%; PT = 49%. | *TAU*: Care from GP or other sources as participants would normally seek. ADM = 40%; PT = 34%. | 60 | 2 | HDRS-17 <10 | - | 82/50 | 44/82 | SCID-I | BDI |  | 13.55 |  |  |  |
| Williams et al. (2014) | UK | *MBCT + TAU*: Individual interview, 8 weekly 2h group sessions, with follow-up classes at 6 wk and 6 mo posttreatment + TAU. ADM = 18%; PT = 18%. | *(1) TAU*: Interview with therapist and care from GP or other sources as participants would normally seek. ADM = 21%; PT = 21%.  *(2) CPE + TAU*: all elements of MBCT except experiential cultivation of mindfulness through meditation; same format of 8 weekly 2h sessions, with follow-up at 6 wk and 6 mo. ADM = 13%; PT = 12%. | 52 | 3 | n.s. | + | 73/182 | 34/73 | SCID-I | BDI-II |  | 12.82 |  |  |  |
| *Abbreviations*. ADM = antidepressant medication; BE = Belgium; BDI = Beck Depression Inventory version I or II; CA = Canada; CBASP = cognitive– behavioral analysis system of psychotherapy; CBT = Cognitive Behavioral Therapy; C-CT = Continuation Cognitive Therapy; CH = Switzerland; Eval only = Evaluation only; CPE = Cognitive Psychological Education EQ-5D = EuroQol five-dimensional questionnaire 3- or 5-level versions; FU = follow-up; HDRS = Hamilton Depression Rating Scale 17- or 24-item version; IDS-C = Inventory for Depressive Symptomatology-Clinician; LIFE = Longitudinal Interval Follow-up Evaluation; MADRS = Montgomery Åsberg Depression Rating Scale; MBCT = Mindfulness-Based Cognitive Therapy; MDE = major depressive episode; mo = months; n/a = not applicable; NL = The Netherlands; n.s. = not specified; P-ADM = placebo antidepressant medication; PCT = Preventive Cognitive Therapy; PE = Psychoeducation; PT = some other form of psychotherapy, psychological treatment, or counseling; RGT = relaxation group therapy; SAS-SR = Social Adjustment Scale Self-Report; SCID-I = Structured Clinical Interview for DSM–IV; SE = Sweden; TAU = treatment-as-usual; UK = United Kingdom; USA = United States of America; wk = weeks; WHOQOL-BREF = World Health Organization Quality of Life Questionnaire – BREF.  ^a^ RoB abbreviations using the Rob-2 tool: + = low; - = high; +/- = some concerns.  ^b^ HDRS-17 scores are transformed from other scales when unavailable.  ^c^ Participants allocated to placebo antidepressant medication (ADM) or ADM tapering, were excluded from all analyses in the current IPD-MA.  ^d^ CT or other psychosocial interventions explicitly prohibited. | | | | | | | | | | | | | | |  |  |

| Supplementary Table 5. Demographic and clinical variables as predictor of relapse in the control group. | | | | | | |
| --- | --- | --- | --- | --- | --- | --- |
| **Predictor** | **Bivariable model** | | | **Multivariable model** | | |
|  | **HR (95% CI)** | ***p*** | ***n***  **(*n* relapses)** | **HR (95% CI)** | ***p*** | ***n***  **(*n* relapses)** |
| Age | 1.01 (1.00–1.02) | .173 | 328 (171) | n.s. | n.s. | n.s. |
| Gender, male | 1.01 (0.71–1.43) | .977 | 328 (171) | n.s. | n.s. | n.s. |
| Education level, higher | 0.88 (0.55–1.41) | .594 | 311 (163) | n.s. | n.s. | n.s. |
| Marital status, DSW | 1.07 (0.76–1.52) | .693 | 304 (154) | n.s. | n.s. | n.s. |
| Previous episodes, number | 1.01 (1.00–1.03) | .138 | 288 (140) | n.s. | n.s. | n.s. |
| Previous episodes,  3 or more | 1.63 (1.03–2.58) | .036 | 288 (140) | 2.17 (1.18–3.98) | .012 | 210 (98) |
| Age of onset | 1.00 (0.99–1.02) | .856 | 260 (132) | n.s. | n.s. | n.s. |
| Depressive symptoms, HDRS-17 ^a^ | 1.05 (1.01–1.09) | .016 | 328 (171) | 1.22 (1.06–1.40) | .005 | 210 (98) |
| Depressive symptoms, BDI-II | 1.02 (1.00–1.04) | .088 | 250 (129) | 0.94 (0.87–1.01) | .070 | 210 (98) |

| Supplementary Table 6. Moderator analysis of treatment efficacy on time-to-relapse at 12 months, following partial remission from major depressive disorder. | | | | | | |
| --- | --- | --- | --- | --- | --- | --- |
|  | **Psychological intervention vs. non-psychological control** | | | **Psychological intervention plus TAU vs. TAU** | | |
| **Moderator** | **HR (95% CI)**  **Interaction model** | ***p*** | ***n***  **(*n* relapses)** | **HR (95% CI)**  **interaction model** | ***p*** | ***n***  **(*n* relapses)** |
| Age | 0.98 (0.96–1.00) | .074 | 700 (309) | 1.03 (0.96–1.05) | .409 | 401 (205) |
| Gender, male | 0.79 (0.46–1.36) | .399 | 700 (308) | 0.36 (0.07–1.96) | .239 | 402 (205) |
| Education level, higher | 1.41 (0.64–3.13) | .393 | 664 (295) | 1.10 (0.37–3.30) | .860 | 389 (198) |
| Marital status, DSW | 0.87 (0.53–1.43) | .591 | 653 (277) | 1.06 (0.60–1.87) | .849 | 398 (203) |
| Previous episodes, number | 1.00 (0.98–1.02) | .961 | 620 (262) | 1.00 (0.94–1.07) | .984 | 326 (163) |
| Previous episodes, 3 or more | 0.70 (0.36–1.38) | .308 | 620 (262) | 0.53 (0.24–1.18) | .122 | 326 (163) |
| Age of onset | 1.00 (0.97–1.02) | .713 | 560 (242) | 0.99 (0.97–1.02) | .455 | 388 (196) |
| Depressive symptoms, HDRS-17 ^a^ | 0.97 (0.92–1.04) | .405 | 701 (309) | 0.99 (0.92–1.06) | .706 | 402 (205) |
| Depressive symptoms, BDI | 1.00 (0.97–1.03) | .991 | 556 (247) | 1.00 (0.97–1.04) | .893 | 326 (170) |
| *Abbreviations*. BDI-II = Beck Depression Inventory; HDRS-17 = 17-item Hamilton Depression Rating Scale; DSW = divorced, single/separated, or widowed; TAU = treatment as usual; vs = versus.  ^a^ HDRS-17 scores are transformed from other scales when unavailable. | | | | | | |

| Supplementary Table 7. Moderator analysis of treatment efficacy on depressive symptom severity at post-treatment, following partial remission of major depressive disorder. | | | | | | |
| --- | --- | --- | --- | --- | --- | --- |
|  | **Psychological intervention vs. non-psychological control** | | | **Psychological intervention plus TAU vs. TAU** | | |
| **Moderator** | **Mean β (SE)**  **Interaction model** | ***p*** | ***n* observations**  **(*k* studies)** | **Mean β (SE) Interaction model** | ***p*** | ***n* observations**  **(*k* studies)** |
| Age | -0.01 (0.01) | .149 | 599 (15) | 0.00 (0.01) | .659 | 353 (7) |
| Gender, male | -0.28 (0.19) | .140 | 599 (15) | -0.41 (0.25) | .104 | 353 (7) |
| Education level, higher | 0.06 (0.25) | .822 | 569 (14) | -0.25 (0.36) | .495 | 340 (7) |
| Marital status, DSW | -0.49 (0.16) | .002 | 550 (14) | -0.59 (0.21) | .004 | 349 (7) |
| Previous episodes, number | -0.01 (0.01) | .148 | 520 (14) | 0.02 (0.03) | .425 | 279 (6) |
| Previous episodes, 3 or more | 0.05 (0.21) | .823 | 520 (14) | -0.07 (0.27) | .797 | 279 (6) |
| Age of onset | 0.00 (0.01) | .965 | 464 (12) | 0.01 (0.01) | .129 | 340 (7) |
| Baseline depressive symptoms, HDRS-17 ^a^ | -0.01 (0.02) | .715 | 600 (15) | -0.01 (0.02) | .613 | 353 (7) |
| Baseline depressive symptoms, BDI | 0.00 (0.01) | .936 | 467 (10) | -0.01 (0.01) | .731 | 378 (5) |
| *Abbreviations*. BDI = Beck Depression Inventory; HDRS-17 = 17-item Hamilton Depression Rating Scale; DSW = divorced, single/separated, or widowed; vs = versus.  ^a^ HDRS-17 scores are transformed from other scales when unavailable. | | | | | | |

| Supplementary Table 8. Moderator analysis of treatment efficacy on depressive symptom severity at 60 weeks, following partial remission of major depressive disorder. | | | | | | |
| --- | --- | --- | --- | --- | --- | --- |
|  | **Psychological intervention vs. non-psychological control** | | | **Psychological intervention plus TAU vs. TAU** | | |
| **Moderator** | **Mean β (SE)**  **Interaction model** | ***p*** | ***n* observations**  **(*k* studies)** | **Mean β (SE)**  **Interaction model** | ***p*** | ***n* observations**  **(*k* studies)** |
| Age | -0.01 (0.01) | .048 | 543 (14) | -0.02 (0.01) | .135 | 338 (7) |
| Gender, male | -0.52 (0.20) | .009 | 519 (14) | -0.71 (0.27) | .007 | 338 (7) |
| Education level, higher | 0.06 (0.27) | .818 | 521 (13) | -0.17 (0.39) | .664 | 329 (7) |
| Marital status, DSW | -0.14 (0.17) | .417 | 494 (13) | -0.17 (0.22) | .432 | 334 (7) |
| Previous episodes, number | -0.01 (0.02) | .446 | 473 (13) | 0.02 (0.03) | .567 | 273 (6) |
| Previous episodes, 3 or more | -0.02 (0.22) | .933 | 473 (13) | 0.07 (0.30) | .813 | 273 (6) |
| Age of onset | 0.00 (0.01) | .837 | 440 (12) | 0.01 (0.01) | .533 | 324 (7) |
| Baseline depressive symptoms, HDRS-17 ^a^ | -0.05 (0.02) | .047 | 544 (14) | -0.06 (0.03) | .017 | 338 (7) |
| Baseline depressive symptoms, BDI | -0.02 (0.01) | .074 | 423 (9) | -0.03 (0.02) | .027 | 267 (5) |
| *Abbreviations*. BDI = Beck Depression Inventory; HDRS-17 = 17-item Hamilton Depression Rating Scale; DSW = divorced, single/separated, or widowed; vs = versus.  ^a^ HDRS-17 scores are transformed from other scales when unavailable. | | | | | | |

# Supplementary Figure 1.

# *Forest Plot of Random Effects Two-Stage Individual Participant Data Meta-Analysis for Depression Severity at Post-treatment and 60-weeks Follow-up, Comparing Psychological with Non-psychological Interventions.*


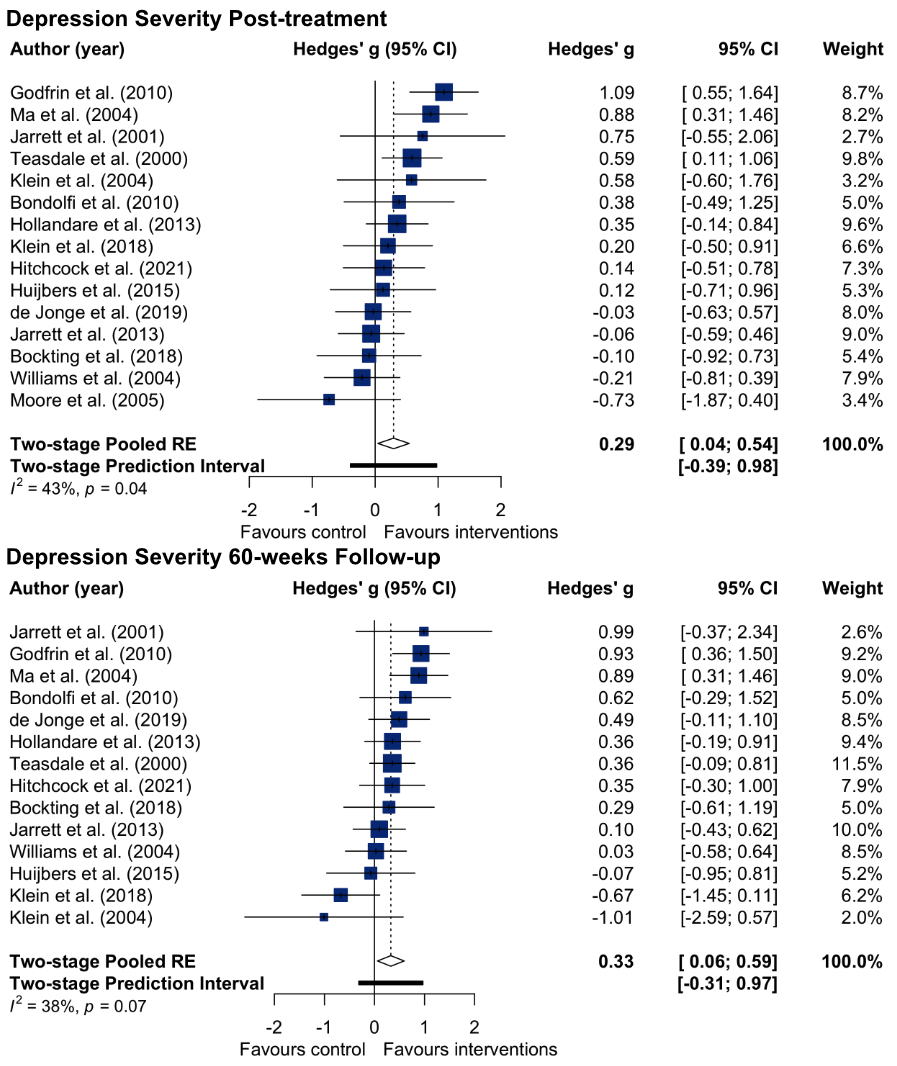


# Supplementary Figure 2.

# *Funnel plot examining small-study effects bias with results from a two-stage IPD meta-analysis model of treatment efficacy on time-to-relapse 12 months.*
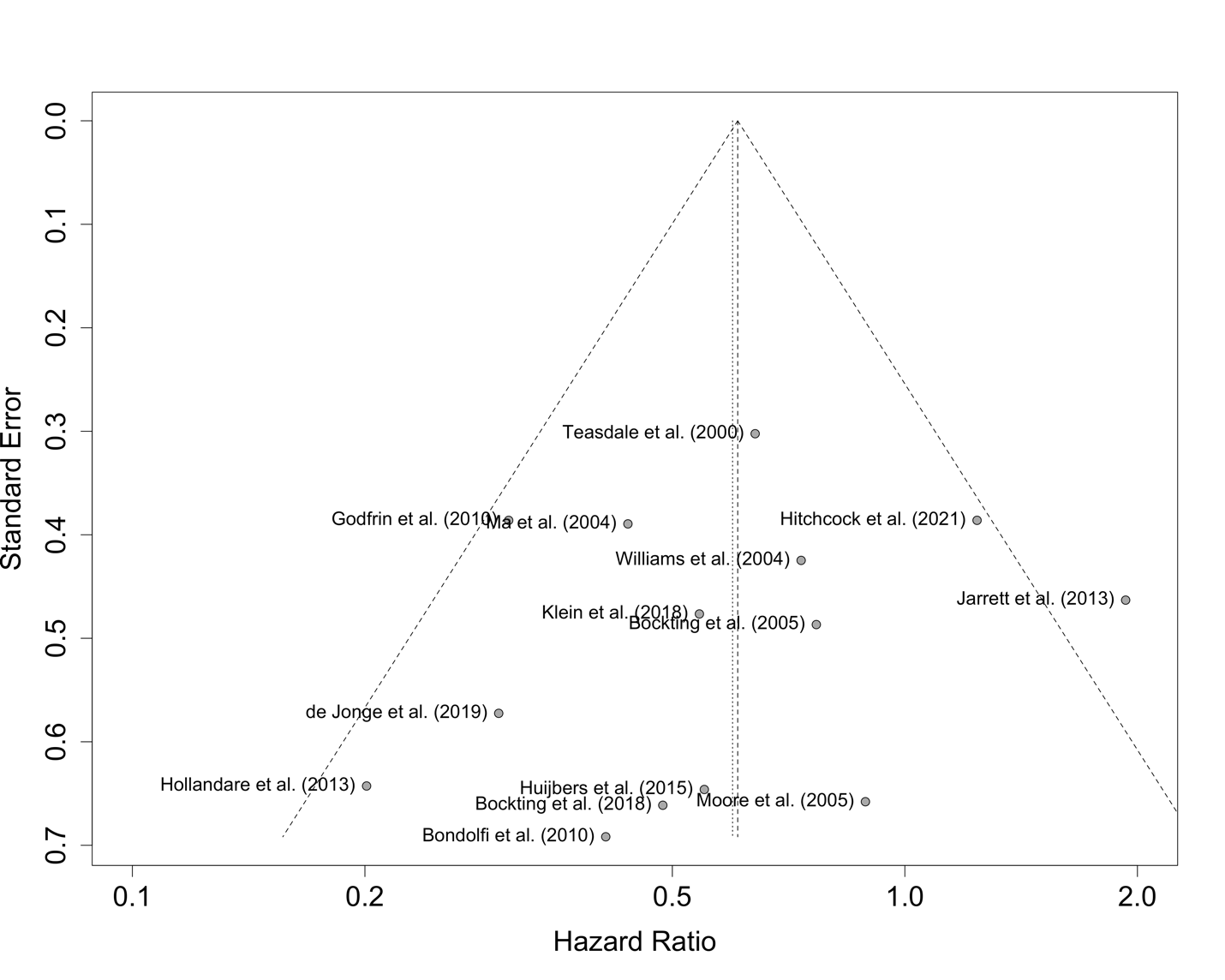


# Supplementary Figure 3.

# *Funnel plot examining small-study effects bias with results from a two-stage IPD meta-analysis model of treatment efficacy on depressive symptom severity at post-treatment.*


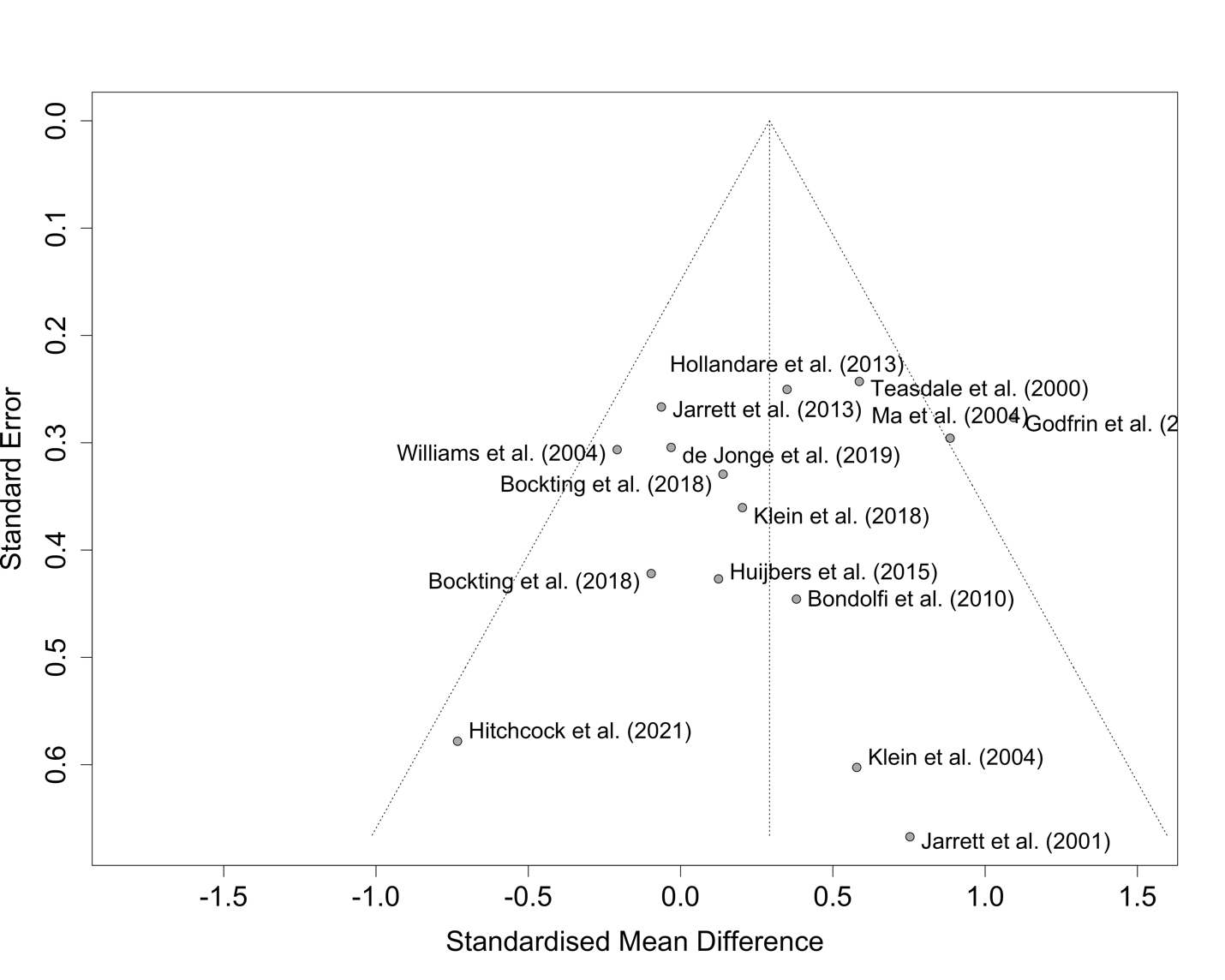


# Supplementary Figure 4.

# *Funnel plot examining small-study effects bias with results from a two-stage IPD meta-analysis model of treatment efficacy on depressive symptom severity at 60-weeks follow-up.*


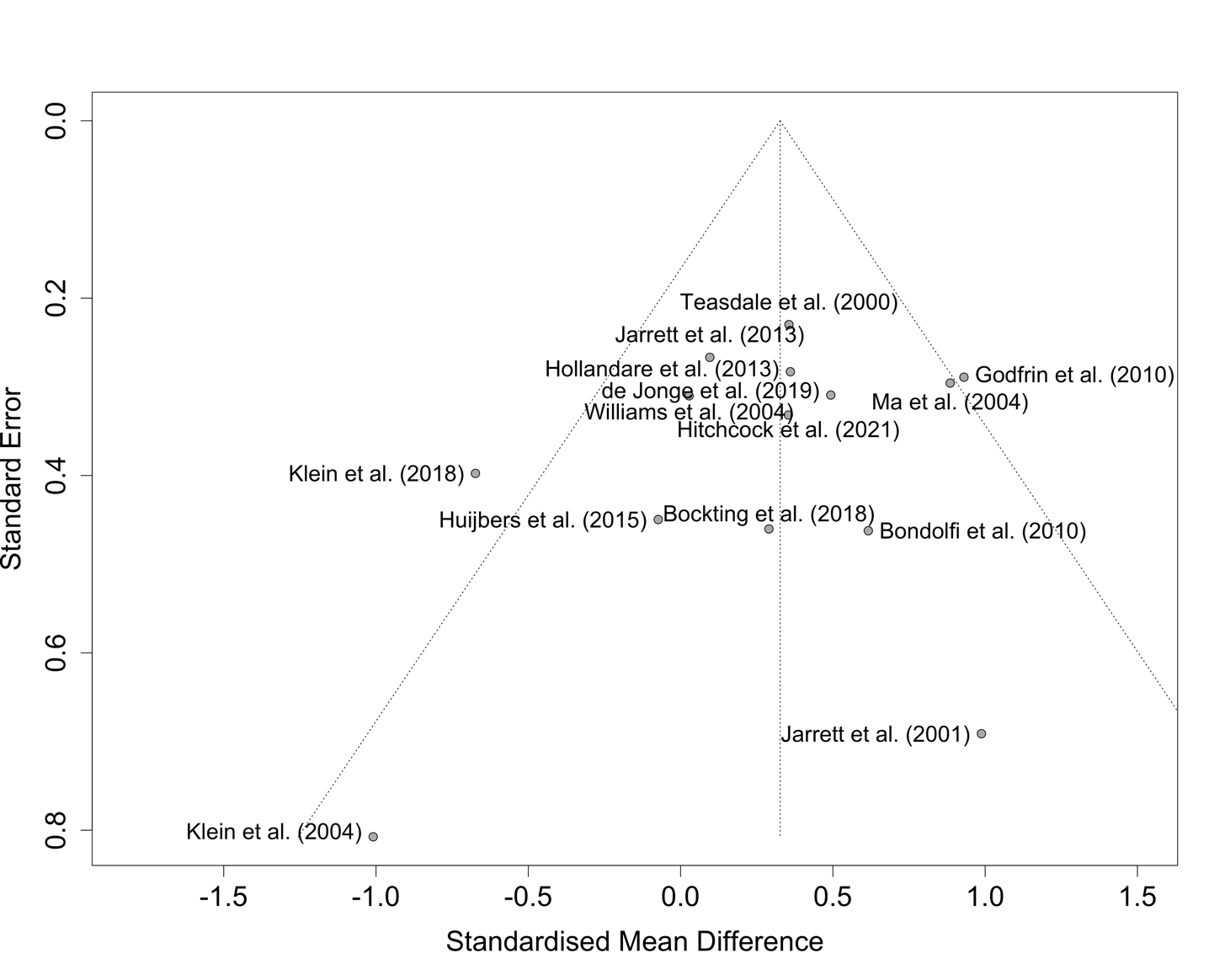


# Supplementary Figure 5.

# *Risk of bias plot using the Cochrane Risk-of-Bias 2 tool of included randomized controlled trials, across 5 domains and overall.*


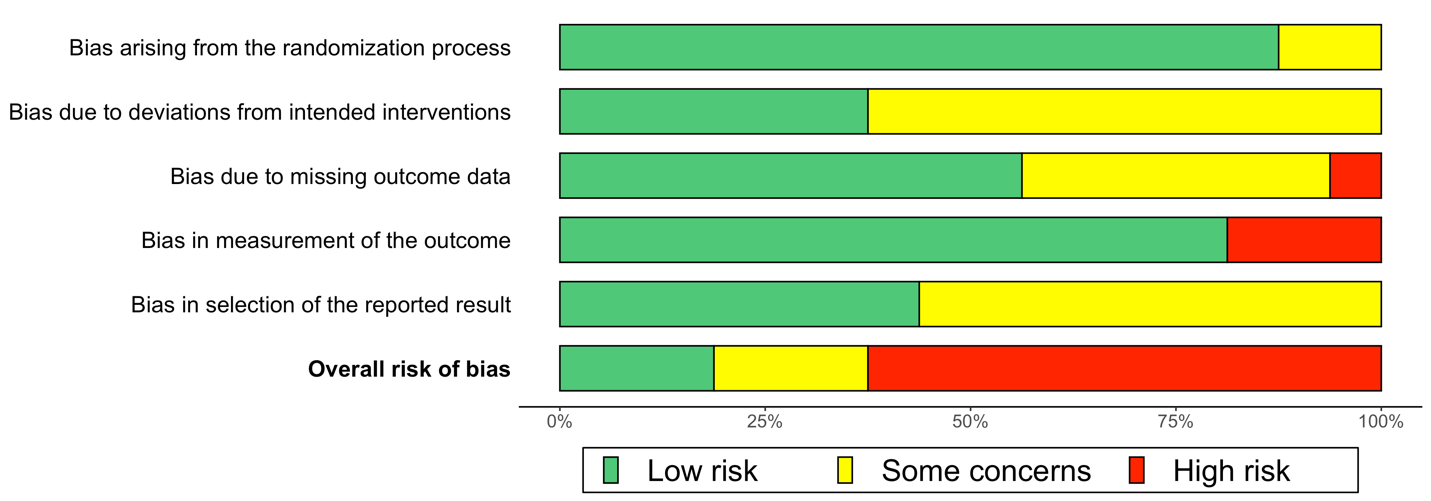

Supplement: Gülpen et al. supplementary material [file S0033291725000157sup001.docx]
